# Design, Synthesis, Antitumor Activity and Molecular Docking Study of Novel 5-Deazaalloxazine Analogs

**DOI:** 10.3390/molecules25112518

**Published:** 2020-05-28

**Authors:** Sawsan Mahmoud, Doaa Samaha, Mosaad S. Mohamed, Nageh A. Abou Taleb, Mohamed A. Elsawy, Tomohisa Nagamatsu, Hamed I. Ali

**Affiliations:** 1 Pharmaceutical Chemistry Department, Faculty of Pharmacy, Helwan University, Helwan 11795, Egypt; sawsansf@yahoo.com (S.M.); samahado@student.hu-berlin.de (D.S.); dr.nagehabotaleb@yahoo.com (N.A.A.T.); 2 Institute of Chemistry, Humboldt-Universität zu Berlin, Brook-Taylor-Str. 2, 12489 Berlin, Germany; 3 Pharmaceutical Organic Chemistry Department, Faculty of Pharmacy, Helwan University, Helwan 11795, Egypt; doctormosaad@hotmail.com; 4 School of Pharmacy, Queen’s University of Belfast, 97 Lisburn Road, Belfast BT9 7BL, UK; melsawy01@qub.ac.uk; 5 Leicester Institute of Pharmaceutical Innovation, Leicester School of Pharmacy, De Monfort University, The Gateway, Leicester LE1 9BH, UK; 6 Department of Medical Technology, Faculty of Health Science, Kumamoto Health Science University, Kumamoto 861-5598, Japan; nagamatu@kumamoto-hsu.ac.jp; 7 Irma Lerma Rangel College of Pharmacy, Texas A&M University, College Station, Kingsville, TX 78363, USA

**Keywords:** deazaalloxazine, antitumor, AutoDock, selectivity, protein kinase

## Abstract

Protein tyrosine kinases (PTKs) are the most potential therapeutic targets for cancer. Herein, we present a sound rationale for synthesis of a series of novel 2-(methylthio), 2-(substituted alkylamino), 2-(heterocyclic substituted), 2-amino, 2,4-dioxo and 2-deoxo-5-deazaalloxazine derivatives by applying structure-based drug design (SBDD) using AutoDock 4.2. Their antitumor activities against human CCRF-HSB-2, KB, MCF-7 and HeLa have been investigated in vitro. Many 5-deazaalloxazine analogs revealed high selective activities against MCF-7 tumor cell lines (IC_50_: 0.17–2.17 µM) over HeLa tumor cell lines (IC_50_ > 100 µM). Protein kinase profiling revealed that compound **3h** induced multi- targets kinase inhibition including −43% against (FAK), −40% against (CDKI) and −36% against (SCR). Moreover, the Annexin-V/PI apoptotic assay elucidate that compound 3h showed 33% and potentially 140% increase in early and late apoptosis to MCF-7 cells respectively, compared to the control. The structure-activity relationship (SAR) and molecular docking study using PTK as a target enzyme for the synthesized 7-deazaalloaxazine derivatives were investigated as potential antitumor agents. The AutoDock binding affinities of the 5-deazaalloxazine analogs into c-kit PTK (PDB code: 1t46) revealed reasonable correlations between their AutoDock binding free energy and IC_50_.

## 1. Introduction

Cancer is a severe health problem in the United States. In 2020, there have been more than 1.8 million new cases diagnosed with cancer and nearly 606,520 died from it in the US [1]. The implication of protein tyrosine kinases (PTKs in various forms of cancers made them significant targets in drug discovery. The selective PTK inhibitors are prospective candidates as antitumor agents by inhibiting multiple pathways in cancer cells, such as proliferation, survival and angiogenesis [2]. Consequently, the synthesis of small molecules as PTK inhibitors (PTKIs) has developed into a remarkable approach toward new therapeutics [3]. After the Food and Drug Administration (FDA) approval for imatinib in 2001, they become a trend as potential anticancer drugs to the market [4]. Nowadays, there are more than twenty FDA-approved PTKIs for cancer therapy [5].

**Figure 1:**
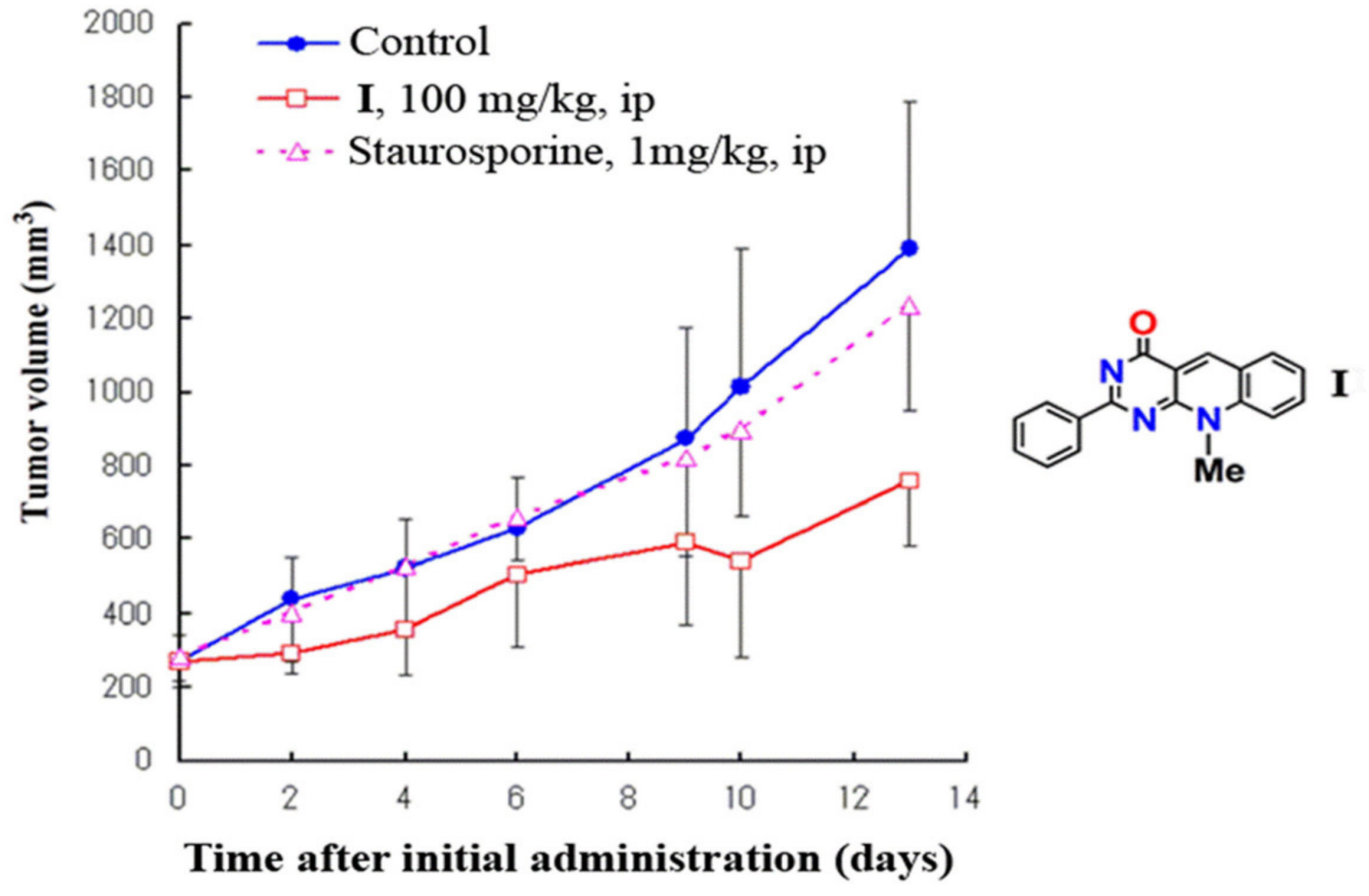
One hundred mg/kg, i.p., of compound I reduced the tumor volume of Subcutaneous (SC) implanted A431 into nude mouse 53.4% after 10 days of 100 mg/kg, ip.

Interestingly, the in vivo antitumor activity of 2-deoxo-10-methyl-2-phenyl- 5-deazaflavin (I) against subcutaneously transplanted human adenocarcinoma A431 xenograft into BALB/c nude mice was reported early by our research group [6]. In this study, the tumor volume was reduced to 53.4% of the intraperitoneal administration after 10 days (100 mg/kg; i.p.) as shown in Figure 1. Furthermore, we investigated the antitumor activities of 5-deazaflavins and flavins derivatives against a variety of cancer cell lines and elucidated that these candidates could be promising antitumor drugs [7,8].

**Figure 2:**
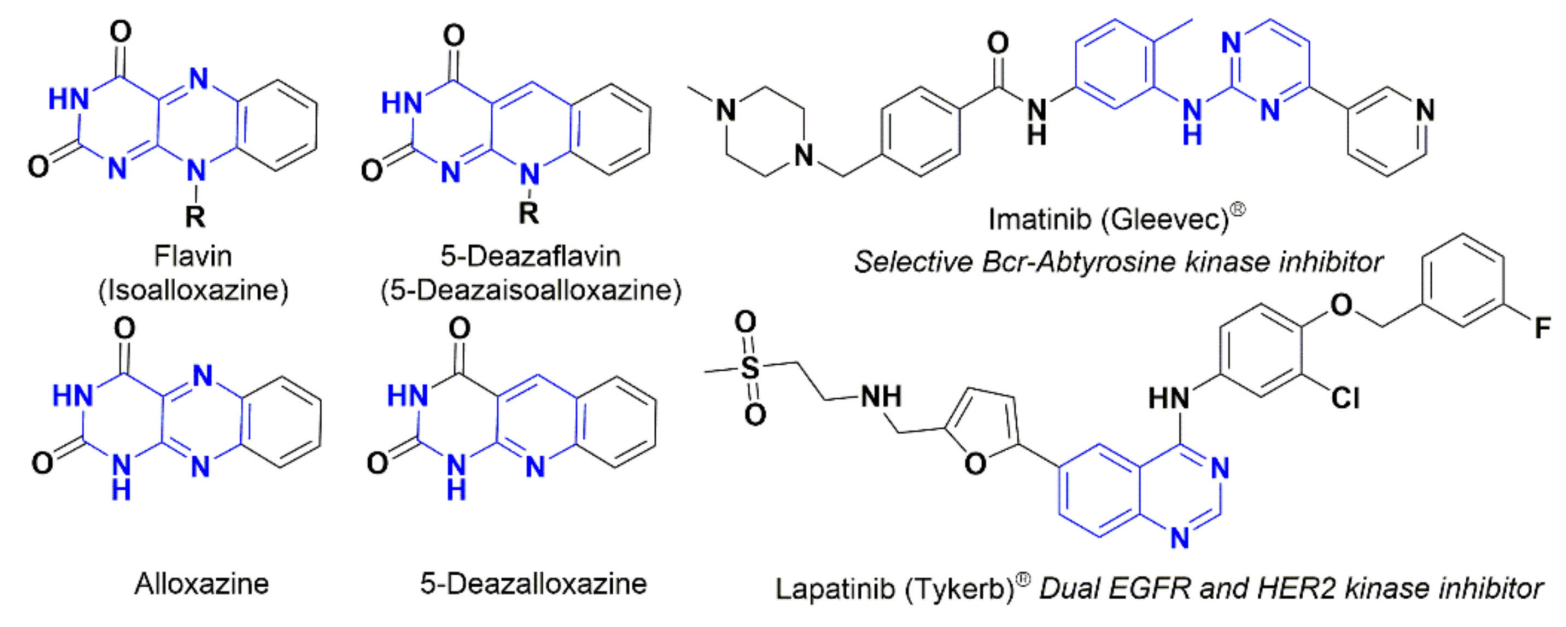
Structures of flavin, 5-deazaflavin, alloxazine, 5-deazaalloxazins and the well-known kinase inhibitors (imatinib and lapatinib).

The potential antitumor activity of 5-deazaalloxazines {pyrimido[4,5-*b*] quinoline-2,4(1*H*, 3*H*)-diones} is attributed to their structural similarities with the scaffold of 5-deazaflavins {pyrimido[4,5-*b*] quinoline-2,4(3*H*, 10*H*)-diones} [9]. The condensed pyrimido[4,5-*b*] quinoxaline cyclic systems are called "alloxazines” which exist in two tautomeric forms, one of which, when "frozen" by substitution of the hydrogen atom at N (10), is the source of the isoalloxazine series [10] as shown in Figure 2.

**Figure 3:**
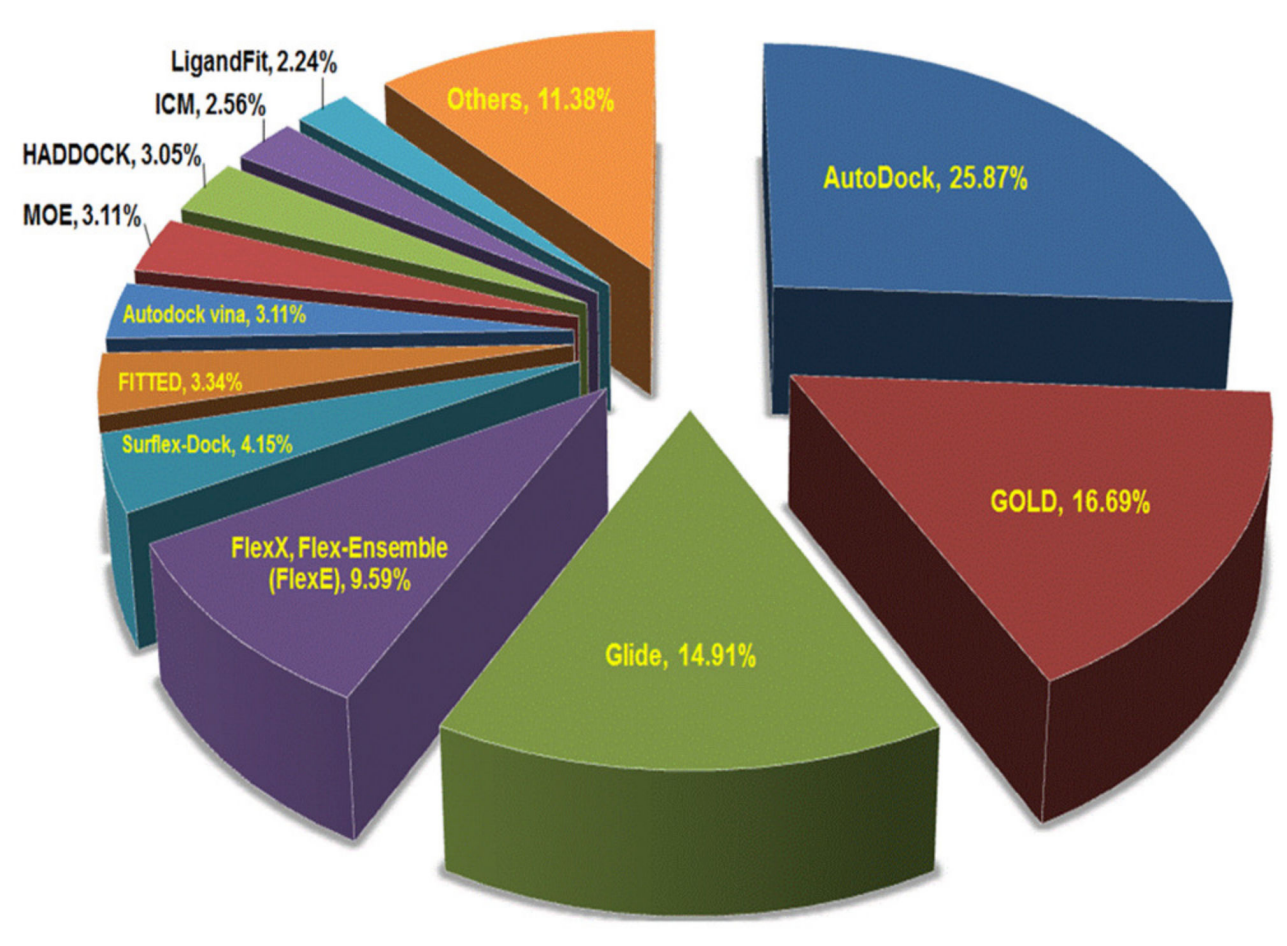
The rate of citation of different molecular docking programs, in which Autodock, GOLD and Glide are the most commonly used ones, modified through reference [21].

Computer-aided drug design (CADD) is a powerful model for drug discovery, where several computationally designed drugs are currently under clinical investigations and even marketed, such as Dorzolamide, Saquinavir, Aliskiren [11,12]. Recent applications of computational chemistry comprising both Structure-Based Drug Design (SBDD) and Ligand-Based Drug Design (LBDD) approaches in the discovery and design of kinase inhibitors were intensively implemented in the design and discovery of many kinase inhibitors such as imatinib (Figure 2) [13,14]. Multi-targeted therapy is a powerful category for eradication of cancer cells; therefore, CADD tools are fundamental in the discovery and development of anticancer drugs [15,16,17]. SBDD techniques such as homology modeling, molecular dynamics (MD) simulations and docking have been applied for lead optimization based on the binding sites of therapeutic target proteins [18,19,20]. Molecular docking is the most advanced technique for lead optimization by investigation of the binding affinities of compounds into the binding cavities of the therapeutic target protein. AutoDock is the most commonly cited software docking program in the scientific literature Figure 3 [21]. The docking binding affinity studies for many 5-deazaflavin derivatives into PTK have been reported [10,22,23,24].

For the aforementioned facts, new derivatives of flavin and deazaflavin derivatives compounds are still in demand to enhance their binding affinity into the targeted kinases and consequently their and the antitumor activities. Moreover, our earlier results [7] have identified several key features through Structure Activity Relationship (SAR) study of the different flavin analogs to impact the maximum binding affinity by the following structural characteristics: (1) no substituent at the N-10 position on 5-deazaflavins, flavins and flavin-5-oxide analogs, (2) hydrogen at the N-3 position, (3) Ph or NH_2_ group at the C-2 position. These preliminary studies prompted us to synthesize new 5-deazaalloxazines analogs based on SBDD for structural optimization of our reported compounds to reveal higher selectivity against different kinases and tumor cell lines.

## 2. Results and Discussion

### 2.1. Synthesis and Structural Elucidation of 5-Deazaalloxazine Derivatives

**Scheme 1:**
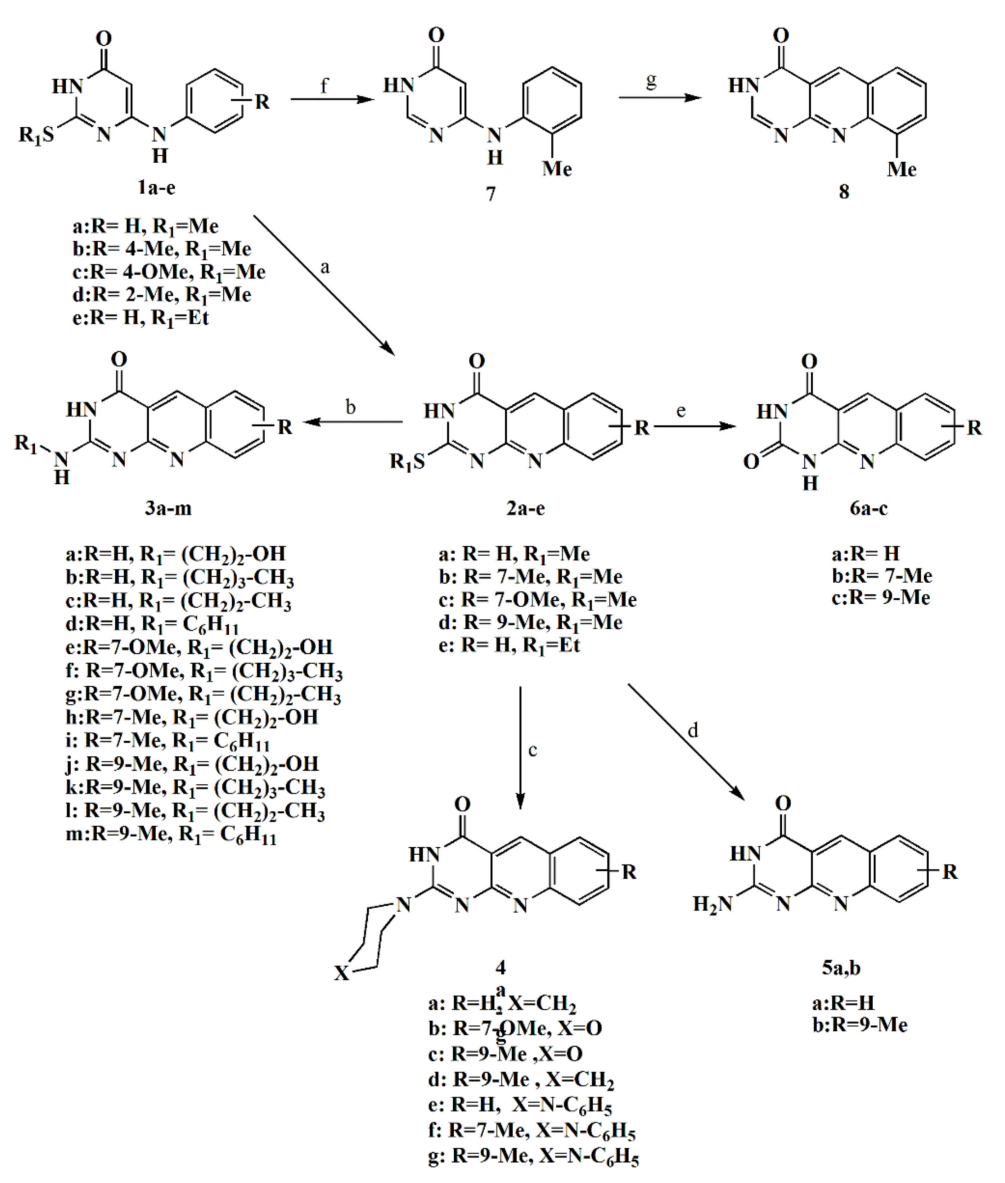
General methods for the preparation of 2-deoxo-2-alkylthio-5-deazaalloxazines (**2a**–**e**), 2-(substituted alkyl amino)-2-deoxo-5-deazaalloxazines (**3a**–**m**), 2-(heterocyclic substituted)-2-deoxo-5-deazaalloxazines (**4a–g**), 2-amino-2-deoxo-5-deazaalloxazines (**5a,b**), 2,4-dioxo-5-deazaalloxzines (**6a**–**c**), 2-deoxo-5-deazaalloxazine (**8**). Reagents and conditions*:* (**a**) DMF-POCl_3_, 90 °C, 1–4 h; (**b**) R_1_NH_2_, DMF, reflux 7–9 h; (**c**) 2^ry^ amine, DMF, reflux 24–30 h; (**d**) CH_3_COONH_4_, 160–165 °C, 0.5–1.5 h; (**e**) 5 M HCl, reflux, 15 h; (**f**) Raney Ni, absolute ethanol, 0.5 h; (**g**) DMF-POCl_3_, 90 °C, 2 h.

The prerequisite starting material, 6-(*N*-alkylanilino)-2-methylthiopyrimidin- 4(3H)-ones (**1a**–**e**) were prepared by alkylation of 2,3-dihydro-6-(*N*-arylamino)-2-thioxopyrimidin-4(1*H*)-ones [8] with alkyl iodide in 2 M NaOH at 0–5 °C for 30 min in 61–90% yields [21]. The intermediate compounds, 2-deoxo-2-alkylthio-5- deazaalloxazines (**2a**–**e**) were prepared according to the reported method [10,13] by the reaction of 6-(*N*-alkylanilino)-2-alkylthiopyrimidin-4(3*H*)-ones (**1a**–**e**) with Vilsmier reagent (dimethylformamide-phosphorus oxycholride-DMF-POCl_3_) at 90 °C for 1–4 h in 72–86% yields. Amination of 2-alkylthiopyrimidine and 5-deazaalloxazine analogs via reaction with different alkyl and aryl amines has long been investigated [8,25,26]. The carbons adjacent to nitrogens of pyrimidine moieties of 5-deazaalloaxzine are susceptible to nucleophilic substitution reactions because of the π electron-deficient nature. Thus, the different amination reactions have been done by substitution of 2-methylthio group with different amines for preparation of 2-(substituted alkylamino)-, 2-(heterocyclic substituted)- and 2-amino-2-deoxo-5-deazaalloxazines {pyrimido[4,5-*b*] quinoline-4(3*H*)-one derivatives} (**3a**–**m**, **4a**–**g** and **5a,b**) as represented in Scheme 1. These derivatives (**3a**–**m**, **4a**–**g** and **5a,b**) have higher aqueous solubility because of their polar side chain bearing different amino groups at position 2, which may be the predisposing factor for boosting their potency and selectivity.

2-(Substituted alkyl amino)-2-deoxo-5-deazaalloxazines (**3a**–**m**) were synthesized by heating under reflux the compounds 2-deoxo-2-alkylthio- 5-deazaalloxazines (**2**) with an appropriate primary amine including ethanolamine, *n*-butylamine, *n*-propylamine, and/or cyclohexylamine for 7–9 h. The products (**3a**–**m**) were obtained as yellow needles in 60–88% yields. Moreover, a variety of substitutions namely, 2-piperidin-1-yl, 2-morpholin-4-yl and 2-piprazin-4-yl of 2-deoxo-5-deazaalloxazines (**4a**–**g**) were prepared by refluxing of the intermediate compounds (**2**) with an appropriate secondary amine including morpholine, piperidine, and/or *N*-phenylpiperazine in dimethylformamide (DMF) for 24–30 h. All the products of (**4a**–**g**) were afforded as needles with bright yellow color in 64–75% yields. Whereas, 2-amino-2-deoxo-5-deazaalloxazines (**5a,b**) were synthesized by fusion of 2-deoxo-2-alkylthio-5-deazaalloxazines (**2**) with ammonium acetate for 0.5–3 h at 160–165 °C to produce the corresponding 5-deazaalloxazines (**5a,b**) as yellow needles in 69–83% yields. The excess amines, in the above reactions involving treatment with volatile amines, were evaporated under vacuo. While, the high boiling point amines such as piperidine, morpholine and *N*-phenylpiperazine were separated from residue by treating with water or ethanol to get amine-free products.

**Figure 4:**
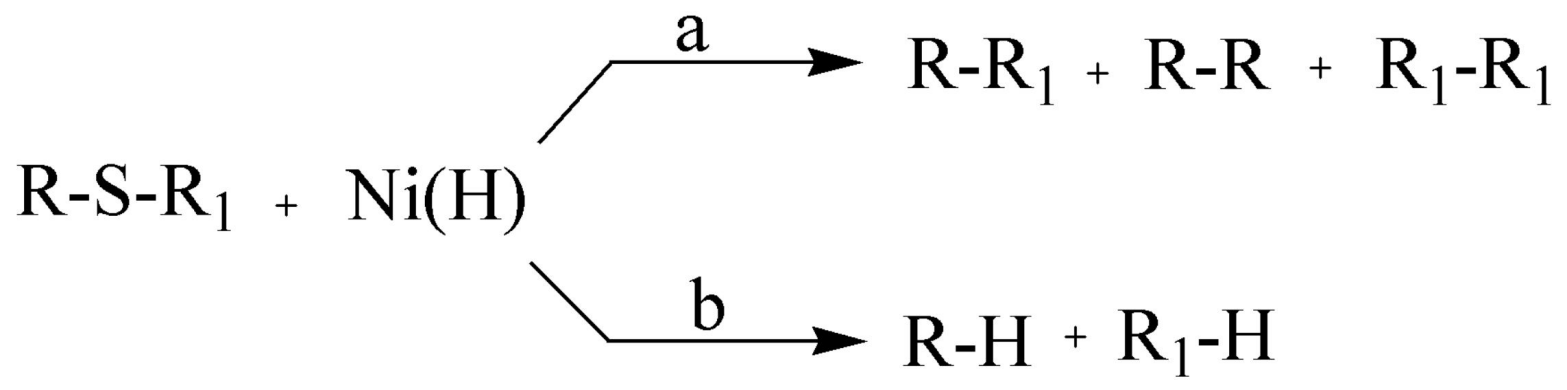
Desulfurization method under Mozingo conditions (**a**) and in the presence of sufficient Raney nickel catalyst (**b**).

Acidic hydrolysis of the intermediate 2-deoxo-2-alkylthio-5-deazaalloxazines (**2**) in 5 M hydrochloric acid by heating under reflux for 10–15 h produces the corresponding 2,4-dioxo-5-deazaalloxzines (**6a**–**c**) as sharp yellow needles in 62–84% yields. Desulfurization of the alkyl mercapto substituent is often a critical step in heterocyclic synthesis, particularly in pyrimidine chemistry [27]. The most commonly used method of desulfurization is to reflux the target compound with an excess of Raney nickel (Mozingo conditions) [28]. According to Mozingo, R. et al., two courses for the reaction of sulfide may be postulated. In the first of these, the nickel is considered to function as a metal removing the sulfur in the Wurtz-type reaction, Figure 4a. Alternatively, in the presence of sufficient Raney nickel catalyst, to contain an excess of hydrogen, the reaction may take the course represented by Figure 4b.

In the desulfurization reaction under Mozingo conditions, using aliphatic and aromatic sulfides, disulfides, sulfoxides and sulfones with sufficient Raney nickel catalyst only the reaction (b) has been observed. That is, in case, the rupture of the C-S bond was accompanied by the formation of a new C-H bond and a combination of the organic radicals did not occur. Other methods for the replacement of the mercapto group by hydrogen include oxidation with nitric acid or with hydrogen peroxide in acidic solution [27]. In this study, the 2-unsubstituted-2-deoxo-5-deazaalloxazines derivatives were prepared by dethiation approach of the corresponding 2-methylthio analogs. The required 6-(*o*-tolylamino) pyrimidin-4(3*H*)-one (**7**) was prepared by dethiation of 6-(*N*-*o*-tolylamino)-2-methylthiopyrimidin-4(3*H*)-one (**1d**) by using an excess of Raney nickel and heating under reflux in ethanol for 0.5 h to afford 70% yield. The dethiated 5-deazaalloxazine analog, namely, 9-methylpyrimido[4,5-*b*] quinolin-4-ol (**8**), was synthesized according to the reported procedure [8] by the reaction of 6-(*o*-tolylamino) pyrimidin-4(3*H*)-one (**7**) with Vilsmier reagent (DMF-POCl_3_) at 90 °C for 2 h to produce pale yellow needles in 80% yield, as represented in Scheme 1.

The UV/VIS, ^1^H-NMR, mass spectra, IR and elemental analysis were applied to determine and identify the synthesized compounds. ^1^H-NMR spectra proved the intermediate compounds (**1a**–**d**) by the presence of a proton resonance as a singlet signal at 5-position at 5.04–5.51 ppm, as reported [8]. The cyclized 2-deoxo-2-alkylthio-5-deazaalloxazines (**2b**–**e**) exhibited a significant singlet signal because of the proton at C5 in the lower field at 9.01–9.18 ppm. Also, compounds (**2b**–**d**) showed a slight downfield shift of their ^1^H-NMR singlet signal of 2-SMe moieties being at 2.56–2.85 ppm owing to the newly cyclized conjugated tricyclic pyrimido[4,5-*b*] quinolin-4(3*H*)-one ring in comparison with the of uncyclized analog. Additionally, 3-NH singlet signals at ^1^H-NMR spectra were detected at 11.78–12.68 ppm for the 2-SMe cyclized derivatives (**2b**–**d**), whereas, ^1^H-NMR singlet signal of 3-NH for compound **2e** exhibited at an upfield shift of 9.43 ppm. This is because of the 2-ethylthio group has higher electron donating properties than that of the 2-methylthio group (see Supplementary Materials).

2-(Substituted alkyl amino)-2-deoxo-5-deazaalloxazines (**3a**–**m**)*,* 2-(heterocyclic substituted)-2-deoxo-5-deazalloxazines (**4a**–**g**) and 2-amino-2-deoxo- 5-deazaalloxazine derivatives (**5a,b**) were characterized by vanishing the 2-methylthio group singlet signal of their precursors (**2a**–**e**). This is concurrent with the appearance of a singlet signal of the 2-NH at δ_H_: 6.52–6.90 ppm for compounds **3a**–**c**, **e**–**h**, **j**–**l**. While the 2-NH singlet signal of the 2-(cyclohexyl amino) analogs (**3d, i, m**) are located at upfield region δ_H_: 3.93–3.95 ppm owing to the positive inductive effect of cyclohexyl moiety. 2-(Piperiden-1-yl)-2-deoxo*-*5-deazalloxazines (**4a,d**) are elucidated by their 6H multiplets at δ_H_: 1.50–1.66 ppm and 4H multiplets at δ_H_: 3.73–3.82 ppm for the piperidinyl moiety. 2-(Morpholin-4-yl)-2-deoxo*-* 5-deazalloxazines (**4b,c**) are elucidated by their twin 4H multiplets at δ_H_: 3.22–3.29 and 3.70–3.86 ppm for the morpholinyl moiety. Similarly, 2-(4-phenylpiperazin-1-yl)-pyrimido[4,5-*b*] quinoline-4-ol derivatives (**4e**–**g**) are identified by their piperazinyl two 4H multiplets at δ_H_: 3.03–3.34 and 3.93–3.99 ppm.

**Figure 5:**
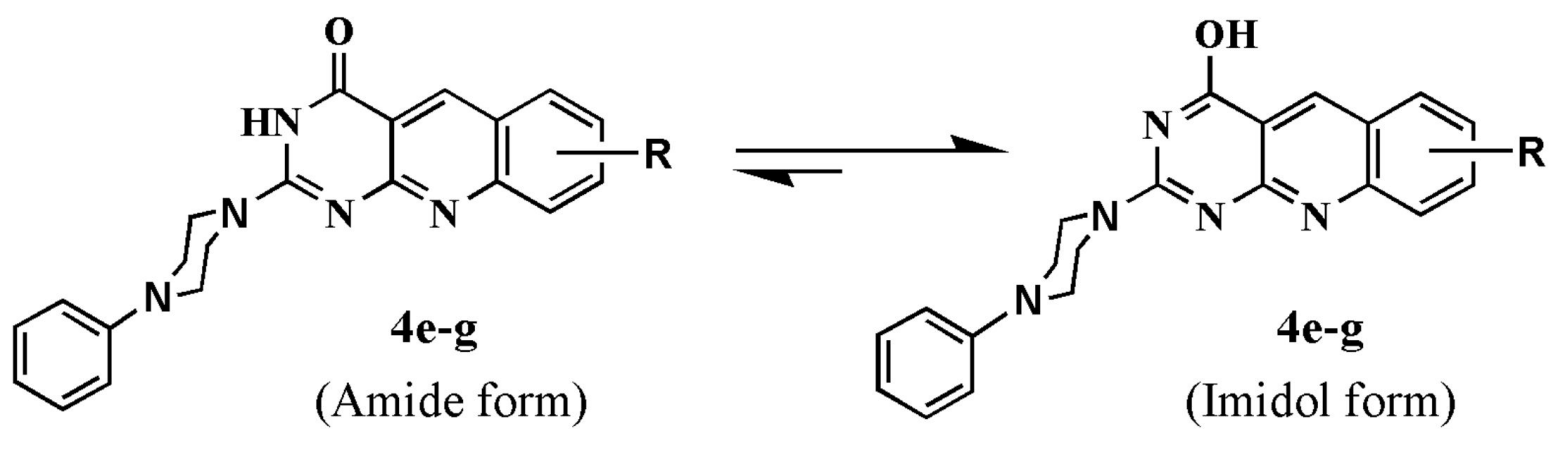
The amide-iminol tautomerism for compounds 2-deoxo-2-(4-phenylpiperazin-1-yl)-5- deazaalloxazines (**4e**–**g**).

Interestingly, the phenomenon of amide-imidol tautomerism was noticed for the ^1^H-NMR spectra for 2-deoxo-2-(4-phenylpiperazin-1-yl)-5-deazaalloaxazines (**4e**–**g**). The imidol tautomer predominates in ^1^H-NMR (DMSO-d_6_ at 25 °C), Figure 5. The characteristic signal of the 4-OH proton was detected at δ_H_: 3.44–4.49 ppm in the ^1^H-NMR spectra concurrent disappearance of the singlet signal of the 3-NH proton. This also confirmed by the disappearance of 3-NH and C=O bands in the IR spectrum and displaced by the OH stretching band at 3421–3434 cm^−1^.

Compounds (**5a,b**) are characterized by appearance of D_2_O exchangeable broad singlet signals at δ_H_: 6.72 and 6.74 ppm belong to exocyclic 2-NH_2_ and broad singlet signals of 3-NH at δ_H_: 10.81 and 11.18 ppm, respectively.

The ^1^H-NMR spectra of 2,4-dioxo-5-deazaalloxzines (**6a**–**c**), were elucidated by their two characteristic D_2_O exchangeable broad singlet signals at δ_H_: 10.93–11.49 and 11.34–11.76 ppm which are corresponding to 1-NH and 3-NH, respectively. Two strong IR peaks at νcm^−1^ 1712–1703 and 1625–1615 cm^−1^ boosted their structural elucidation.

**Figure 6:**
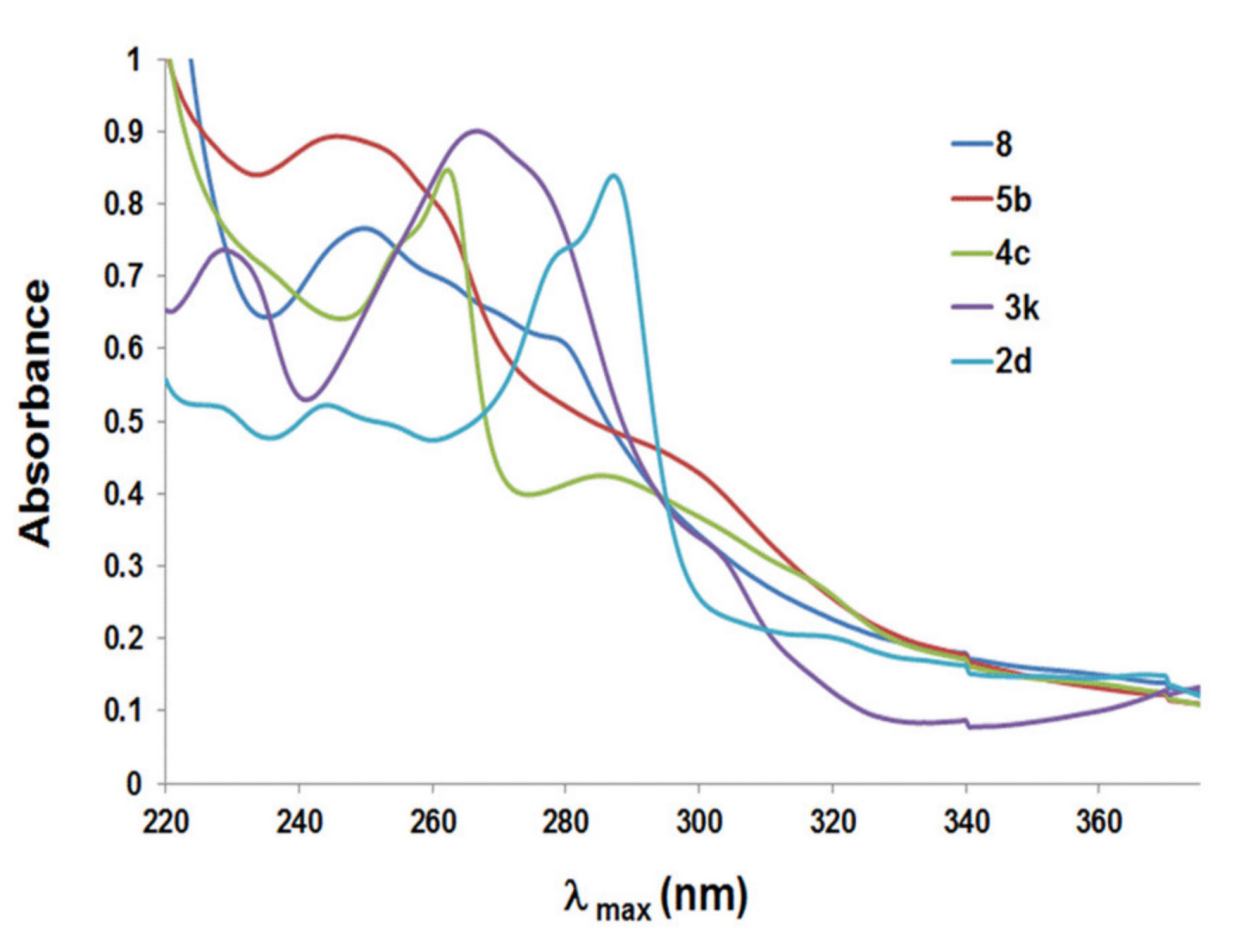
UV–VIS spectra of 9-methyl-2-methylthio-5-deazaalloxazine (**2d**), 2-butylamino-9-methyl-5-deazaalloxazine (**3k**)*,* 9-methyl-2-(morpholin-4-yl)-5-deazaalloxazine (**4c**), 2-amino-9-methyl-5-deazaalloxazine (**5b**) and 2-deoxo-5-deazaalloxazine (**8**).

In general, the UV spectra of 2-deoxo-2-alkylthio-5-deazalloxazines (**2a**–**e**) showed longer wavelength than those of compounds **3a**–**m**, **4a**–**g**, **5a**, **b**, **6a**–**c** and **8** as shown in Figure 6. This redshift (bathochromic shift) is attributed to the easier polarizability of the S-atom in 2-alkylthio analogs (**2a**–**e**) [29]. The UV/VIS absorption spectra of compounds (**4b**–**e**) exhibited three absorption maxima at 230–254, 284–287 and 362–401 nm with one absorption shoulder at 266–279 nm. While, the UV/VIS absorption spectra of 7-methoxy2-(substituted alkyl amino)-2-deoxo-5-deazaalloxazines (**3f,g**) show four absorption maxima at 224–230, 267–277, 327–370, 394–401 nm without any absorption shoulder. While the other 2-(substituted alkyl amino)-2-deoxo-5-deazaalloxazines (**3a**–**e**, **h**–**m**), 2-(heterocyclic substituted)-2-deoxo-5-deazaalloxazines (**4a**–**g**) and 2-amino-2-deoxo-5-deazaalloxazines (**5a,b**) show three absorption maxima together with an absorption shoulder as shown in Figure 6.

**Scheme 2:**
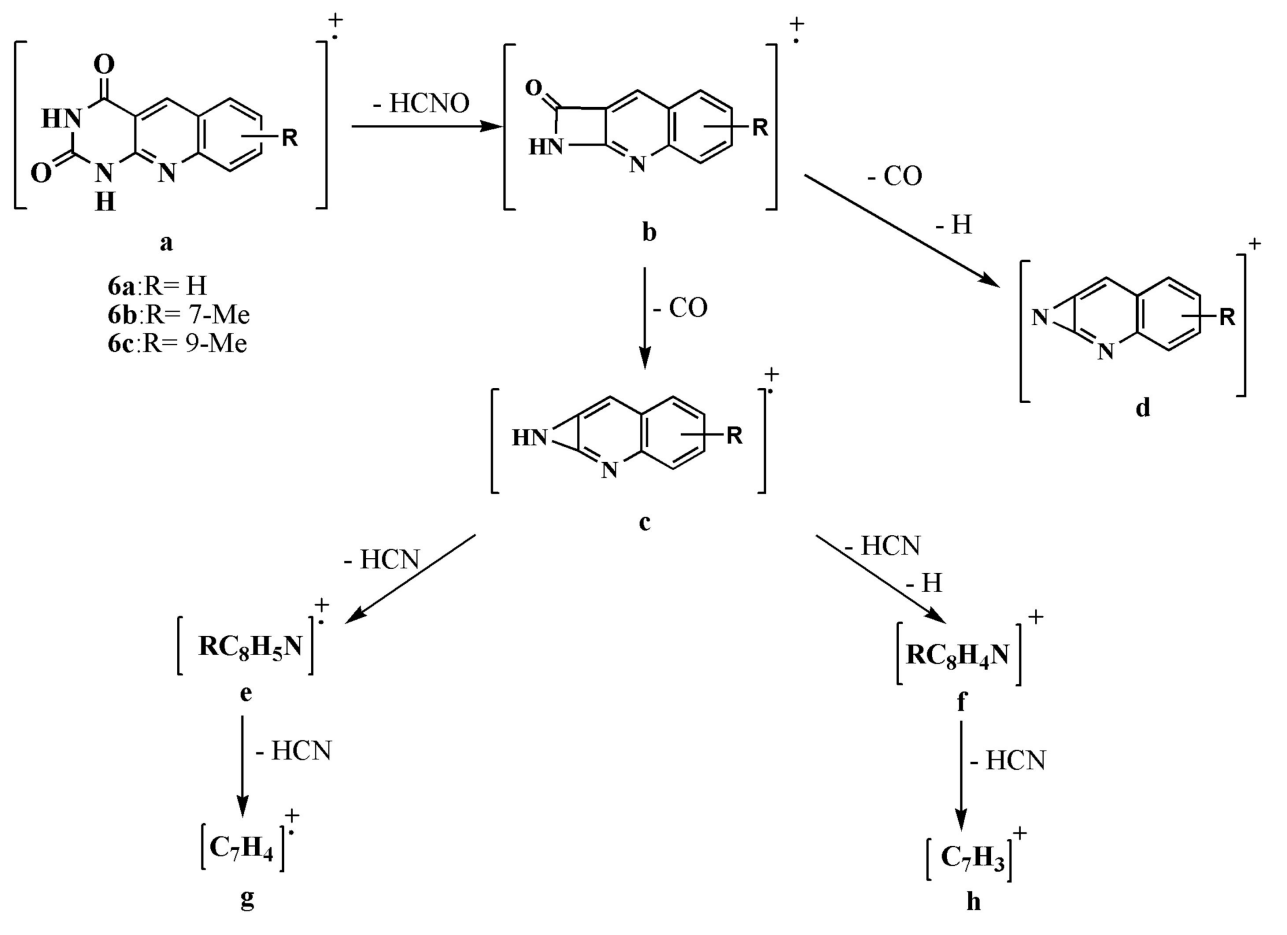
Proposed principal of Electron Ionization Mass Spectrometry (EI-MS) fragmentation routes of compounds (**6a**–**c**).

Based on the reported electron ionization (EI) fragmentation of N (5)-oxides of alloxazine and isoalloxazine derivatives [30] the principal mass spectral fragmentation routes of compounds 2,4-dioxo-5-deazaalloxzines (**6a**–**c**) were proposed as shown in Scheme 2. This includes ions b formed by a retro-Diels-Alder (RDA) reaction by the ejection of an HNCO moiety from the tautomeric pyrimidine ring of (**a**) ions. The next fragmentation involved the elimination of CO or CO and H**^−^** species for the formation of odd-electron and even-electron ions (**c**) and (**d**), respectively. Consecutive ejections of HCN molecules from fragment **c**, with or without elimination of the H˙radical, result in the liberation of fragments **e**, **f**, **g** and **h**, Table 1.

### 2.2. Antiproliferative MTT Assay

The modified 3-(4,5-dimethylthiazol-2-yl)-2,5-diphenyltetrazolium bromide (MTT) assay [31] developed by M. Tim [32] was used to determine the in vitro antiproliferative activities of the test 5-deazaalloxazine compounds against human tumor cell lines, namely, T-cell acute lymphoblastoid leukemia cell line (CCRF-HSB-2) and oral epidermoid carcinoma cell line (KB), using cytosine arabinoside (Ara-C) as a positive control. Another two cells namely: Michigan Cancer Foundation–7 cell line (MCF-7) and cervical cancer cell line (HeLa) were utilized relative to the % viability of 1% DMSO as a negative untreated control.

As can be noticed in Table 2, 2-(cyclohexylamino)-2-deoxo-5-deazaalloxazines (**3d**, **i** and **m**) revealed reasonable antitumor activities; which were the highest among their analogs; against both of CCRF-HSB-2 and KB cell lines of IC_50_ in the range of (6.71–14.91 µM) and (6.35–17.47 µM), respectively, compared to the positive control Ara-C. It is noticed that 2-aminopyrimido[4,5-*b*] quinolin-4(3*H*)-one (**5a**) exhibited high selective antiproliferative activity against KB cell lines over CCRF-HSB-2 cell lines. Its IC_50_ against KB tumor cell lines was 1.46 µM, whereas, its IC_50_ against CCRF-HSB-2 cell lines was >100 µM.

On the other hand, 2-(2-hydroxyethylamino)*-*7-methyl-pyrimido[4,5-*b*] quinolin-4(3*H*)-one (**3h**) revealed a significant antitumor activity; which was the highest among their analogs; against both of MCF-7 and HeLa tumor cell lines of IC_50_ 0.17 µM and 2.00 µM as shown in Table 2, respectively. It is noticed that compounds (**2b**, ***2c***, **3e**, **3f**, **3g**, **4b**, **6a** and **6c**) revealed high selective antiproliferative activity against MCF-7 tumor cell lines over HeLa tumor cell lines. Their IC_50_ against MCF-7 tumor cell lines were 0.22–2.17 µM, whereas, their IC_50_ against HeLa tumor cell lines were >100 µM, respectively. Contrary, compounds (**3c**, **3l**, **6b** and **8**) showed high selectivity against HeLa tumor cell lines over MCF-7 tumor cell lines. Their IC_50_ against HeLa tumor cell lines were 0.57, 0.13, 1.39 and 0.33 µM, whereas, their IC_50_ against MCF-7 tumor cell lines were 8.49, >100, 29.45 and 18.22 µM, respectively.

Interestingly, the IC_50_ of 2-hydroxyethylamino derivative (**3h**) was improved ten times from the IC_50_ of its starting compound 2- methylthio derivatives (**2b**) being of 0.17 µM and 1.79 µM, respectively, against MCF-7 tumor cell line. This suggested that the polar side chain at position 2 enhances the solubility of 5- deazaalloxazine derivatives and could be essential for their antitumor activity. The MTT assay result of Compound **3h** revealed the highest IC_50_ against MCF-7 breast cancer cell line and HeLa cervical cancer cell line (IC_50_ 0.17 µM and 2.00 µM) in comparison to other compounds, which is considered as a new lead for our derivatives and motivated us for further investigation. Nonetheless, this result is less consistent with its binding affinity into c-Kit PTK (∆*G*_b_: −11.07 kcal/mol) and room mean square deviation (RMSD) of 6.13 Å

### 2.3. In Vitro Protein Kinase Assay

**Figure 7:**
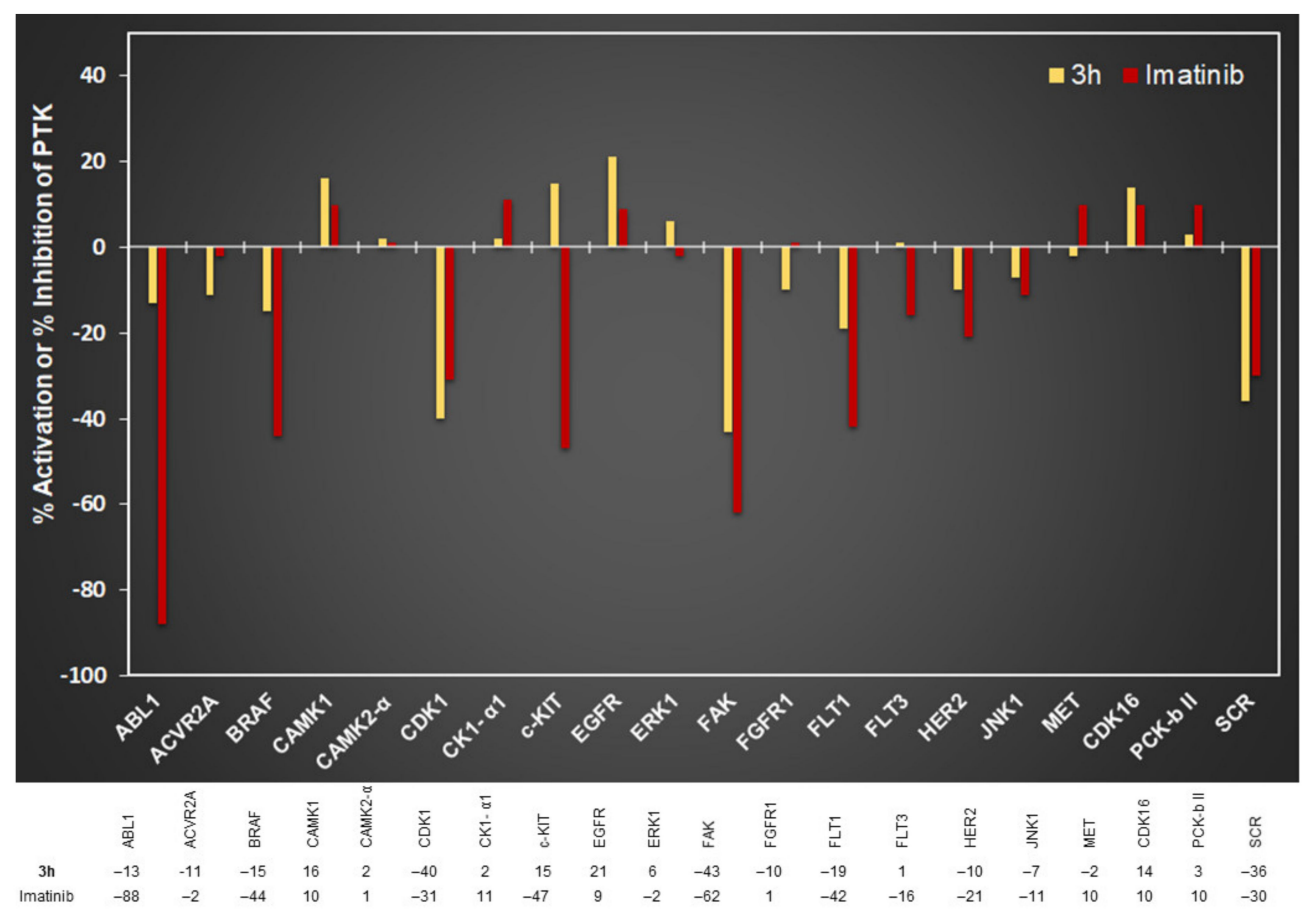
Protein kinase profiling for compound (**3h**) at 10 μM concentration against 20 PKs compared to imatinib.

**Figure 8:**
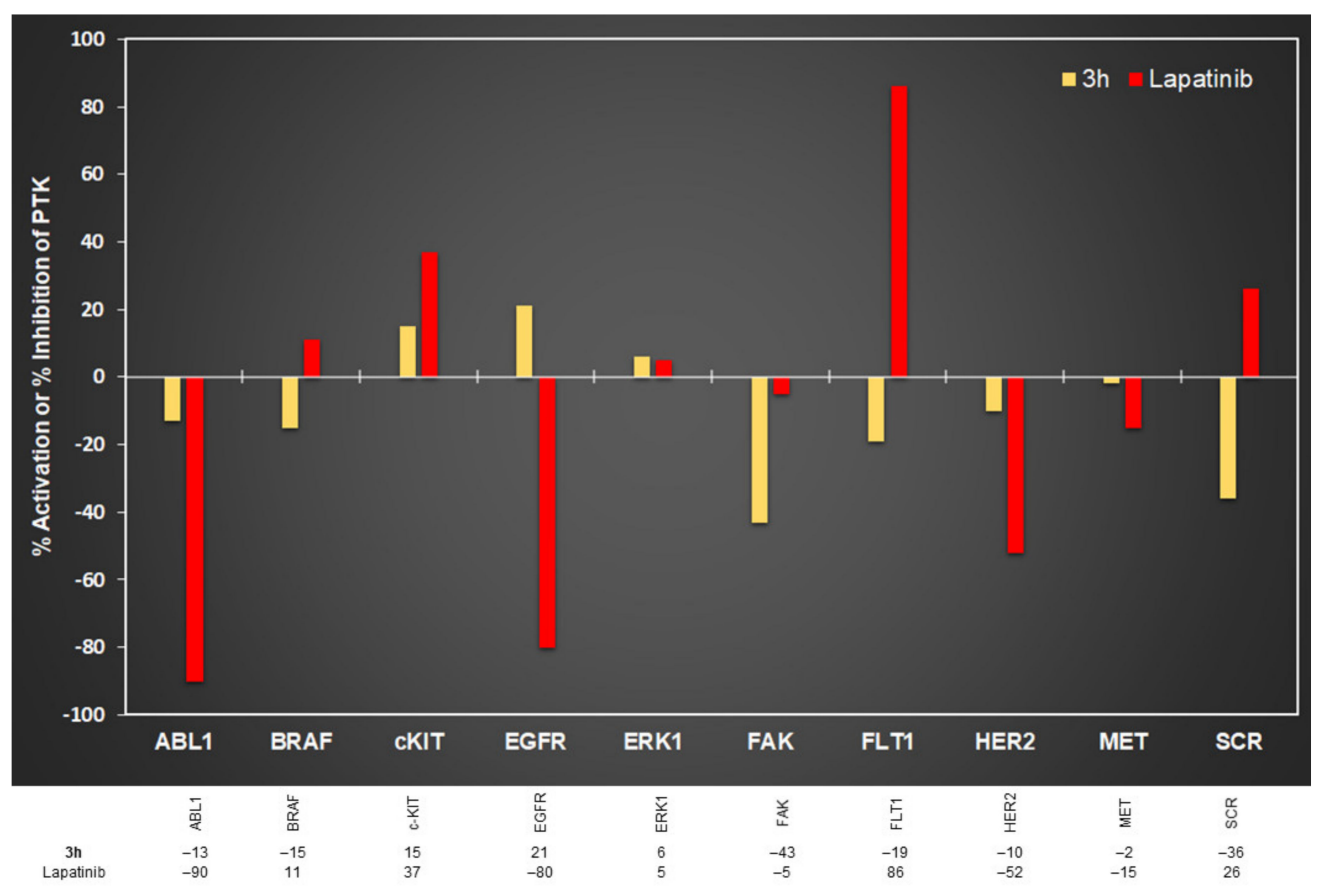
Protein kinase profiling for compound (**3h**) at 10 μM concentration against 10 PKs compared to lapatinib.

Kinase profiling intends to detect the selectivity of a lead compound against a comprehensive panel of kinases by determining its extent of phosphorylation or dephosphorylation. The protein kinase profile of compound (**3h**) against a large group of protein kinases was evaluated using the 33P-γATP radiometric assay method at one concentration (10 μM) in a single measurement by utilizing the methodology of the standardized assay, summarized in the experimental part. The PK profiling assay was performed by KINEXUS Bioinformatics Corporation, Canada. Two reference kinase inhibitors on the phosphotransferase activity were used including Imatinib (BCR-ABL tyrosine kinase inhibitor) and lapatinib (EGFR and HER2 dual selective tyrosine kinase inhibitor). In this study, the results are reported in Figure 7 and Figure 8 as the percentage change in the activation and inhibition in comparison with the control. The negative (−) values indicate the inhibition activity of target by compound, while the positive (+) values indicate the activation activity of the target. The percentages that more than 25% are counted to be a promising result.

Three out of twenty kinases tested by compound (**3h**) exhibited reasonable inhibition scores ranging from −36% to −43% including FAK (−43%), CDK1/Cyclin A1 (−40%) and SCR (−36%) compared to the reference compound Imatinib, which showed significant inhibitory effects with FAK (−62%), CDK1/Cyclin A1 (−31%), SCR (−30%), respectively. Notably, seven out of twenty tested kinases were markedly affected by Imatinib including ABL1, BRAF, CDK1, c-Kit, FAK, FLT1 and SCR kinases with inhibition −88%, −44%, −31%, −47%, −62%, −42% and −30%, respectively.

Another reference compound lapatinib was used as a selective dual kinase inhibitor, which interrupts the HER2 and EGFR pathways. Comparing the 5-deazaalloxazine analog (**3h**) with lapatinib, the compound (**3h**) expressed a moderate inhibition with FAK (−43%), while lapatinib showed slight inhibition (−5%). Moreover, the compound (**3h**) revealed inhibition with BRAF (−15%) and FLT1 (−19%), whereas lapatinib exhibited a slightly to strongly activation with BRAF (11) and FLT1 (86%), as showed in Figure 8. Notably, three out of ten tested kinases were markedly affected by lapatinib, including ABL1, EGFR and HER2 kinases with inhibition −90%, −80%, −52%, respectively. However, lapatinib revealed a moderate to high activation with c-Kit and FLT1 kinases being of 37% to 86% that can boost the deregulation of cellular pathways in cancer patients. Generally, the unregulated activity of the c-Kit kinase is connected to the cancer pathogenesis in human, whereas c-Kit kinase activity is controlled by a tight regulation in the normal cells [33]. Profiling result of compound **3h** revealed slight activation of the c-Kit kinase by 15%, as presented in Figure 8. Interestingly, the binding affinity of this compound has a slight affinity into the binding site of c-Kit PTK (∆*G*_b_ being of −11.07 kcal/mol) and RMSD of 6.13 Å. This docking result could elucidate the activation result of compound **3h** against c-kit kinase.

Overall, this profiling study revealed that the 2-hydroxyethylamino-5-deazaalloxazine analog (3h) has antiproliferative activity by causing multi-target kinases inhibition, including FAK (−43%), CDK1/Cyclin A1 (−40%) and SCR (−36%). This compound (**3h**) has a high solubility in aqueous solutions because of the polar side chain of 2-hydroxyethylamine at position 2, which could be responsible for its fundamental potency (36−43%), which promoted us for further lead optimization studies [34].

### 2.4. Annexin V PI/FITC Apoptosis Assay

According to reported research [35,36] deazaalloxazine analogs expressed a potential capacity of activating the tumor suppressor protein p53 causing a subsequent cell cycle arresting and apoptosis. Annexin V Fluorescein Isothiocyanate/Propidium Iodide (FITC/PI) apoptosis assay was conducted for testing whether different concentrations of compound (**3h**) have apoptosis-inducing activity. The treated and untreated (control) were analyzed with flow cytometry (BC, FC500) after labeling by annexin-V FITC/PI.

**Figure 9:**
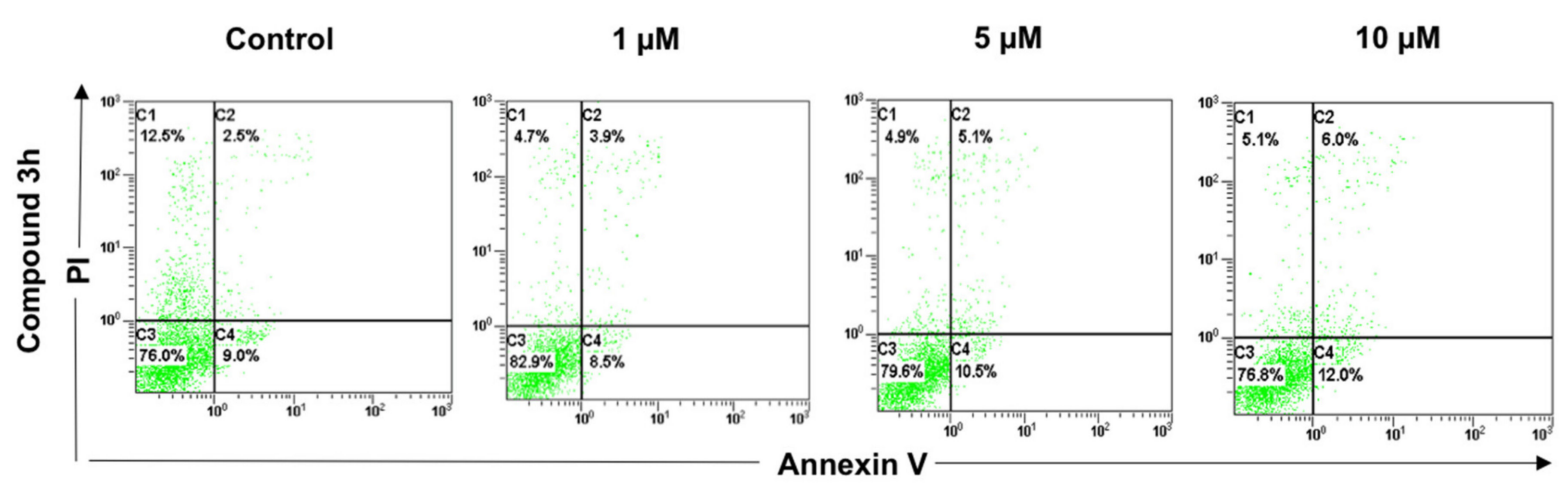
Apoptosis assay for compound **3h** with different concentrations. X-axis: annexin V and Y-axis: PI. live cells (PI−/V−): C3; early apoptotic cells (PI−/V+): C4; late apoptotic cells (PI+/V+): C2; necrotic cells (PI+/V−): C1.

Compound (**3h**) was selected for apoptosis assay regarding its potential inhibitory effect on cell proliferation and multiple kinases, as shown in Table 2, Figure 7 and Figure 8, respectively. Compound (**3h**) induced early and late apoptosis to MCF-7 cells in a dose-dependent pattern. The lowest concentration (1.0 µM) of the compound (**3h**) showed 56% increased late apoptosis compared to the control. Raising the treatment compound (**3h**) dose to 5 then 10 µM resulted in a potential increase in both early and late apoptosis. The highest treatment concentration (10 µM) produced 33% and 140% increase in early and late apoptosis compared to the control, respectively, as showed in Figure 9. Meanwhile, gathering the percentage change in early and late apoptosis between the treatment doses reveals that the most significant shift associated with the second concentration (5 µM). Taken together suggests that the highest overall apoptotic effect of compound (**3h**) was at a dose of (5 µM). On the other hand, MTT assay identified the IC_50_ of compound (**3h**) as 0.17 µM against MCF-7 tumor cell line. MTT assay indicates the percentage of surviving cells compared to control and cannot recognize the mechanism by which the population of the treated cells was decreased [37]. Therefore, different compounds may generally express their antitumor effects by inhibiting different characteristic cellular functions and molecular pathways of cancer without directly inducing apoptosis. Additionally, the PK profiling study of compound (**3h**) demonstrates that this compound has antiproliferative activity by inhibition of FAK (−43%), CDK1/Cyclin A1 (−40%) and SCR (−36%). Herein, it can be suggested that the compound (**3h**) revealed antitumor activity by inhibiting the proliferation and causing apoptosis of the cancer cells.

### 2.5. Structure-Activity Relationship (SAR)

The studies of the structure-activity relationship (SAR) exhibited that the greatest antitumor activities were obtained with the structural features of 5-deazaalloxazin skeleton; the presence of ethanolamine, amino group substituent at C-2 position with unsubstituted quinoline nucleus or substituted by 7-Me revealed good antitumor activity as shown for compounds **3h** and **5a**. Additionally, unsubstituted quinoline scaffold or substituted by 7-OMe group, 7-Me group or 9-Me group with thiomethyl, ethanolamine, butylamine, propylamine, morpholine or oxo group at C-2 position show high selective antiproliferative activity against MCF-7 tumor cell lines as shown for compounds **2b,c**, **3e**–**g**, **4b**, **6a** and **6c**. Moreover, unsubstituted quinoline scaffold or substituted by a 7-Me group or 9-Me group with cyclohexyl, propylamino, oxo group or without substitution at C-2 position show high selective antiproliferative activity against HeLa tumor cell lines as shown for compounds **2c**, **3i**, **6b** and **8**.

### 2.6. Molecular Docking Study

The AutoDock binding affinities of various 5-deazaalloxizine and 5-deazaflavin analogs for their potential protein tyrosine inhibitory activities have been investigated [8]. Herein, the molecular docking study was conducted using AutoDock 4.2 [38] by docking of synthesized derivatives into the c-Kit receptor PTK: platelet-derived growth factor receptor (PDGFR, c-kit; PDB code: 1t46) and co-crystallized with the c-kit PTK inhibitor (STI571).

The selection criteria used for molecular docking are listed as (1) full structure of the kinase domain of the retrieved protein from PDB structures, (2) high quality of the protein structure in this study the quality of chai A used is 83%, (3) minimum resolution to ensure high quantity of the gathered data from the used kinase (1.60 Å for PDB: 1t46), (4) Using a kinase model co-crystallized with inhibitor (Imatinib, Gleevec or STI-571) to be used for validation of the accuracy of the docking algorithm of the software (AutoDock 4.2) and (5) to ensure removal of the native co-crystallized ligand, water molecules and cofactor (if available) before docking. Docking accuracy will be verified if the bound inhibitor (STI) is docked within RMSD ≤ 2.0 Å from the natively co-crystallized pose. [39]. In addition, the docked compounds should reveal binding free energies (Δ*G*_b_) comparable to the native ligand and display a large number of hydrogen bonds with the most relevant amino acids (E640, T670, C673 and D810) involved in the inhibitor interaction into c-kit PTK (1t46).

#### 2.6.1. AutoDock Validation

**Figure 10:**
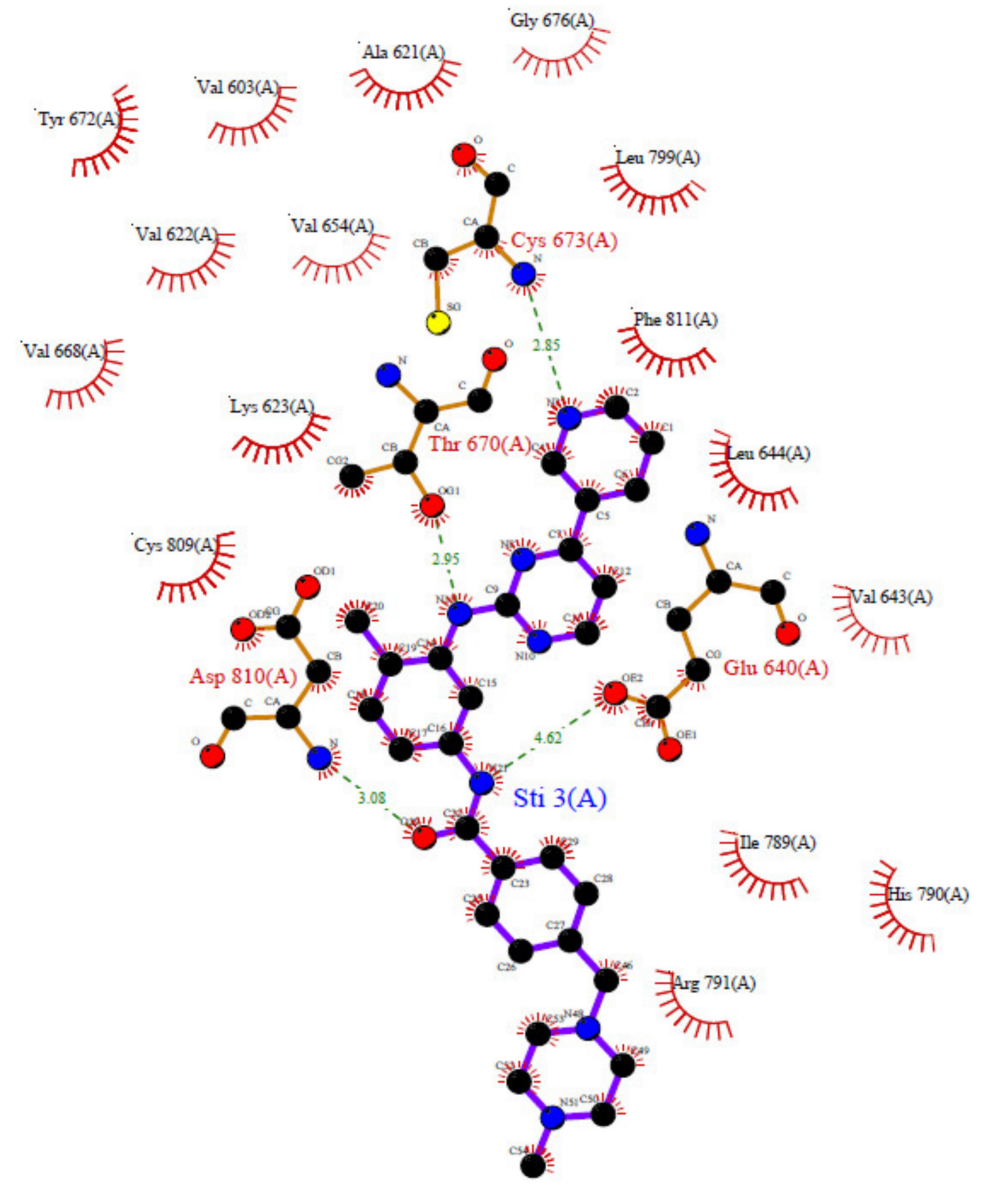
The LIGPLOT of interactions involving the co-crystallized ligand (Imatinib, Gleevec or STI) revealing the most relevant amino acids (E640, T670, C673 and D810) involved in the inhibitor binding into c-kit kinase (PDB: 1t46), through PDBsum database.

The docking accuracy of AutoDock had been validated by docking the native cocrystallized ligand (STI) into its binding site of PTK (PDB: 1t46) to examine the similarity between the docked conformation and the original ligand [39]. The original ligand (Imatinib, Gleevec or STI-571) showed four hydrogen bonds with the essential amino acids of PTK: E640, T670, C673 and D810 (Figure 10). The docked STI ligand appeared overlapped on the original STI ligand within RMSD being of 0.17 Å. Therefore, AutoDock validation was verified to allow further docking of the designed analogs [40]. These results similarly emulate the experimental results, revealing a successful docking protocol and confirming the accuracy of the GOLD docking program for further docking of the designed hits.

#### 2.6.2. AutoDock Binding Affinities of the Synthesized Compounds into C-Kit Tyrosine Kinase

On applying the flexible docking of the 5-deazaalloxazine derivatives into the binding site of PTK (PDB: 1t46), the test compounds revealed reasonable binding free energy (∆*G*_b_) compared to the natively bound STI-571 ligand (Table 3). The promising poses were exhibited by compounds **2b**, **2e**, **3d**, **3e**, **3i**, **3m** and **4a**–**d**, that showed binding free energies (∆*G*_b_) in the range of −13.71 to −12.33 kcal/mol within RMSD range of 1.31–7.88 Å and interacting by 1–3 hydrogen bonds.

**Figure 11:**
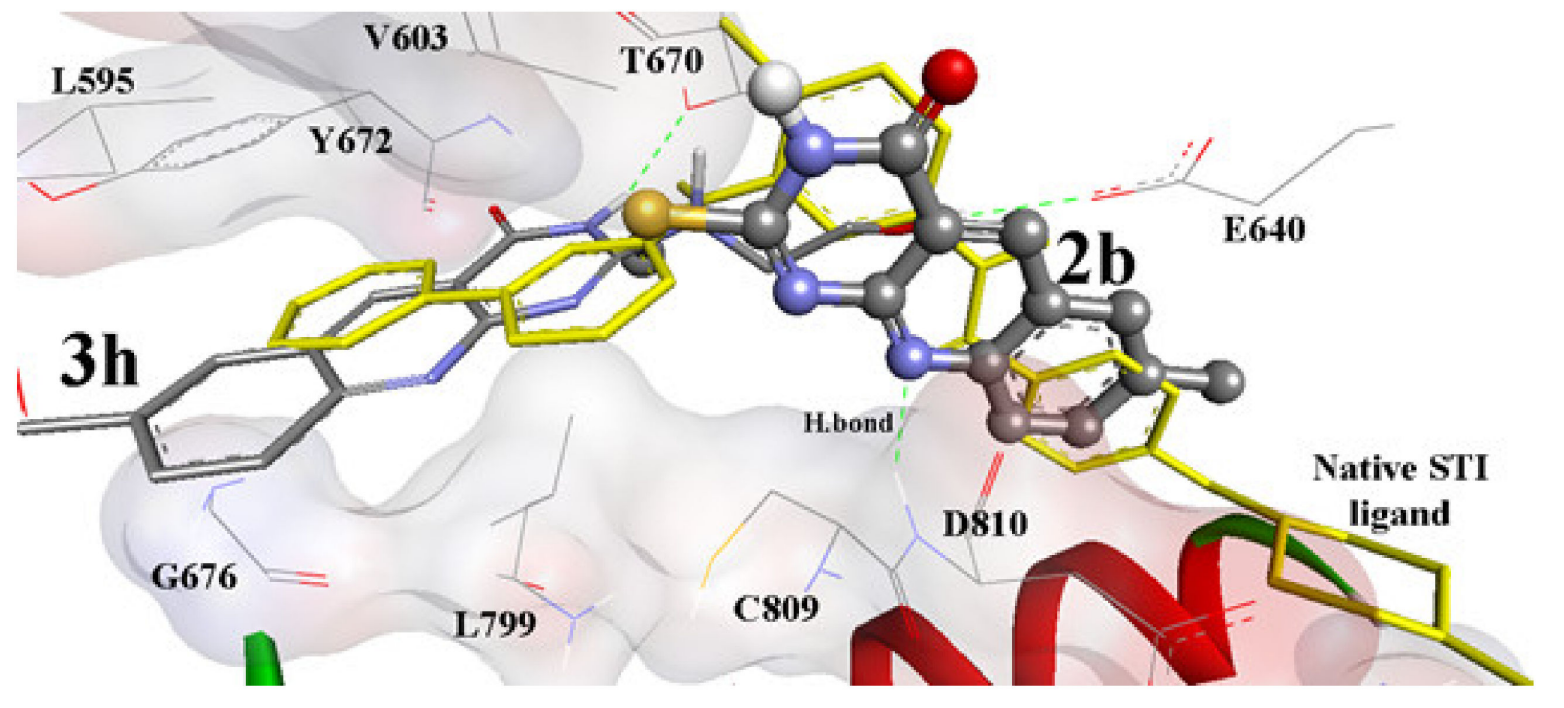
The comparative binding affinities of compound **2b** (colored by element, ball and stick) and **3h** (colored by element, sticks) into the binding site of protein Tyrosine kinase (PTK) (1t46). They showed one and two hydrogen bonds with D810, E640 and T670 amino acids, respectively. The STI ligand is shown as yellow sticks. The hydrogen bonds are drawn as green dashed lines.

The significant PTK inhibition of compound **2b** with IC_50_ against CCRF-HSB-2 and KB tumor cell lines of 23.66 and 26.73 µM, respectively and compound ***3h*** with IC_50_% against MCF-7 and HeLa tumor cells of 0.17 and 2.0 µM, respectively were correlated with their binding affinities. Where they were docked into PTK (PDB: 1t46) as depicted in Figure 11 compared to the bound native ligand. Compound **2b** revealed higher binding affinity with Δ*G*_b_: −13.04 kcal/mol and one hydrogen bond within RMSD of 1.31 Å. Whereas, compound 3h exhibited less binding affinity into with ∆*G*_b_ of −11.07 kcal/mol within RMSD of 6.13Å. 3h was bound through two hydrogen bonds. This results indicated that compound **2b** is predicted to be a significant lead compound for PTK inhibition by its appropriate fitting into the binding site.

**Figure 12:**
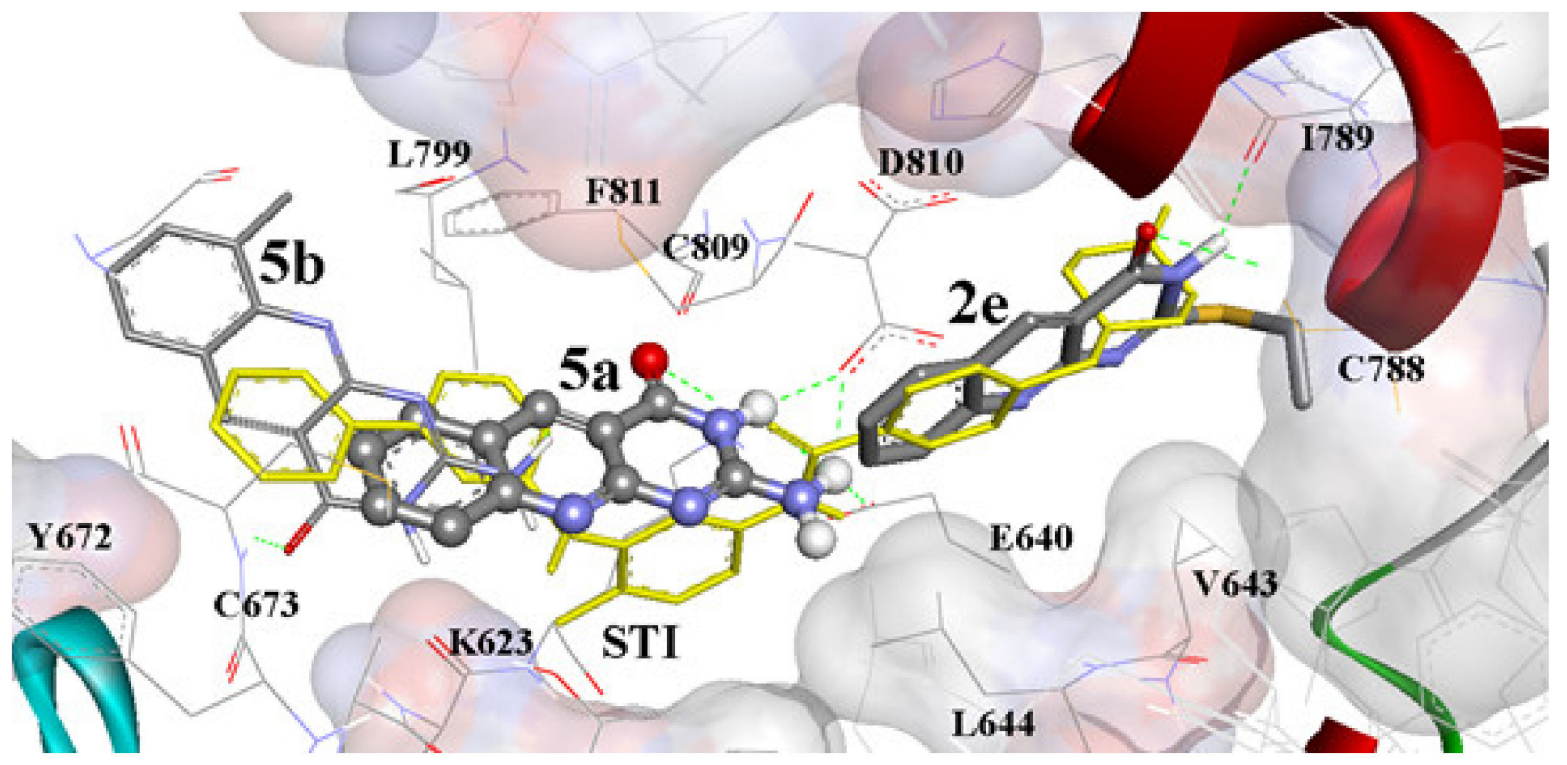
Differential docking affinities of compounds **2e** (bold sticks), **5a** (ball and stick) and **5b** (thin sticks) involving flexible docking into c-Kit PTK (1t46). Where compounds **2e** and **5a** seem docked exactly superimposed on the native STI ligand (yellow sticks) revealing two and four hydrogen bonds, whereas, **5b** has deviated away with only one hydrogen bond. The c-Kit PTK is shown as a solid backbone ribbon and the hydrogen bonds as green dotted lines.

On the other hand, the flexible docking of compounds ***2e***, **5a** and **5b**, exhibited comparable binding affinities with ∆*G*_b_ in the range of –12.60 to –10.45 kcal/mol and 1–4 hydrogen bonds, within RMSD in the range of 2.46–8.04 Å, as shown in Figure 12.

**Figure 13:**
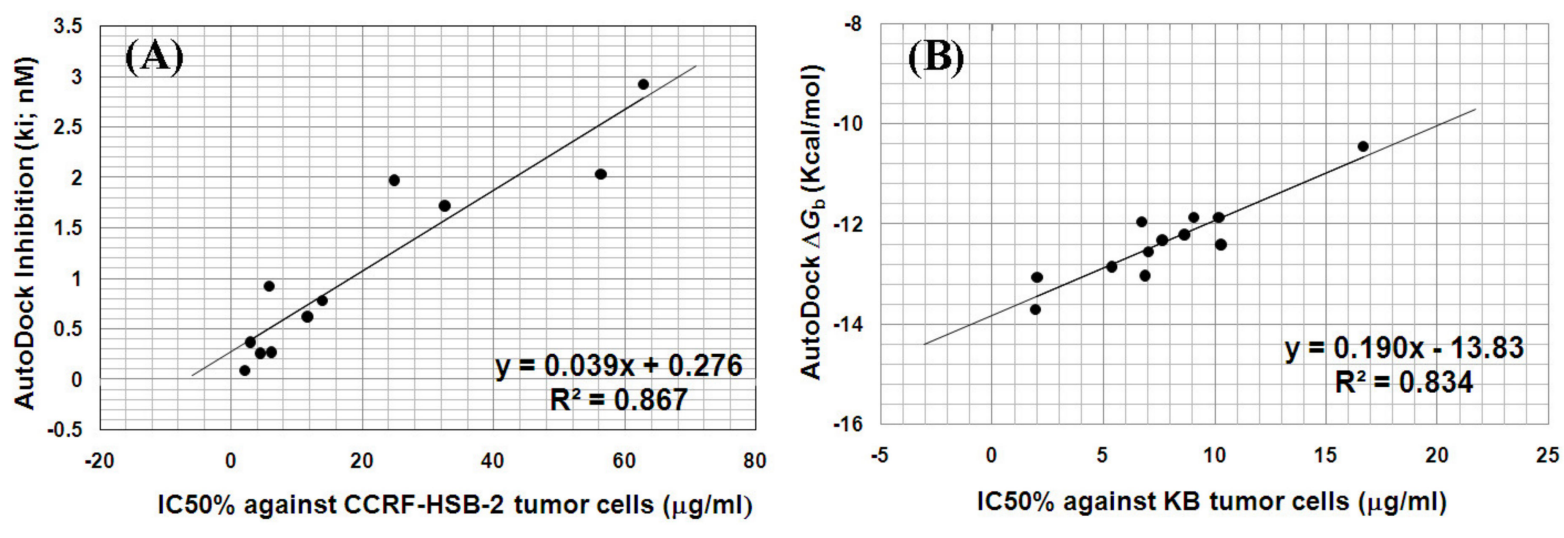
(**A**) Correlation between the IC_50_ (µg/mL) against CCRF-HSB-2 tumor cell lines and AutoDock inhibition (Ki) for compounds **2b**, **3d**, **3i**, **3j**, **3m**, **3k**, **4a**, **4c**, **4d**, **4e** and **5a**. (**B**) Correlation between the IC_50_ (µg/mL) against KB tumor cell lines and AutoDock binding free energy (∆*G*_b_) for compounds **2b**, **3a**, **3d**, **3i**, **3j**, **3m**, **4a**, **4c**, **4d**, **4e**, **4g** and **5b**.

The correlation between the antiproliferative activities IC_50_ (µg/mL) of compounds (**2b**, **3d**, **3i**, **3j**, **3m**, **3k**, **4a**, **4c**, **4d**, **4e** and **5a**) against human CCRF-HSB-2 cell line and the AutoDock inhibition constants (Ki) was sensible with a correlation coefficient (R^2^) of 0.867 as represented in Figure 13A. whereas, the correlation between IC_50_ (µg/mL) of compounds **2b**, **3a**, **3d**, **3i**, **3j**, **3m**, **4a**, **4c**, **4d**, **4e**, **4g** and **5b** against human KB cell line and AutoDock binding free energies (Δ*G*_b_) showed a reasonable correlation coefficient (R^2^) of 0.834 as represented in Figure 13B.

To a lower extent, the correlation coefficient (R^2^) between Autodock inhibition (Ki) and IC_50_ (µM) against HeLa and MCF-7 tumor cell lines were 0.719 and 0.684, respectively.

## 3. Experimental

### 3.1. Synthesis and Structural Elucidation of 5-Deazaalloxazine Derivatives

Using an electrothermal capillary melting point apparatus, MPs were measured as uncorrected. IR spectra were recorded using of infrared spectrophotometer of Perkin Elmer model 137 with KBr disk. A Varian Mercury VX-300 MHz spectrophotometer was used to analyze the ^1^H-NMR spectra and the chemical shift values were measured in in δ values (ppm). Tetramethylsilane (TMS) was used as an internal reference. The coupling constants are expressed in Hz. Both protons of NH and OH protons were measured as exchangeable with D_2_O. Gas chromatograph-mass spectrometer-single quadrupole (GCMC-QP)1000 EX SHIMADZU Gas Chromatography MS spectrometer was used to record the mass spectra. The elemental analyses were conducted by using an Automatic (Carbon; Hydroge; Nitrogen CHN) analyzer, Vario E1III, Elementary-Germany at the Microanalytical Center, Faculty of Science Cairo University, Egypt. All reagents in this study were used in a commercial quality and were not subjected for further purification. Drying of the organic solvents was performed by using suitable drying agents and stored over appropriate molecular sieves. Thin-layer chromatography (TLC) using pre-coated glass plates silica gel plates of 60F254-Merck was used for monitoring the progress of reactions and our fluorescent products were visualized by a UV lamp.

#### 3.1.1. General Procedure for the Preparation of *2-*Alkylthiopyrimido[4,5-*b*] Quinoline-4(3*H*)-Ones {2-Deoxo-2-Alkylthio-5-Deazaalloxazines} (**2a**–**e**)

A mixture of the appropriate 6-(*N*-arylamino)-2-alkylthiopyrimidin-4(3*H*)-one (1, 0.01 mole) and POCl_3_ (7.7 g, 0.05 mole) in (10 mL) of anhydrous DMF was heated with stirring at 90 °C for 1–2 h. Then, the reaction mixture was poured onto ice/water mixture and neutralized at pH 7 by aqueous ammonia. The separated yellow crystals were filtered off, then washed with water, dried and recrystallized by using DMF-H2O to produce the products as pale-yellow needles in 72–86% yields.

*2-(Methylthio)pyrimido[4,5-b]quinoline-4(3H)-one* (**2a**)*:* Yield (1.75 g, 72%, DMF-H_2_O); m.p. 292–294 °C, as reported [8].

*7-Methyl-2-(methylthio)pyrimido[4,5-b]quinoline-4(3H)-one* (**2b**): Yield (2.0 g, 78%, DMF-H_2_O); m.p. 244–246 °C; UV (EtOH): λ_max_nm (logε/dm^3^ mol^−1^cm^−1^): 254 (4.03), 278sh (4.06), 285 (4.08), 366 (3.55); IR (ν_cm_^−1^): 3421 (NH), 1672 (C=O); *^1^*H-NMR [(CD_3_)_2_SO-d_6_]: δ 2.51 (3H, s, 7-Me), 2.65 (3H, s, 2-SMe), 7.73 (1H, d, *J_8,9_* = 6.0 Hz, 9-H), 7.90 (1H, d, *J_8,9_* = 6.0 Hz, 8-H), 7.92 (1H, s, 6-H), 9.07 (1 H, s, 5-H), 12.61 (1H, br s, NH, D_2_O exchangeable); % CHN analysis: Anal. Calcd for C_13_H_11_N_3_OS: C, 60.68; H, 4.31; N, 16.33. Found: C, 60.49; H*,* 4.22; N, 16.69; MSEI, *m*/*z* (%): 257 (M^+^, 95%), 242 (6%), 226 (100%), 155 (82%).

*7-Methoxy-2-(methylthio)pyrimido[4,5-b]quinoline-4(3H)-one* (**2c**)*:* Yield (2.10 g, 77%, DMF-H_2_O); m.p. 222–224 °C; UV (EtOH): λ_max_nm (logε/dm^3^ mol^–1^cm^–1^): 230 (4.36), 266sh (4.39), 285 (4.40), 401 (3.51); IR (ν_cm_^−1^): 3399 (NH), 1677 (C=O); ^1^H-NMR [(CD_3_)_2_SO-d_6_]: δ 2.56 (3H, s, 2-SMe), 3.91 (3H, s, 7-OMe), 7.57(1H, d, *J_8,9_* = 9.0 Hz, 8-H), 7.59 (1H, s, 6-H), 7.93 (1H, d, *J_8,9_* = 9.0 Hz, 9-H), 9.01(1H, s, 5-H), 12.68 (1H, br s, NH, D_2_O exchangeable); % CHN analysis: Anal. Calcd for C_13_H_11_N_3_O_2_S.0.5 DMF: C, 56.20; H, 4.72; N, 15.82. Found: C, 55.98; H, 4.52; N, 15.55; MSEI, *m*/*z* (%): 273 (M^+^, 40%), 256 (11%), 249 (36%), 55 (100%).

*9-Methyl-2-(methylthio)pyrimido[4,5-b]quinoline-4(3H)-one* (**2d**): Yield (2.20 g, 86%, DMF-H_2_O); m.p. 200–202 °C; UV (EtOH): λ_max_nm (logε/dm^3^ mol^–1^cm^–1^): 244 (4.35), 279sh (4.49), 287 (4.55), 367 (3.81); IR (ν_cm_^−1^): 3412 (NH), 1681 (C=O); *^1^*H-NMR [CDCl_3_-d_6_]: δ 2.54 (3H, s, 9-Me), 2.85 (3H, s, 2-SMe), 7.46–7.48 (1H, m, 7-H), 7.71–7.73 (1H, m, 8-H), 7.83–7.85 (1H, m, 6-H), 9.14 (1H, s, 5-H), 11.78 (1H, br s, NH, D_2_O exchangeable); % CHN analysis: Anal. Calcd for C_13_H_11_N_3_OS: C, 60.68; H, 4.31; N, 16.33. Found: C, 60.35; H, 4.63; N, 16.01; MSEI, *m*/*z* (%): 257 (M^+^, 65%), 208 (100%), 154 (81%).

*2-(Ethylthio)pyrimido[4,5-b] quinoline-4(3H)-one* (***2e***)*:* Yield (1.90 g, 74%, DMF-H_2_O); m.p. 240–242 °C; UV (EtOH): λ_max_nm (logε/dm^3^ mol^–1^cm^–1^): 243 (4.35), 269sh (4.40), 284 (4.53), 362 (3.79); IR (ν_cm_^−1^): 3416 (NH), 1680 (C=O); ^1^H-NMR [CDCl_3_-d_6_]: δ 1.52 (3H, t, *J* = 6.0 Hz, 2-SCH_2_CH_3_), 3.50 (2H, q, *J* = 6.0 Hz, 2-SCH_2_CH_3_), 7.60 (1H, t, *J_6,7_* = *J_7,8_* = 7.2 Hz, 7-H), 7.91 (1H, t, *J_7,8_* = *J_8,9_* = 7.2 Hz, 8-H), 8.02 (1H, d, *J_8,9_* = 7.2 Hz, 9-H), 8.26 (1H, d, *J_6,7_* = 7.2 Hz, 6-H), 9.18 (1 H, s, 5-H), 9.43 (1H, br s, NH, D_2_O exchangeable); % CHN analysis: Anal. Calcd for C_13_H_11_N_3_OS: C, 60.68; H, 4.31; N, 16.33. Found: C, 61.02; H, 4.56; N, 16.61; MSEI, *m*/*z* (%): 257 (M^+^, 34%), 229 (34%), 170 (32%), 141 (94%) and 114 (100%).

#### 3.1.2. General Procedure for the Preparation of 2-(Substituted alkyl amino)-pyrimido[4,5-*b*] quinoline-4(3*H*)-ones {2-(substituted alkyl amino)-2-deoxo-5-deazaalloxazines} (**3a**–**m**)

A 0.01 mole of 2-(methylthio)pyrimido[4,5-*b*]quinolin-4(3*H*)-one derivatives (2) was mixed and refluxed with stirring with 0.1 moles of the appropriate amine for 7–9 h. The clear solution of the yellow product was kept overnight in the refrigerator to allow precipitation of yellow crystals which were collected by filtration to get the first crop. The excess amine was removed from the filtrate by concentrating in vacuo, then the residue was treated with ether then water to get the second crop free from amine. The collected solids were dried and recrystallized from DMF/H_2_O mixture to afford pure products with 60–88% as yellow needles.

*2-(2-Hydroxyethylamino)pyrimido[4,5-b]quinoline-4(3H)-one* (***3a***)*:* Yield (2.1 g, 82%, DMF-H_2_O); m.p. 273–275 °C; UV (EtOH): λ_max_nm (logε/dm^3^ mol^−1^cm^−1^): 226 (4.31), 261sh (4.25), 277 (4.34), 372 (3.49); IR (ν_cm_^−1^): 3389 (OH), 3247 (NH), 1694 (C=O); ^1^H-NMR [(CD_3_)_2_SO-d_6_]: δ 3.58–3.60 (2H, m, NCH_2_), 3.67–3.69 (2H, m, OCH_2_), 5.01 (1H, br s, OH, D_2_O exchangeable), 6.88 (1H, br s, NH, D_2_O exchangeable), 7.43 (1H, t, *J_6,7_* = *J_7,8_* = 9.0 Hz, 7-H), 7.78 (1H, t, *J_7,8_* = *J_8,9_* = 9.0 Hz, 8-H), 7.82 (1H, d, *J_8,9_* = 9.0 Hz, 9-H), 8.06 (1H, d, *J_6,7_* = 9.0 Hz, 6-H), 8.92 (1H, s, 5-H), 11.10 (1H, br s, NH, D_2_O exchangeable); % CHN analysis: Anal. Calcd for C_13_H_12_N_4_O_2_: C, 60.93; H, 4.72; N, 21.86. Found: C, 61.23; H, 5.01; N, 21.54.

*2-(Butylamino)pyrimido[4,5-b]quinoline-4(3H)-one* (**3b**): Yield (1.60 g, 60%, DMF-H_2_O); m.p. 153–155 °C; UV (EtOH): λ_max_nm (logε/dm^3^ mol^−1^cm^−1^): 243 (4.36), 263 (4.42), 278sh (4.40), 370 (3.71); IR (ν_cm_^−1^): 3431, 3256 (NH), 1691 (C=O); ^1^H-NMR [(CD_3_)_2_SO-d_6_]*:* δ 0.91 (3H, br s, 2-NH(CH_2_)_3_CH_3_), 1.36–1.38 (2H, m, 2-NH(CH_2_)_2_CH_2_-), 1.52–1.54 (2H, m, 2-NH-CH_2_CH_2_-), 3.16–3.18 (2H, m, 2-NHCH_2_-), 6.90 (1H, br s, NH, D_2_O exchangeable), 7.44–7.46 (1H, m, 7-H), 7.59–7.61 (1H, m, 8-H), 7.78–7.80 (1H, m, 9-H), 8.04–8.06 (1H, m, 6-H), 8.90 (1H, s, 5-H), 10.65 (1H, br s, NH, D_2_O exchangeable); % CHN analysis: Anal. Calcd for C_15_H_16_N_4_O.0.3 H_2_O: C, 65.82; H, 6.11; N, 20.47. Found: C, 65.98; H, 5.97; N, 20.26.

*2-(Propylamino)pyrimido[4,5-b]quinoline-4(3H)-one* (**3c**): Yield (1.90 g, 75%, DMF-H_2_O); m.p. 254–256 °C; UV (EtOH): λ_max_nm (logε/dm^3^ mol^−1^cm^−1^): 243 (4.0), 261sh (4.12), 278 (4.26), 372 (3.41); IR (ν_cm_^−1^): 3448, 3232 (NH), 1634 (C=O); ^1^H-NMR [(CD_3_)_2_SO-d_6_]: δ 0.90–0.95 (3H, m, 2-NH(CH_2_)_2_CH_3_), 1.52–1.60 (2H, m, 2-NHCH_2_CH_2_-), 3.35–3.37 (2H, m, 2-NHCH_2−_), 6.68 (1H, br s, NH, D_2_O exchangeable), 7.43 (1H, t, *J_6,7_* = *J_7,8_* = 8.4 Hz, 7-H), 7.76 (1H, t, *J_7,8_* = *J_8,9_* = 8.4 Hz, 8-H), 7.82 (1H, d, *J_8,9_* = 8.4 Hz, 9-H), 8.04 (1H, d, *J_6,7_* = 8.4 Hz, 6-H), 8.91 (1H, s, 5-H), 11.02 (1H, br s, NH, D_2_O exchangeable); % CHN analysis: Anal. Calcd for C_14_H_14_N_4_O: C, 66.13; H, 5.55; N, 22.03. Found: C, 65.98; H, 5.34; N, 22.35; MSEI, *m*/*z* (%): 254 (M^+^, 25%), 225 (76%), 212 (100%).

*2-(Cyclohexylamino)pyrimido[4,5-b]quinoline-4(3H)-one* (**3d**)*:* Yield (2 g, 68%, DMF-H_2_O); m.p. 229–231 °C; UV (EtOH): λ_max_nm (logε/dm^3^ mol^−1^cm^−1^): 242 (4.26), 262sh (4.37), 279 (4.49), 370 (3.71); IR (ν_cm_^−1^): 3425, 3250 (NH), 1688 (C=O); ^1^H-NMR [(CD_3_)_2_SO-d_6_]: δ 1.29–1.35 (2H, m, 4’-CH_2_), 1.52–1.71 (4H, m, 3’ and 5’-CH_2_), 1.95–2.20 (4H, m, 2′-CH_2_ and 6′-CH_2_), 2.97–2.99 (1H, m, 1’-CH-), 3.93 (1H, br s, NH, D_2_O exchangeable), 7.40–7.42 (1H, m, 7-H), 7.80–7.82 (2H, m, 8 and 9-H), 8.04–8.06 (1H, m, 6-H), 8.91 (1H, s, 5-H), 10.67 (1H, br s, NH, D_2_O exchangeable); % CHN analysis: Anal. Calcd for C_17_H_18_N_4_O: C, 69.37; H, 6.16; N, 19.03. Found: C, 68.01; H, 5.89; N, 18.85; MSEI, *m*/*z* (%): 294 (M^+^, 12%), 212 (89%), 57 (100%).

*2-(2-Hydroxyethylamino)-7-(methoxy)pyrimido[4,5-b]quinoline-4(3H)-one* (**3e**): Yield (2.4 g, 84%, DMF-H_2_O); m.p. 190–192 °C; UV (EtOH): λ_max_nm (logε/dm^3^ mol^–1^cm^–1^): 224 (4.08), 277 (4.16), 327 (3.69), 401 (3.23); IR (ν_cm_^−1^): 3301 (OH), 3218 (NH), 1693 (C=O); ^1^H-NMR [(CD_3_)_2_SO-d_6_]: δ 3.68–3.70 (2H, m, NCH_2_), *3.72 (3H, s, 7-OMe), 3.85–3.87 (2H, m, OCH_2_), 4.72 (1H, br s, OH, D_2_O exchangeable), 6.52 (1H, br* s, NH, D_2_O exchangeable), 7.05–7.07 (1H, m, 9-H), 7.63 (1H, s, 6-H), 7.80–7.82 (1H, m, 8-H), 8.79 (1H, s, 5-H), 9.43 (1H, br s, NH, D_2_O exchangeable); % CHN analysis: Anal. Calcd for C_14_H_14_N_4_O_3_: C, 58.73; H, 4.93; N, 19.57. Found: C, 58.21; H, 5.29; N, 19.25; MSEI, *m*/*z* (%): 287 (M^+^+1, 17%), 270 (42%), 255 (17%), 108 (100%).

*2-Butylamino-7-(methoxy)pyrimido[4,5-b]quinoline-4(3H)-one* (**3f**): Yield (2.2 g, 74%, DMF-H_2_O); m.p. 242–243 °C; UV (EtOH): λ_max_nm (logε/dm^3^ mol^−1^cm^−1^): 230 (4.37), 269 (4.41), 370 (3.67), 394 (3.66); IR (ν_cm_^−1^): 3398, 3269 (NH), 1694 (C=O); ^1^H-NMR [(CD_3_)_2_SO-d_6_]: δ 0.91 (3H, t, *J* = 7.2 Hz, 2-NH(CH_2_)_3_CH_3_), 1.37–1.39 (2H, m, 2-NH(CH_2_)_2_CH_2_-), 1.53–1.55 (2H, m, 2-NH-CH_2_CH_2_-), 3.75–3.77 (2H, m, 2-NHCH_2_-), 3.87 (3H, s, 7-OMe), 6.89 (1H, br s, NH, D_2_O exchangeable), 7.46–7.49 (2H, m, 6 and 9-H), 7.73–7.75 (1H, m, 8-H), 8.83 (1H, s, 5-H), 10.98 (1H, br s, NH, D_2_O exchangeable); % CHN analysis: Anal. Calcd for C_16_H_18_N_4_O_2_: C, 64.41; H, 6.08; N, 18.78. Found: C, 64.13; H, 5.82; N, 19.04; MSEI, *m*/*z* (%): 298 (M^+^, 55%), 282 (8%), 270 (26%), 255 (100%), 242 (94%), 227 (59%).

*7-Methoxy-2-(propylamino)pyrimido[4,5-b]quinoline-4(3H)-one* (**3g**): Yield (2.5 g, 88%, DMF-H_2_O); m.p. 268–270 °C; UV (EtOH): λ_max_nm (logε/dm^3^ mol^−1^cm^−1^): 229 (4.36), 267 (4.45), 370 (3.60), 396 (3.69); IR (ν_cm_^−1^): 3391, 3241 (NH), 1694 (C=O); ^1^H-NMR [(CD_3_)_2_SO-d_6_]: 0.91 (3H, t, *J* = 6.3 Hz, 2-NH(CH_2_)_2_CH_3_), 1.58 (2H, hexet, *J* = 6.3 Hz, 2-NHCH_2_CH_2_-), 3.73–3.76 (2H, m, 2-NHCH_2_-), 3.86 (3H, s, 7-OMe), 6.52 (1H, br s, NH, D_2_O exchangeable), 7.43 (1H, d, *J_8,9_* = 9 Hz, 9-H), 7.52 (1H, s, 6-H), 7.74 (1H, d, *J_8,9_* = 9 Hz, 8-H), 8.81 (1H, s, 5-H), 10.93 (1H, br s, NH, D_2_O exchangeable); % CHN analysis: Anal. Calcd for C_15_H_16_N_4_O_2_: C, 63.37; H, 5.67; N, 19.71. Found: C, 63.14; H, 5.35; N, 19.94; MSEI, *m*/*z* (%): 284 (M^+^, 53%), 269 (14%), 255 (83%), 242 (100%), 227 (48%).

*2-(2-Hydroxyethylamino)-7-(methyl)pyrimido[4,5-b]quinoline-4(3H)-one* (**3h**): Yield (2.1 g, 78%, DMF-H_2_O); m.p. 200–210 °C; UV (EtOH): λ_max_nm (logε/dm^3^ mol^−1^cm^−1^): 228 (4.32), 265 (4.44), 281sh (4.35), 360 (3.65); IR (ν_cm_^−1^): 3385 (OH), 3243 (NH), 1696 (C=O); ^1^H-NMR [(CD_3_)_2_SO-d_6_]: δ 2.65 (3H, s, 7-Me), 3.49–3.51 (2H, m, NCH_2_), 3.58–3.60 (2H, m, OCH_2_), 4.95 (1H, br s, OH, D_2_O exchangeable), 6.75 (1H, br s, NH, D_2_O exchangeable), 7.32–7.34 (1H, m, 9-H), 7.62 (1H, s, 6-H), 7.89–7.81 (1H, m, 8-H), 8.89 (1H, s, 5-H), 11.12 (1H, br s, NH, D_2_O exchangeable); % CHN analysis: Anal. Calcd for C_14_H_14_N_4_O_2_: C, 62.21; H, 5.22; N, 20.73. Found: C, 62.53; H, 5.54; N, 20.52.

*2-Cyclohexylamino-7-(methyl)pyrimido[4,5-b]quinoline-4(3H)-one* (**3i**): Yield (2.0 g, 65%, DMF-H_2_O); m.p. 220–225 °C; UV (EtOH): λ_max_nm (logε/dm^3^ mol^−1^cm^−1^): 244 (4.39), 265 (4.45), 281sh (4.37), 401 (3.73); IR (ν_cm_^−1^): 3414, 3245 (NH), 1687 (C=O); ^1^H-NMR [(CD_3_)_2_SO-d_6_]: δ 1.26–1.33 (2H, m, 4′-CH_2_), 1.83–1.90 (4H, m, 3′ and 5′-CH_2_), 2.14–2.29 (4H, m,2′-CH_2_ and 6′-CH_2_), 2.63 (3H, s, 7-Me), 3.04 (1H, m, 1′-CH_2_), 3.95 (1H, br s, NH, D_2_O exchangeable), 7.31 (1H, d, *J_8,9_* = 8.1 Hz,, 9-H), 7.60 (1H, s, 6-H), 7.87 (1H, d, *J_8,9_* = 8.1 Hz,, 8-H), 8.84 (1H, s, 5-H), 10.20 (1H, br s, NH, D_2_O exchangeable); % CHN analysis: Anal. Calcd for C_18_H_20_N_4_O: C, 70.11; H, 6.54; N, 18.17. Found: C, 69.81; H, 6.78; N, 17.97; MSEI, *m*/*z* (%): 308 (M^+^, 16%), 226 (100%).

*2-(2-Hydroxyethylamino)-9-(methyl)pyrimido[4,5-b]quinoline-4(3H)-one* (**3j**): Yield (1.8 g, 67%, DMF-H_2_O); m.p. 200–210 °C; UV (EtOH): λ_max_nm (logε/dm^3^ mol^−1^cm^−1^): 224 (4.27), 265 (4.40), 281sh (4.31), 370 (3.72); IR (ν_cm_^−1^): 3394 (OH), 3235 (NH), 1695 (C=O); ^1^H-NMR [(CD_3_)_2_SO-d_6_]: δ 2.65 (3H, s, 9-Me), 3.51–3.53 (2H, m, NCH_2_), 3.60–3.61 (2H, m, OCH_2_), 4.94 (1H, br s, OH, D_2_O exchangeable), 6.72 (1H, br s, NH, D_2_O exchangeable), 7.33 (1H, t, *J_6,7_* = *J_7,8_* = 7.5 Hz, 7-H), 7.63 (1H, d, *J_7,8_* = 7.5 Hz, 8-H), 7.89 (1H, d, *J_6,7_* = 7.5 Hz, 6-H), 8.84 (1H, s, 5-H), 11.03 (1H, br s, NH, D_2_O exchangeable); % CHN analysis: Anal. Calcd for C_14_H_14_N_4_O_2_: C, 62.21; H, 5.22; N, 20.73. Found: C, 62.49; H, 5.01; N, 20.13; MSEI, *m*/*z* (%): 270 (M^+^, 32%), 252 (31%), 239 (95%), 226 (100%).

*2-Butylamino-9-(methyl)pyrimido[4,5-b]quinoline-4(3H)-one* (**3k**): Yield (2.3 g, 82%, DMF-H_2_O); m.p. 214–216 °C UV (EtOH): λ_max_nm (logε/dm^3^ mol^–1^cm^–1^): 245 (4.54), 264 (4.70), 281sh (4.54), 360 (3.92); IR(ν_cm_^−1^): 3412, 3237 (NH), 1698 (C=O); ^1^H-NMR [(CD_3_)_2_SO-d_6_]:δ 0.93 (3H, t, *J* = 6.9Hz, 2-NH(CH_2_)_3_CH_3_), 1.36–1.38 (2H, m, 2-NH(CH_2_)_2_CH_2_-), 1.52–1.56 (2H, m, 2-NHCH_2_CH_2_-), 2.64 (3H, s, 9-Me), 3.18–3.20 (2H, m, 2-NHCH_2_-), 6.65 (1H, br s, NH, D_2_O exchangeable), 7.31–7.33 (1H, m, 7-H), 7.60–7.62 (1H, m, 8-H), 7.87–7.89 (1H, m, 6-H), 8.86 (1H, s, 5-H), 11.25 (1H, br s, NH, D_2_O exchangeable); % CHN analysis: Anal. Calcd for C_16_H_18_N_4_O: C, 68.06; H, 6.43; N, 19.84. Found: C, 67.83; H, 6.58; N, 20.19; MSEI, *m*/*z* (%): 282 (M^+^, 43%), 266 (4%), 253 (19%), 239 (73%), 226 (100%).

*9-Methyl-2-(propylamino)pyrimido[4,5-b]quinoline-4(3H)-one* (**3l**): Yield (2.1 g, 78%, DMF-H_2_O); m.p. 270–272 °C; UV (EtOH): λ_max_nm (logε/dm^3^ mol^−1^cm^−1^): 244 (4.29), 265 (4.48), 281sh (4.36), 373 (3.56); IR (ν_cm_^−1^): 3369, 3233 (NH), 1695 (C=O); ^1^H-NMR [(CD_3_)_2_SO-d_6_]: δ 0.94–0.95 (3H, m, 2-NH(CH_2_)_2_CH_3_), 1.58–1.60 (2H, m, 2-NHCH_2_CH_2_-), 2.65 (3H, s, 9-Me), 3.37–3.39 (2H, m, 2-NHCH_2_-), 6.63 (1H, br s, NH, D_2_O exchangeable), 7.32 (1H, t, *J_6,7_* = *J_7,8_* = 7.6 Hz, 7-H), 7.61 (1H, d, *J_7,8_* = 7.6 Hz, 8-H), 7.88 (1H, d, *J_6,7_* = 7.6 Hz, 6-H), 8.88 (1H, s, 5-H), 10.99 (1H, br s, NH, D_2_O exchangeable); % CHN analysis: Anal. Calcd for C_15_H_16_N_4_O: C, 67.15; H, 6.01; N, 20.88. Found: C, 67.41; H, 6.37; N, 20.69; MSEI, *m*/*z* (%): 268 (M^+^, 44%), 254 (16%), 240 (79%), 226 (100%).

*2-Cyclohexylamino-9-(methyl)pyrimido[4,5-b]quinoline-4(3H)-one* (**3m**): Yield (2.60 g, 84%, DMF-H_2_O); m.p. 178–180 °C; UV (EtOH): λ_max_nm (logε/dm^3^ mol^−1^cm^−1^): 243 (4.12), 264 (4.27), 281sh (4.10), 378 (3.68); IR (ν_cm_^−1^): 3410, 3220 (NH), 1688 (C=O); ^1^H-NMR [(CD_3_)_2_SO-d_6_]: δ 1.26–1.40 (2H, m, 4’-CH_2_), 1.44–1.72 (4H, m, 3’ and 5’-CH_2_), 1.74–1.96 (4H, m, 2’-CH_2_ and 6’-CH_2_), 2.65 (3H, s, 9-Me), 2.89 (1H, m, 1’-CH), 3.93 (1H, br s, NH, D_2_O exchangeable), 7.32 (1H, t, *J_6,7_* = *J_7,8_* =6 Hz, 7-H), 7.63 (1H, d, *J_7,8_* = 6 Hz, 8-H), 7.89 (1H, d, *J_6,7_* = 6 Hz, 6-H), 8.87 (1H, s, 5-H), 10.23 (1H, br s, NH, D_2_O exchangeable); *%* CHN analysis: Anal. Calcd for C_18_H_20_N_4_O.0.4DMF: C, 68.30; H, 6.81; N, 18.25. Found: C, 68.59; H, 6.64; N, 18.05.

#### 3.1.3. General Procedure for the Preparation of 2-(heterocyclic substituted)pyrimido[4,5-*b*]quinoline-4(3*H*)-ones {2-(heterocyclic substituted)-2-deoxo-5-deazaalloxazines} (4**a**–**g**)

A mixture of 2-(methyl)thiopyrimido[4,5-*b*]quinolin-4(3*H*)-one derivatives (**2**, 0.01 mole) and the appropriate amine (0.2–0.3 mole) in DMF (30 mL) was refluxed 24–30 h with stirring. The generated clear yellow solution was kept overnight in the refrigerator so that yellow crystals could be collected through filtration to get the first crop. Excessive amine was removed from the filtrate by evaporation under vacuo and the residue was treated with ether then water to get the second crop free from amine. The collected solids were dried and recrystallized by a DMF-H_2_O mixture to afford pure products as yellow needles in 64–75% yields.

*2-(Piperidin-1-yl)pyrimido[4,5-b]quinoline-4(3H)-one* (**4a**): Yield (1.80 g, 64%, DMF-H_2_O); m.p. 228–230 °C; UV (EtOH): λ_max_nm (logε/dm^3^ mol^−1^cm^−1^): 263sh (4.41), 283 (4.51), 370 (3.83), 412 (3.86); IR (ν_cm_^−1^): 3420 (NH), 1660 (C=O); ^1^H-NMR [(CD_3_)_2_SO-d_6_]: δ 1.63–1.66 (6H, m, 3′,4′ and 5′-CH_2_), 3.80–3.82 (4H, m, 2′ and 6′-N-CH_2_), 7.48–7.50 (1H, m, 7-H), 7.82–7.85 (2H, m, 8 and 9-H), 8.07–8.09 (1H, m, 6-H), 8.96 (1H, s, 5-H), 11.53 (1H, br s, NH, D_2_O exchangeable); % CHN analysis: Anal. Calcd for C_16_H_16_N_4_O: C, 68.55; H, 5.75; N, 19.99. Found: C, 68.39; H, 5.91; N, 19.76; MS, *m/z* (%): 280 (M^+^, 57%), 251 (66%), 225 (39%), 55 (100%).

*7-Methoxy-2-(morpholin-4-yl)pyrimido[4,5-b]quinoline-4(3H)-one* (**4b**): Yield (2.0 g, 64%, DMF-H_2_O); m.p. 208–210 °C; UV (EtOH): λ_max_nm (logε/dm^3^ mol^–1^cm^–1^): 227 (4.24), 269 (4.43), 276sh (4.41), 401 (3.66); IR (ν_cm_^−1^): 3418 (NH), 1612 (C=O); ^1^H-NMR [(CD_3_)_2_SO-d_6_]: δ 3.22–3.27 (4H, m, 3’ and 5’-NCH_2_), 3.76 (3H, s, 7-OMe), 3.84–3.86 (4H, m, 2’ and 6’-OCH_2_), 7.44–7.47 (2H, m, 6 and 8-H), 7.71–7.73 (1H, m, 9-H), 8.80 (1H, s, 5-H), 11.40 (1H, br s, NH, D_2_O exchangeable); % CHN analysis: Anal. Calcd for C_16_H_16_N_4_O_3_. 0.2H_2_O: C, 60.83; H, 5.23; N, 17.73. Found: C, 61.03; H, 4.98; N, 17.87; MSEI, *m/z* (%): 312 (M^+^, 71%), 255 (100%), 226 (28%).

*9-Methyl-2-(morpholin-4-yl)pyrimido[4,5-b]quinoline-4(3H)-one* (**4c**): Yield (2.1 g, 71%, DMF-H_2_O); m.p. 248–250 °C; UV (EtOH): λ_max_nm (logε/dm^3^ mol^−1^cm^−1^): 244 (4.33), 265 (4.39), 285sh (4.29), 400 (3.65); IR (ν_cm_^−1^): 3419 (NH), 1621 (C=O); ^1^H-NMR [(CD_3_)_2_SO-d_6_]: δ 2.66 (3H, s, 9-Me), 3.25–3.29 (4H, m, 3′ and 5′-NCH_2_), 3.70–3.82 (4H, m, 2′ and 6′-OCH_2_), 7.34–7.36 (1H, m, 7-H), 7.55–7.57 (1H, m, 8-H), 7.96–7.98 (1H, m, 6-H), 8.80 (1H, s, 5-H), 10.40 (1H, br s, NH, D_2_O exchangeable); % CHN analysis: Anal. Calcd for C_16_H_16_N_4_O_2_: C, 64.85; H, 5.44; N, 18.91. Found: C, 65.12; H, 5.24; N, 19.22.

*9-Methyl-2-(piperidin-1-yl)pyrimido[4,5-b]quinoline-4(3H)-one* (**4d**): Yield (1.90 g, 65%, DMF-H_2_O); m.p. 220–222 °C; UV (EtOH): λ_max_nm (logε/dm^3^ mol^−1^cm^−1^): 246 (4.40), 266 (4.47), 288sh (4.34), 401 (3.62); IR (ν_cm_^−1^): 3422 (NH), 1678 (C=O); ^1^H-NMR [(CD_3_)_2_SO-d_6_]: δ 1.50–1.54 (6H, m, 3’, 4’ and 5’-CH_2_), 2.76 (3H, s, 9-Me), 3.73–3.75 (4H, m, 2’ and 6’-NCH_2_), 7.31–7.33 (1H, m, 7-H), 7.62–7.64 (1H, m, 8-H), 7.88–7.90 (1H, m, 6-H), 8.88 (1H, s, 5-H), 11.53 (1H, br s, NH, D_2_O exchangeable); % CHN analysis: Anal. Calcd for C_17_H_18_N_4_O: C, 69.37; H, 6.16; N, 19.03. Found: C, 69.09; H, 6.35; N, 19.31; MSEI, *m*/*z* (%): 294 (M^+^, 16%), 266 (8%), 253 (23%), 239 (25%), 225 (100%).

*2-(4-Phenylpiperazin-1-yl)pyrimido[4,5-b]quinoline-4-ol* (**4e**): Yield (2.40 g, 67%, DMF-H_2_O); m.p. 172–174 °C; UV (EtOH): λ_max_nm (logε/dm^3^ mol^−1^cm^−1^): 261sh (4.62), 281 (4.68), 370 (3.71), 410 (3.99); IR (ν_cm_^−1^): 3421 (OH tautomer of C=O); ^1^H-NMR [(CD_3_)_2_SO-d_6_]: δ 3.03–3.04 (4H, m, 2’ and 6’-NCH_2_), 3.49 (1H, br s, OH, D_2_O exchangeable), 3.95–3.99 (4H, m, 3’ and 5’-NCH_2_), 6.79 (1H, dt, *J_3__′′,4__′′_ = J_4__′′,5__′′_ = 7.2 Hz, J_2__′′,4__′′_ = J_4__′′,6__′′_ = 2.1 Hz,* Ph-pH), 6.94 (2H, dd, *J_2__′′,3__′′_* *= J_5__′′,6__′′_* *= 7.2 Hz, J_2__′′,4__′′_* *= J_4__′′,6__′′_ =2.1 Hz*, Ph-oH), 7.16–7.23 (2H, m, Ph-mH), 7.45 (1H, t, *J_6,7_* = *J_7,8_* = 7.8 Hz, 7-H), 7.76 (1H, t, *J_7,8_* = *J_8,9_* = 7.8 Hz, 8-H), 7.82 (1H, d, *J_6,7_* = 7.8 Hz, 6-H), 8.07 (1H, d, *J_8,9_* = 7.8 Hz 9-H), 8.92 (1H, s, 5-H); % CHN analysis: Anal. Calcd for C_21_H_19_N_5_O: C, 70.57; H, 5.36; N, 19.59. Found: C, 70.23; H, 5.72; N, 19.32; MSEI, *m/z* (%): 357 (M^+^, 4%) and 132 (100%).

*7-Methyl-2-(4-phenylpiperazin-1-yl)pyrimido[4,5-b]quinoline-4-ol* (**4f**): Yield (2.70 g, 73%, DMF-H_2_O); m.p. 279–281 °C; UV (EtOH): λ_max_nm (logε/dm^3^ mol^−1^cm^−1^): 249 (4.46), 286sh (4.15), 356 (3.50), 373 (3.48); IR (ν_cm_^−1^): 3431 (OH tautomer of C=O); ^1^H-NMR [(CD_3_)_2_SO-d_6_]: δ 2.68 (3H, s, 7-Me), 3.27–3.34 (4H, m, 2’ and 6’-NCH_2_), 3.48 (1H, br s, OH, D_2_O exchangeable), 3.93–3.96 (4H, m, 3’ and 5’-NCH_2_), 6.85 (1H, t, *J_3__′′,4__′′_* *= J_4__′′,5__′′_ =7.5 Hz*, Ph-pH), 6.97 (2H, d, *J_2__′′,3__′′_* *= J_5__′′,6__′′_* *= 7.5 Hz*, Ph-oH), 7.22-7.27 (2H, m, Ph-mH), 7.65 (1H, d, *J_8,9_ = 8.6 Hz,* 9-H), 7.92 (1H, d, *J_8,9_ = 8.6 Hz*, 8-H), 8.16 (1H, s, 6-H), 8.93 (1H, s, 5-H); % CHN analysis: Anal. Calcd for C_22_H_21_N_5_O: C, 71.14; H, 5.70; N, 18.85. Found: C, 71.35; H, 5.42; N, 18.47; MSEI, *m*/*z* (%): 371(M^+^, 0.03%, metastable ion), 162 (95%), 121 (100%).

*9-Methyl-2-(4-phenylpiperazin-1-yl)pyrimido[4,5-b]quinoline-4-ol* (**4g**): Yield (2.80 g, 75%, DMF-H_2_O); m.p. 281–283 °C; UV (EtOH): λ_max_nm (logε/dm^3^ mol^−1^cm^−1^): 266 (4.52), 287sh (4.36), 359 (3.67), 370 (3.66); IR (ν_cm_^−1^): 3434 (OH tautomer of C=O); ^1^H-NMR [(CD_3_)_2_SO-d_6_]: δ 2.68 (3H, s, 9-Me), 3.25–3.27 (4H, m, 2’ and 6’-NCH_2_), 3.44 (1H, br s, OH, D_2_O exchangeable), 3.94–3.96 (4H, m, 3’ and 5’-NCH_2_), 6.89 (1H, dt, *J_3__′′,4__′′_ = J_4__′′,5__′′_ = 7.8 Hz, J_2__′′,4__′′_ = J_4__′′,6__′′_ = 2.3 Hz,* Ph-pH), 6.97 (2H, dd, *J_2__′′,3__′′_ = J_5__′′,6__′′_ = 7.8 Hz, J_2__′′,4__′′_ = J_4__′′,6__′′_ = 2.3 Hz*, Ph-oH), 7.17-7.24 (2H, m, Ph-mH), 7.38 (1H, t, *J_6,7_* = *J_7,8_* = 8.1 Hz, 7-H), 7.62 (1H, d, *J_7,8_* = 8.1 Hz, 8-H), 7.91 (1H, d, *J_6,7_* = 8.1 Hz, 6-H), 8.93 (1H, s, 5-H); % CHN analysis: Anal. Calcd for C_22_H_21_N_5_O: C, 71.14; H, 5.70; N, 18.85. Found: C, 69.95; H, 5.42; N, 19.12.

#### 3.1.4. General Procedure for the Preparation of 2-Aminopyrimido[4,5-*b*]quinoline-4(3*H*)-ones {2-amino-2-deoxo-5-deazaalloxazines} (**5a,b**)

A mixture of 2-(methylthio)pyrimido[4,5-*b*]quinolin-4(3*H*)-one derivative (**2**, 4.0 mmole) and ammonium acetate (0.2 mole) were mixed thoroughly and fused at 160–165 *°*C with a vigorous stirring for 0.5–3.0 h. The reaction mixture was refrigerated, mixed with 15 mL of water, neutralized with aqueous ammonia and cooled overnight in refrigerator. The resulting brown crystals were collected by filtration, dried and recrystallized from DMF to give **5a,b** as brown needles in 69% and 83% yields.

*2-Aminopyrimido[4,5-b]quinolin-4(3H)-one* (**5a**): Yield (0.70 g, 83%, DMF); m.p. >300 °C; UV (EtOH): λ_max_nm (logε/dm^3^ mol^−1^cm^−1^): 260sh (4.05), 274 (4.06), 302 (3.94), 401 (3.07); IR (ν_cm_^−1^): 3206, 3050 (NH), 1681 (C=O); ^1^H-NMR [(CD_3_)_2_SO-d_6_]: δ 6.72 (2H, br s, NH, D_2_O exchangeable), 7.41–7.43 (1H, m, 7-H), 7.68–7.71 (2H, m, 6 and 8-H), 7.71–7.73 (1H, m, 9-H), 9.01 (1H, s, 5-H), 10.81 (1H, br s, NH, D_2_O exchangeable); % CHN analysis: Anal. Calcd for C_11_H_8_N_4_O: C, 62.26; H, 3.80; N, 26.40. Found: C, 61.94; H, 4.02; N, 26.21; MSEI, *m*/*z* (%): 213 (M^+^ + 1, 4%), 55 (100%).

*2-Amino-9-(methyl)pyrimido[4,5-b]quinolin-4(3H)-one* (**5b**): Yield (0.62 g, 69%, DMF); m.p. >300 °C; UV (EtOH): λ_max_nm (logε/dm^3^ mol^−1^ cm^−1^): 246 (4.13), 283sh (3.89), 334 (3.46), 401 (3.07); IR (ν_cm_^−1^): 3275, 3060 (NH), 1678 (C=O); ^1^H-NMR [(CD_3_)_2_SO-d_6_]: δ 2.64 (3H, s, 9-Me), 6.74 (2H, br s, 2-NH_2_, exchangeable with D_2_O), *7.36 (1H, t, J_6,7_ =* *J_7,8_= 6.8 Hz, 7-H), 7.64 (1H, d, J_7,8_* *= 6.8 Hz, 8-H), 7.90 (1H, d, J_6,7_* *=6.8 Hz, 6-H)*, 8.87 (1H, s, 5-H), 11.18 (1H, br s, NH, D_2_O exchangeable); % CHN analysis: Anal. Calcd for C_12_H_10_N_4_O: C, 63.71; H, 4.46; N, 24.76. Found: C, 63.94; H, 4.72; N, 24.41; MSEI, *m*/*z* (%): 226 (M^+^, 100%), 184 (10%), 157(18%).

#### 3.1.5. General Procedure for the Preparation of Pyrimido[4,5-b]quinoline-2,4(1*H*,3*H*)-diones{2,4-dioxo-5-deazaalloxzines} (**6a–c**)

A mixture of 2-methylthiopyrimido[4,5-*b*]quinolin-4(3*H*)-one derivatives (**2a**,**b**,**d**, 0.01 mole) and 5 M hydrochloric acid (250 mL) was mixed carefully, then refluxed for 10–15 h. The precipitated yellow crystals were filtered off. The excess acid in the filtrate was removed under vacuo to get the residue. The combined first crop crystals and residue of the filtrate were collected with water then neutralized by aqueous ammonia to pH 7. The precipitated crystals were filtered, washed with water, dried and recrystallized from DMF-H_2_O mixture to afford yellow needles in 62–84%.

*Pyrimido[4,5-b]quinoline-2,4-dione* (**6a**): Yield (1.60 g, 75%, DMF-H_2_O); m.p. >300 °C; UV (EtOH): λ_max_nm (logε/dm^3^ mol^−1^cm^−1^): 219 (4.21), 235 (4.18), 252 (4.26), 307 (3.53), 355 (3.37); IR (ν_cm_^−1^): 3177, 3066 (NH), 1710, 1621 (C=O); ^1^H-NMR [(CD_3_)_2_SO-d_6_]: δ 7.56–7.58 (1H, m, 7-H), 7.85–7.88 (2H, m, 6 and 8-H), 8.16–8.18 (1H, m, 9-H), 9.03 (1H, s, 5-H), *11.39* (1H, br s, NH, D_2_O exchangeable*),* 11.68 (1H, br s, NH, D_2_O exchangeable*)*; % CHN analysis: Anal. Calcd for C_11_H_7_N_3_O_2_: C, 61.97; H, 3.31; N, 19.71. Found: C, 61.72; H, 3.06; N, 19.89.

*7-(Methyl)pyrimido[4,5-b]quinoline-2,4-dione* (**6b**): Yield (1.4 g, 62%, DMF-H_2_O); m.p. >300 °C; UV (EtOH): λ_max_nm (logε/dm^3^ mol^−1^cm^−1^): 223(4.15), 240 (4.17), 254 (4.27), 308 (3.49), 363 (3.32); IR (ν_cm_^−1^): 3205, 3019 (NH), 1703, 1615, (C=O); ^1^H-NMR [(CD_3_)_2_SO-d_6_]: δ 2.14 (3H, s, 7-Me), 7.79–7.81 (2H, m, 8, 9-H), 7.94 (1H, s, 6-H), 8.93 (1H, s, 5-H), *11.49* (1H, br s, 1-NH, D_2_O exchangeable*), 11.76 (*(1H, br s, 3-NH, D_2_O exchangeable*)*; % CHN analysis: Anal. Calcd for C_12_H_9_N_3_O_2_: C, 63.43; H, 3.99; N, 18.49. Found: C, 63.69; H, 4.26; N, 18.72.

*9-(Methyl)pyrimido[4,5-b]quinoline-2,4-dione* (**6c**): Yield (1.90 g, 84%, DMF-H_2_O); m.p. >300 °C; UV (EtOH): λ_max_nm (logε/dm^3^ mol^−1^cm^−1^): 256 (4.41), 262 (4.46), 286 (4.16), 360 (3.67); IR (ν_cm_^−1^): 3217, 3039 (NH), 1712, 1625, (C=O); ^1^H-NMR [(CD_3_)_2_SO-d_6_]: δ 2.17 (3H, s, 9-Me), 7.08–7.11 (1H, m, 7-H), 7.20–7.23 (1H, m, 8-H), 7.26–7.28 (1H, m, 6-H), 8.96 (1H, s, 5-H), 10.93 (1H, br s, 1-NH, D_2_O exchangeable*),* 11.34 (1H, br s, 3-NH, D_2_O exchangeable*)*; % CHN analysis: Anal. Calcd for C_12_H_9_N_3_O_2_: C, 63.43; H, 3.99; N, 18.49. Found: C, 63.39; H, 4.27; N, 18.78.

#### 3.1.6. General Procedure for the Preparation of 6-(*o*-tolylamino)pyrimidin-4(3*H*)-one (7)

To a stirring solution of Raney nickel catalyst (8.0 g, 0.14 mol) in absolute ethanol (100 mL), 6-(*N*-*o*-Tolylamino)-2-methylthiopyrimidin-4(3*H*)-one (**1d**, 0.98g, 4 mmole) was added and the mixture was heated under reflux for 0.5 h. The mixture was filtered while hot to remove the catalyst which was washed well with boiling absolute ethanol (4 × 20 mL). The combined filtrate and washings were evaporated to dryness. The residue was recrystallized from DMF, to afford 0.56 g of compound **7** as colorless needles in 70% yield; m.p. 152–154 °C; UV (EtOH): λ_max_nm (logε/dm^3^ mol*^−^*^1^ cm*^−^*^1^): 242 (4.12), 276 (3.93)*;*IR (ν_cm_^−1^): 3405, 3210(NH), 1637 (C=O); ^1^H-NMR [(CD_3_)_2_SO-d_6_]: δ 2.18 (3H, s, Ph-oMe), 4.83 (1H, s, 5-H), 7.11–7.28 (4H, m, Ph-o,m,pH), 7.80 (1H, s, 2-H), 8.53 (1*H,* br s, 6-NH*,* D_2_O exchangeable), 11.60 *(1H*, br s, 3*-NH*, D_2_O exchangeable); *%* CHN analysis: Anal. Calcd for C_11_H_11_N_3_O.0.4H_2_O: C, 63.39; H, 5.71; N, 20.16. Found: C, 63.13; H, 6.08; N, 19.85.

#### 3.1.7. General Procedure for the Preparation of 9-(methyl)pyrimido[4,5-*b*]quinolin-4-ol {2-deoxo-5-deazaalloxazine} (**8**)

A mixture of 6-(*o*-tolylamino)pyrimidin-4(3*H*)-one (7, 2 g, 0.01 mole) and POCl_3_ (7.7 g, 0.05 mole) in anhydrous DMF (10 mL) was heated at 90 °C with stirring for 2 h. Followed by pouring the mixture onto ice and then neutralized to pH 7 by with aqueous ammonia. The product of yellow crystals was filtered off, washed with cold water, dried and recrystallized from mixture of DMF-H_2_O to give 1.70 g. of compound **8** as pale yellow needles in 80% yield; m.p. 249–251 °C; UV (EtOH): λ_max_nm (logε/dm^3^ mol^−1^cm^−1^): 250 (4.18), 278sh (4.08), 336 (3.55), 365 (3.44); IR (ν_cm_^−1^): 3411 (OH tautomer of C=O); ^1^H-NMR [(CD_3_)_2_SO-d_6_]: δ 2.26 (3H, s, 9-Me), 4.01 (1H, br s, 4-OH, D_2_O exchangeable), 6.53 (1H, s, 2-H), 7.54 (1H, t, *J_6,7_* = *J_7,8_* = 7.2 Hz, 7-H), 7.80 (1H, d, *J_7,8_* = 7.2 Hz, 8-H), 8.09 (1H, d, *J_6,7_* = 7.2 Hz, 6-H), 9.23 (1 H, s, 5-H); % CHN analysis: Anal. Calcd for C_12_H_9_N_3_O: C, 68.24; H, 4.29; N, 19.89. Found: C, 68.66; H, 3.96; N, 20.09; MSEI, *m*/*z* (%): 211(M^+^, 100%), 184 (24%), 155 (49%) and 129 (34%).

### 3.2. Antiproliferative MTT Assay

#### 3.2.1. Antiproliferative Activities for Compounds (**2b**–**d, 3a, d, i, j, k, m, 4a, c**–**g, 5a, b**) Against CCRF-HSB and KB Tumor Cell Lines

Four human cancer cell lines were utilized in this study to carry out cell proliferation assay, including CCRF-HSB-2, KB, MCF-7 and HeLa cell lines. The cell culture was conducted at 37 °C, at 5% CO_2_, 95% air and 100% relative humidity. The use of the cancer cell lines was limited to 20 passages and was checked regularly for Mycoplasma. The modified MTT assay [31] developed by M. Tim [32] was used to determine the in vitro antiproliferative effects of our test compounds against the tumor cell lines. The culture media containing the cancer cell lines were seeded in 96-well microplate at a density of 5 × 10^3^ cells per each well in 100 μL of culture medium. Cells were incubated at 37 °C overnight to allow cell attachment. The test compounds were seeded in triplicates to the wells to get the final concentrations of each compound as 0.0, 0.005, 0.05, 0.5, 5, 25 and 50 μM/mL in 100 μL of media (0.1% DMSO for the blank control). After the incubation, 10 µL of MTT solution (10 mg/mL solution) was added to each well. Then incubated for 4 h at 37 °C, 100 µL of 0.02N HCl, 50% DMF and 20% SDS was used to dissolve the formed MTT-formazan (if any). Plates were shaken for 1 min and the absorbance was examined using a multi-plate reader at λ_570_ nm. The IC_50_ (50% growth inhibition) was determined from the dose-response curve based on the equation: (1 − *T/C*) × 100, where C is the mean OD_570_ of the control group and *T* is that of the treated group.

#### 3.2.2. Antiproliferative Activities for Compounds (**2b**–**e, 3b, c, e, f, g, h, l, 4b, 6a**–**c, 8**) Against MCF-7 and HeLa Tumor Cell Lines

Cells were seeded onto sterile 96-well cell culture plates at densities of 5 × 10^3^/well for HeLa cells and 2 × 10^4^/well for MCF-7 cells. Cells were incubated at 37 °C and 5% CO_2_ for 24 h, after which media was aspirated from cells and 100 µL fresh media with/without treatments were added. Control wells were treated by media with DMSO concentrations equivalent to those of treatment peptides. Cells were further incubated at 37 °C and 5% CO_2_ for 72 h, followed by the addition of MTT (10 µL of a 10 mg/mL solution) to each well. Following 2 h incubation of the plate at 37 °C and 5% CO_2_ and shielded from light with tin foil, the media was removed and the produced formazan crystals were solubilized by the addition of 200 µL of DMSO. The optical density of each well was measured quantitatively at 570 nm (OD_570_) by EL808 HiTeck™ Spectra Thermo plate reader (HiTek Instruments, USA). The IC_50_ (50% growth inhibition) was determined from the dose-response curve based on the equation: (1 − *T/C*) × 100, where C is the mean OD_570_ of the control group and *T* is that of the treated group.

#### 3.3. [γ-^32^P] ATP Radiometric Protein Kinase Assay Method

The protein kinase profiling for compound (**3h**) was carried out following the protocol of KINEXUS Bioinformatics Corporation, Vancouver, British Columbia, Canada. This assay was applied for a single measurement at 10 μM concentration by [γ-^32^P]ATP radioisotope against a panel of 20 protein kinases. Both Imatinib and lapatinib were used as positive references at the same concentration. All experiments have been conducted in a designated radioactive environment and at ambient temperature for 30 min in a total volume of 25 μL in the following sequence: component 1:5.0 μL of the active kinase (~10–50 nM final concentration), component 2:5.0 μL of the substrate solution. component 3: 5.0 μL of a buffer, component 4: 5.0 μL of test and reference at different concentrations or 10% DMSO as a blank control, component 5: 5.0 μL of [γ-^32^P]ATP (from 50 μM stock solution, 0.8 μCi). This assay was started with the addition of [γ-^32^P]ATP to a mixture of other components then incubation for 30 min. The reaction was terminated by adding 10 μL of the mixture onto a MultiScreen phosphocellulose P81 plate, washing 3× by 1% solution of phosphoric acid solution. Using a Trilux scintillation counter, the radioactivity was measured for the test samples in comparison to the blank control which contains all components except that the kinase substrate was replaced by the buffer solution.

### 3.4. Annexin V PI/FITC Apoptosis Assay Using MCF7 Cells

Cell apoptosis assay using MCF7 breast cancer cell lines (1 × 105 cells/well) was carried out by flow cytometry as described [41]. Briefly, MCF7 breast cancer cells were harvested by trypsinization and washed with ice-cold PBS (pH 7.4) twice following treatment with the test compound (3h) at final concentrations of 0, 1, 5, 10 μM for 72 h. Cells have been subsequently incubated with 0.5 mL of Annexin V-FITC/ Propidium iodide (PI) (1.0 mg/mL) solution for 30 min in dark at room temperature following the manufacturer’s protocol. The MCF7 cells were microscopically examined for any potential morphological changes before and after treatment. The floating cells were harvested in tubes and kept on ice, whereas, the remaining cells have been trypsinized to be detached then incubated for 3 min at 37 °C and pooled. At 1200 RPM at a fixed temperature of 40 °C, the cells were centrifuged and the supernatants have been discarded with adding 2mL fresh medium to each tube. Compound **3h** at different concentrations was inoculated using 23 G needle and 1 × 105 MCF7 cells were transferred to new tubes of 12 × 75 mm tubes. The cell solutions were washed with1 mL PBS and again centrifuged, followed by discarding the supernatants and re-suspending the pellets in 100 μL × 1 binding buffer (10 mM HEPES). The stained cells were injected through Becton Dickinson FACScan flow cytometer (BD Biosciences, San Jose, CA, USA) and analyzed for Fluorescein Isothiocyanate-Propidium Iodide *(*FITC and PI) fluorescent signals using FL1 and FL2 signal detector, respectively (λex/em 488/530 nm for FITC and λex/em 535/617 nm for PI). For each sample, 12,000 events were acquired and positive FITC and/or PI cells were quantified by quadrant analysis and calculated using CellQuest Software. The percentage of apoptosis was calculated according to the reported protocol [42] using the equation: (Sample-Control)/Control × 100 to determine the apoptosis and necrosis of the cell populations.

### 3.5. Molecular Docking Study

Autodock 4.2 [38], a molecular grid-based docking program, was used for flexible docking of a fully flexible ligand into a partially flexible protein structure. Autodock scans the binding site looking for the lowest energy binding energy model (s). In this study, the target kinase (PDB code, 1t46) [39] was retrieved solely by using ADS (Accelrys Discovery Studio) visualize v4.0 software (Accelrys Inc., San Diego, CA, USA). The Grid was selected to enclose the native ligand, STI-571 (Imatinib). For each of the docked compounds, the results of top docked runs were analyzed and assigned to the same cluster if their atomic coordinates have a root mean square deviation (RMSD) tolerance of 0.5 Å. Then these clusters were rated from the lowest binding energy to the highest. The analysis was further conducted for the top 10 clusters which revealed significant negative binding energies.

#### 3.5.1. Preparation of Target C-Kit Kinase and Ligands

The 3D structures (PDB format) of the test compounds were constructed using Chem3D Ultra 18.1 software [Cambridge Soft Corporation, USA (2019)]. These structures were energetically minimized by the use of the MOPAC package with 100 iterations and an RMS gradient of 0.10. Before operating with AutoDock Tool (ADT), all hydrogen atoms were added to the 3D protein structures and the ligands as well. Besides, Gasteigercharges (empirical atomic partial charges) were automatically computed by ADT on opening the ligands. To afford all different docking possibilities (conformers), the AutoDock program identifies all the rotatable bonds of the docked ligands. The 3D structure of c-Kit tyrosine kinase in complex with its co-crystallized ligand (STI-571; Imatinib) was retrieved as PDB format from the Protein Data Bank (PDB) web site: https://www.rcsb.org/. Then, all H_2_O molecules, bound ligands were removed deleted from the protein structure. Three amino acids, namely: Glu640, Thr670 and Asp810 were utilized as flexible residues during the docking protocol. The polar hydrogen atoms were added and the Kollman charges were automatically loaded for the protein 3D structure.

#### 3.5.2. Calculation of Autogrid Maps for the C-Kit Kinase Embracing the STI-571 Ligand

Prior to running the docking, AutoGrid maps should be developed. These maps estimate the non-covalent interactions energy between the target c-kit kinase and all probe atoms in different grid points in the macromolecule where the ligand potentially binds. AutoGrid creates about 30 different types of grid maps. The created 3D grids of dimensions of 60 × 60 × 60 Å size (x, y, z) and 0.375 Å spacing centered at 27.696, 26.657 and 39.342 Å encompassing the binding site of the assigned kinase where the co-crystallized ligand; STI-571 was embedded. These grid maps were used to guide the docked ligands into the binding site of the c-kit kinase. Accordingly, in the Autogrid process, the size and position (X, y, z) of the grid box will be determined. Once these parameters have been set, AutoGrid will calculate the grid parameter files for each type of atom within a given area. Followed by running of AutoDock to automatically perform the main docking process using a Lamarckian Genetic Algorithm to generate all possible poses.

As a consequence of AutoDock calculations, the output file with the top 10 conformers of the c-kit kinase-ligand interaction will be generated with different orientations of the flexible residues and the ligands within the binding site. Each pose will be scored by Autodock then ranked according to their binding free energy of interaction.

## 4. Conclusions

In this study, we synthesized and investigated different 5-deazaalloxazine analogs in vitro for their antiproliferative activities against four tumor cell lines, namely CCRF-HSB-2, KB, MCF-7 and HeLa. It was noticed that compounds (**2b**, **2c**, **3e**, **3f**, **3g**, **4b**, **6a** and **6c**) show high selective antiproliferative activity against MCF-7 over HeLa tumor cell lines of IC_50_: 0.22–2.17 µM and >100 µM, respectively. Whereas, other analogs showed the opposite (IC_50_ against HeLa cell lines: 0.33–1.39 µM and IC_50_ against MCF-7 cell lines: 8.49 > 100 µM). On the other hand, Compound 5a exhibited high selective activity against KB over CCRF-HSB-2 tumor cell lines of IC_50_: 1.46 µM and >100 µM, respectively.

Particularly, the most promising lead candidate from our synthesized compounds is 2-(2-hydroxyethylamino)*-*7-methyl-pyrimido[4,5-*b*] quinolin-4(3H)-one analog (**3h**), which revealed significant antitumor activities against both of MCF-7 and HeLa tumor cell lines of IC_50_ 0.17 µM and 2.00 µM, respectively. Additionally, PKs target study of compound **3h** illustrated that the compound **3h** induced multi- targets kinase inhibition including −43% against (FAK), −40% against (CDKI) and −36% against (SCR). Moreover, the Annexin-V/PI apoptotic assay elucidate that compound 3h showed 33% and potentially 140% increase in early and late apoptosis to MCF-7 cells respectively at 10 µM, compared to the control. The AutoDock study of different synthesized compounds was conducted for lead optimization. Nonetheless, the docking result of compound **2b** indicates that this compound is predicted to be a significant lead compound for PTK inhibition by its appropriate fitting into the binding site of c-Kit Kinase. This result is considerable correlated with antiproliferative activity of compound (**2b**) against MCF-7 cancer cell line with 1.79 µM. Herein, we are potentially suggested that the compound (**3h**) considerably a promising lead in comparison to the deazaalloxazine derivatives.

## Figures and Tables

**Table 1:** Elemental composition and relative abundance of the ions in the spectra of compounds **6a**–**c** according to the mass spectral data.

Ions	*m*/*z*	Elemental Composition	% Relative Abundance		
			6a	6b	6c
a [M^+^]	213	C_11_H_7_N_3_O_2_	83	─	─
	227	C_12_H_9_N_3_O_2_	─	100	39
b	170	C_10_H_6_N_2_O	35	─	─
	184	C_11_H_8_N_2_O	─	34	─
c	142	C_9_H_6_N_2_	20	─	─
	156	C_10_H_8_N_2_	─	26	─
d	141	C_9_H_5_N_2_	15	─	─
	155	C_10_H_7_N_2_	─	23	36
e	115	C_8_H_5_N	80	─	─
	129	C_9_H_7_N	─	21	50
f	114	C_8_H_4_N	100	─	─
	128	C_9_H_6_N	─	30	100
g	88	C_7_H_4_	36	─	─
	102	C_8_H_6_	─	21	32
h	87	C_7_H_3_	28	─	─
	101	C_8_H_5_	─	17	44

**Table 2:** The inhibitory activities of the synthesized 5-deazaalloxazine derivatives against CCRF-HSB-2, KB, MCF-7 and HeLa tumor cell lines.

Inhibitory Activity Against Tumor Cell Lines [IC_50_ µM)]					
Compd	CCRF–HSB-2	KB	Compd	MCF-7	HeLa
**2b**	23.66	26.73	**2b**	1.79	>100
**2c**	35.56	30.44	**2c**	1.24	>100
**2d**	>100	>100	**2d**	24.72	3.40
**3a**	47.21	35.47	**2e**	>100	>100
**3d**	14.91	6.93	**3b**	4.40	16.67
**3i**	6.71	6.35	**3c**	8.49	0.57
**3j**	>100	24.9	**3e**	1.10	>100
**3k**	>100	11.62	**3f**	1.74	>100
**3m**	9.17	17.47	**3g**	0.22	>100
**4a**	49.58	36.74	**3h**	**0.17**	2.00
**4c**	39.14	23.79	**3l**	>100	0.13
**4d**	19.41	26.01	**4b**	1.25	>100
**4e**	69.38	28.53	**6a**	2.17	>100
**4f**	50.61	60.30	**6b**	29.45	1.39
**4g**	>100	23.28	**6c**	2.10	>100
**5a**	>100	1.46	**8**	18.22	0.33
**5b**	44.64	73.81	5-FU	5.15	7.76
Ara-c	0.14	0.26			

**Table 3:** The best compounds docked by flexible docking results (AutoDock 4.2), considering the binding free energies (Δ*G*_b_) and inhibition constants (*K*_i_) of 5-deazalloxazine derivatives docked into c-kit PTK (1t46).

Comp.	Δ*G*_b_^a^ (kcal/mol)	*K*_i_^b^	Hydrogen Bonds			RMSD ^c^ (Å)
			Atoms of Comp.	Amino Acids	The Most Relevant Amino Acids	
**2b**	−13.04	274.65 pM	N^10^	HN of D810	D810	1.31
**2e**	−12.60	577.10 pM	3-NH 4-C=O	O=C of I789 HS of C788	-	7.28
**3a**	−11.87	2.00 nM	N^1^ 2-NH Terminal OH	HN of D810 O=C of E640 O=C of D810	E640 D810	0.86
**3d**	−13.07	261.56 pM	2-NH 3-NH 4-C=O	OH of T670 O=C of E671 HN of C673	T670 E671 C673	6.41
**3e**	−12.35	889.03 pM	3-NH Terminal OH	O=C of D810 O=C of E640	E640 D810	3.07
**3h**	−11.07	7.68 nM	3-NH Terminal OH	OH of T670 O=C of E640	E640 T670	6.13
**3i**	−13.71	88.93 pM	3-NH 4-C=O 2-NH	O=C of E671 HN of C673 OH of T670	T670 C673 E671	6.82
**3j**	−11.96	1.72 nM	2-NH Terminal HO	O=C of D677 HN of N680	-	10.33
**3k**	−11.64	2.92 nM	4-C=O	HN of C673	C673	7.82
**3m**	−12.86	373.23 pM	4-C=O	HN of C673	C673	7.71
**4a**	−12.42	782.55 pM	4-C=O	HN of C673	C673	7.44
**4b**	−12.41	806.85 pM	7-O 4-C=O	HO of T670 HN of C673	T670 C673	7.88
**4c**	−12.56	624.08 pM	4-C=O	HN of C673	C673	7.56
**4d**	−12.33	917.97 pM	4-C=O	HN of C673	C673	7.57
**4e**	−11.88	1.97 nM	3-NH 4-C=O	O=C of I789 HS of C788	-	8.91
**4g**	−12.22	1.10 nM	3-NH 4-C=O	O=C of I789 HS of C788	-	8.44
**5a**	−11.86	2.03 nM	4-C=O 3-NH 2-NH^1^ 2-NH^1^	HN of K623 O=C of D810 O=C of D810 O=C of E640	E640 D810	2.46
**5b**	−10.45	21.92 nM	4-C=O	HN of C673	C673	8.04
**STI** ^d^	−16.42	915.03 fM	HNC=O Ph-NH	HN of D810 OH of T670	T670 D810	0.17

^a^ Binding free energy, ^b^ Inhibition constant, ^c^ Root mean square deviation, ^d^ Imatinib: the co-crystallized ligand.

## Supplementary Materials

The following are available online.
