# Supplementary material for: Design, Synthesis, Antitumor Activity and Molecular Docking Study of Novel 5-Deazaalloxazine Analogs
Source: Molecules. 2020 May 28;25(11):2518. doi: 10.3390/molecules25112518 (PMC7321323; doi:10.3390/molecules25112518)
Supplement: Supplementary file 1 [file molecules-25-02518-s001.pdf]

SawsanAhmad-S3-DMSO-H

Pulse Sequence: s2pul

Solvent: DMSO
Temp. 50.0 C / 323.1 K
Mercury-300BB "NMR300"

Relax. delay 1.000 sec
Pulse 74.1 degrees
Acq. time 4.004 sec
Width 8000.0 Hz
16 repetitions
OBSERVE H1, 300.0687855 MHz
DATA PROCESSING
FT size 65536
Total time 1 min, 36 sec

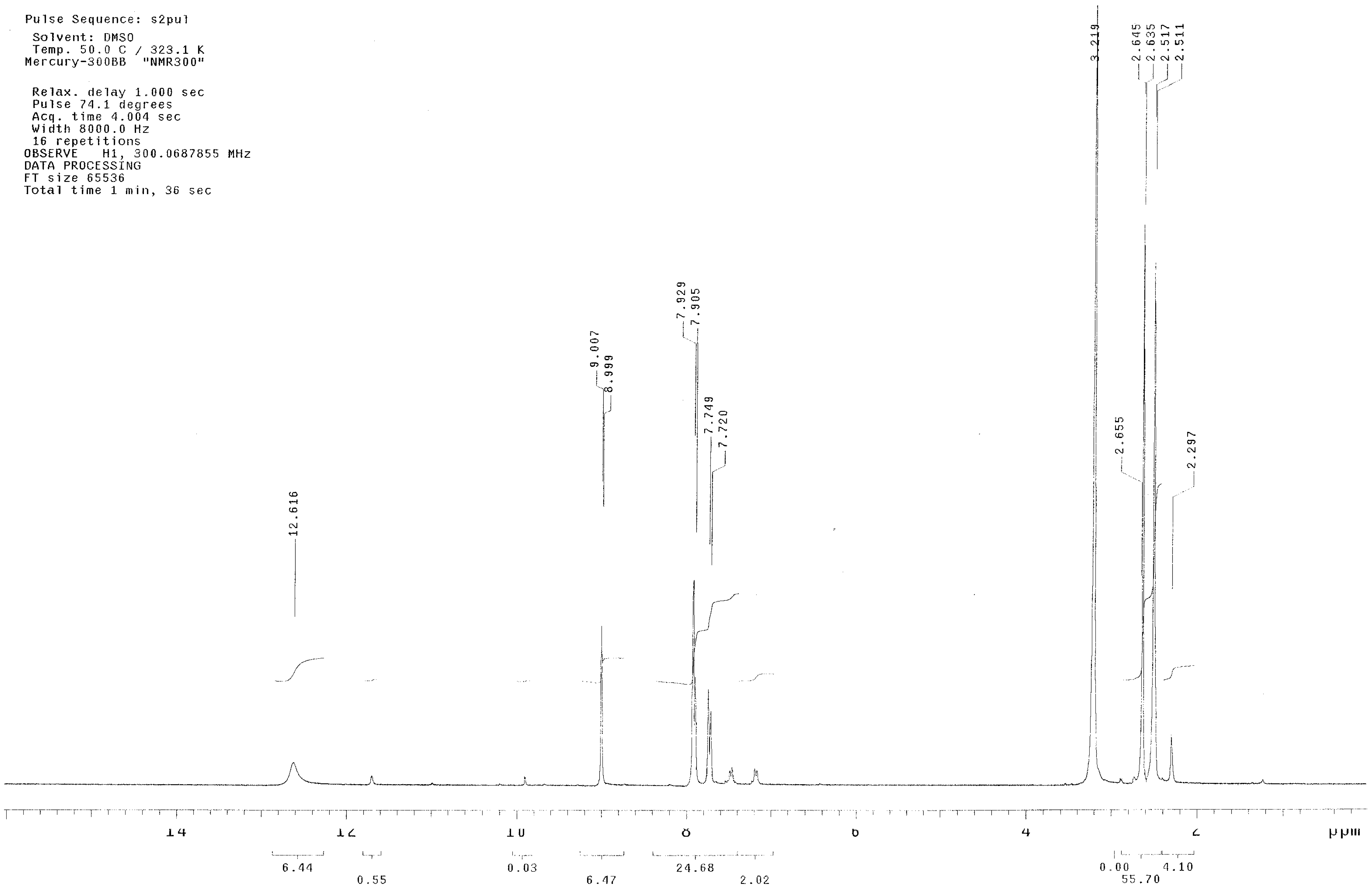

SaswanAhmad-SA8-CDCL3-H

Pulse Sequence: s2pul

Solvent: CDCl3
Temp. 30.0 C / 303.1 K
Mercury-300BB "NMR300"

Relax. delay 1.000 sec
Pulse 74.1 degrees
Acq. time 4.007 sec
Width 9000.0 Hz
32 repetitions
OBSERVE H1, 300.0673626 MHz
DATA PROCESSING
FT size 131072
Total time 31 min, 41 sec

2e NMR

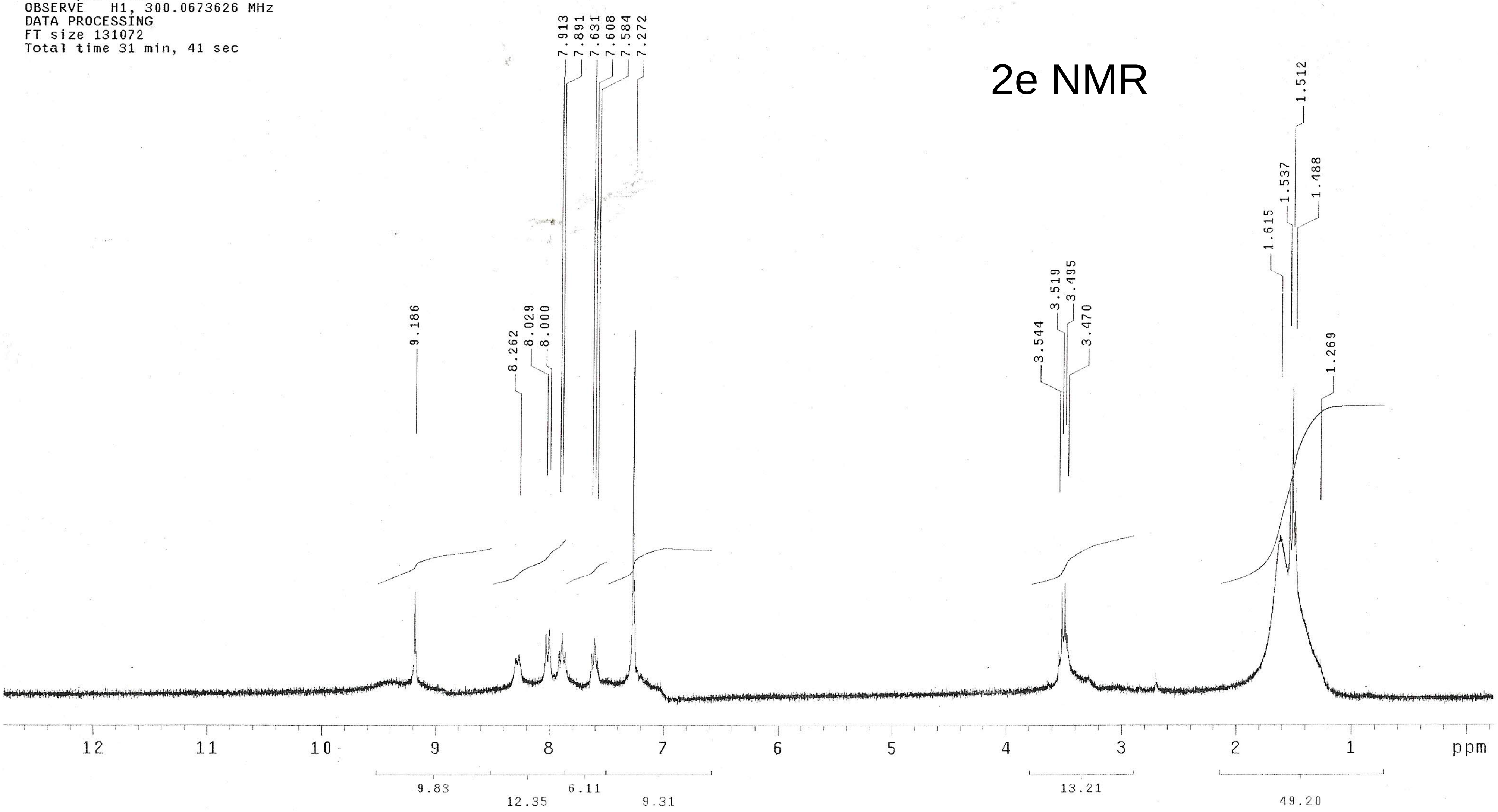

SawssanAhmad-S-11-DMSO-D2O

Pulse Sequence: s2pul

Solvent: DMSO
Temp. 30.0 C / 303.1 K
Mercury-300BB "NMR300"

Relax. delay 1.000 sec
Pulse 74.1 degrees
Acq. time 4.004 sec
Width 8000.0 Hz
8 repetitions
OBSERVE H1, 300.0687855 MHz
DATA PROCESSING
FT size 65536
Total time 19 min, 19 sec

3a NMR d2o

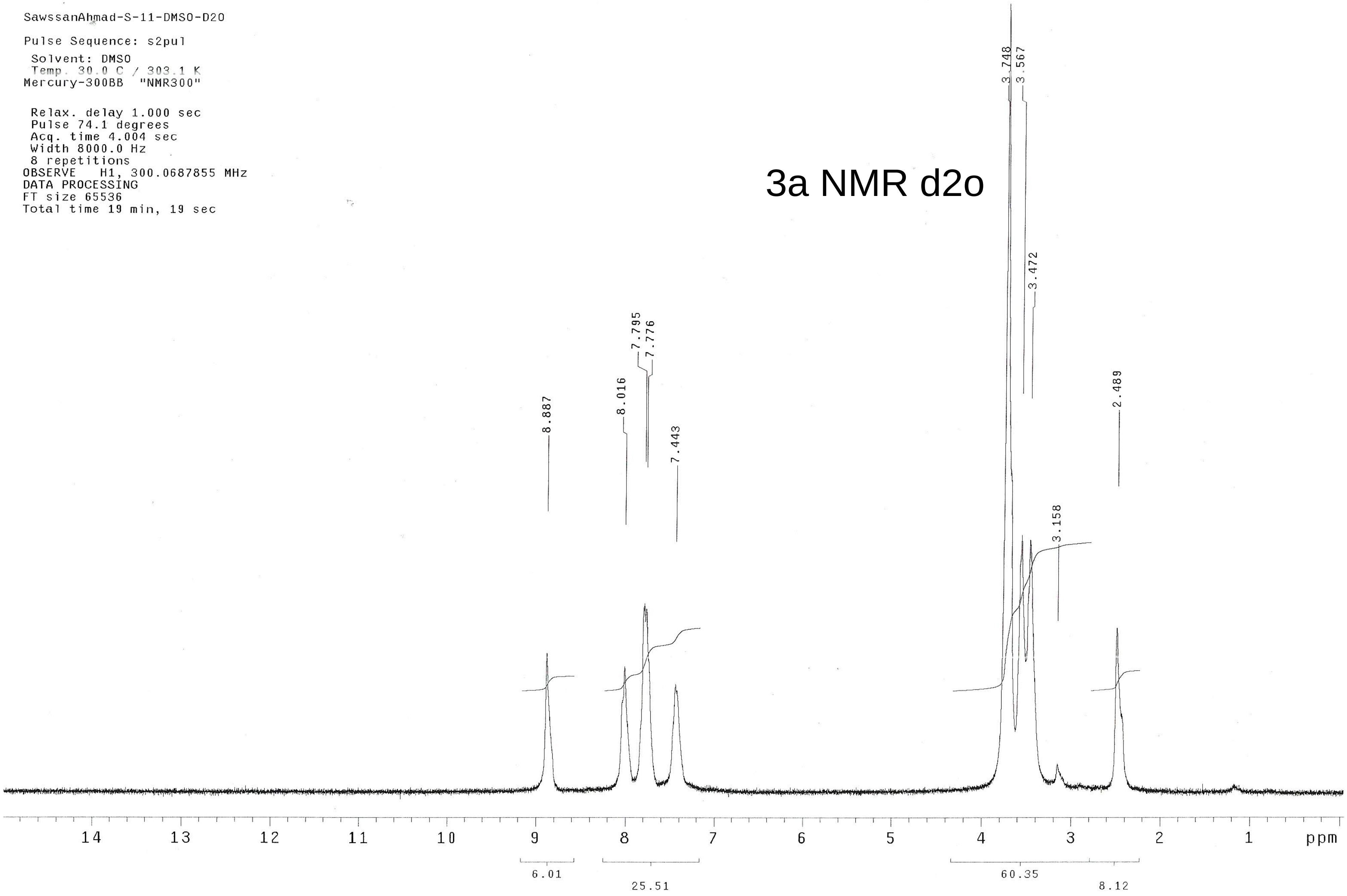

SawssanAhmad-S-12-DMSO-H

Pulse Sequence: s2pul

Solvent: DMSO
Temp. 30.0 C / 303.1 K
Mercury-300BB "NMR300"

Relax. delay 1.000 sec
Pulse 74.1 degrees
Acq. time 4.004 sec
Width 8000.0 Hz
11 repetitions
OBSERVE H1, 300.0687855 MHz
DATA PROCESSING
FT size 65536
Total time 19 min, 19 sec

3c NMR

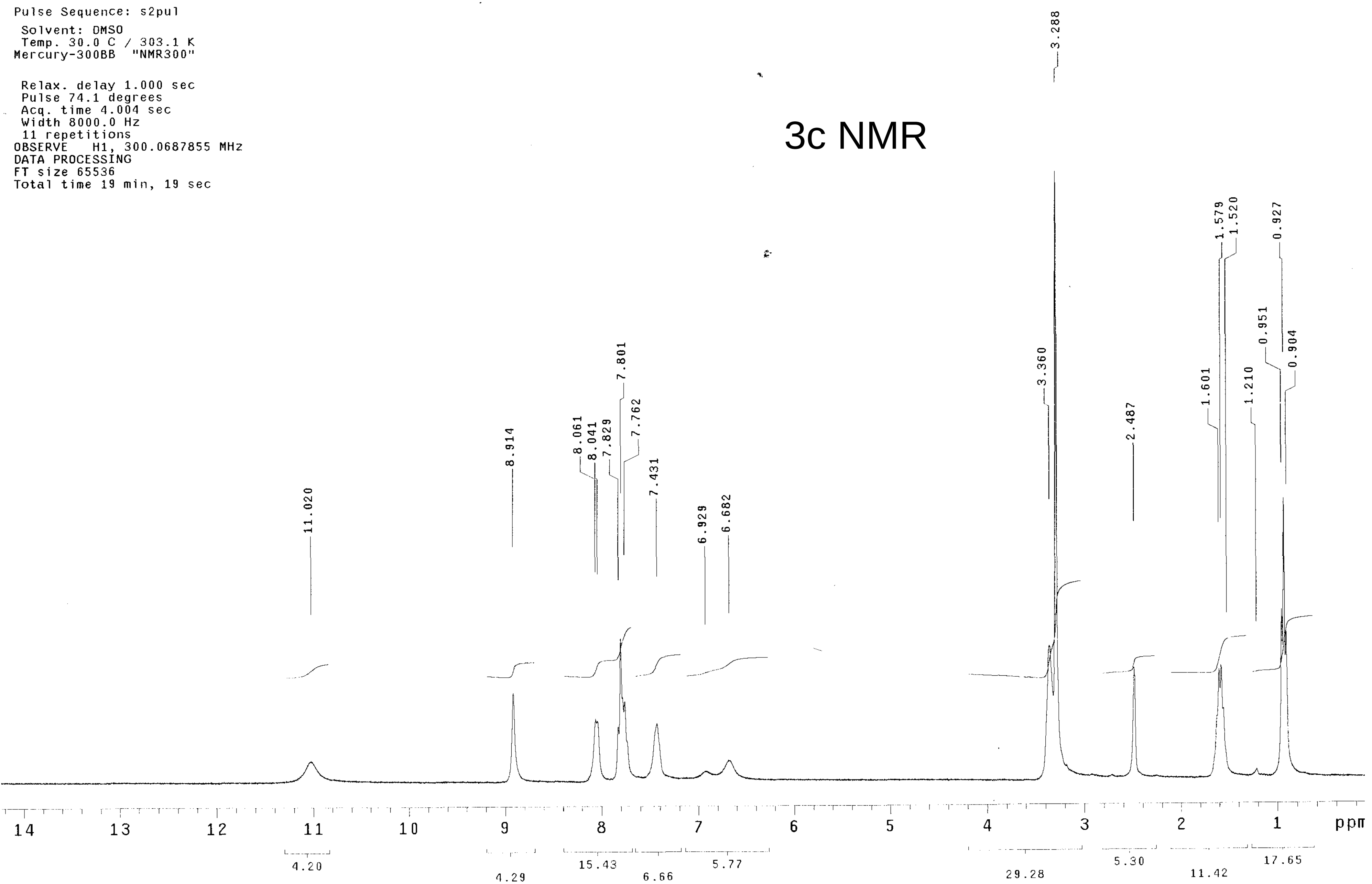

SawsanAhmad-S77-DMSO-H1

Pulse Sequence: s2pul

Solvent: DMSO
Temp. 50.0 C / 323.1 K
Mercury-300BB "NMR300"

Relax. delay 1.000 sec
Pulse 74.1 degrees
Acq. time 4.004 sec
Width 8000.0 Hz
47 repetitions
OBSERVE H1, 300.0687855 MHz
DATA PROCESSING
FT size 65536
Total time 19 min, 19 sec

3d NMR

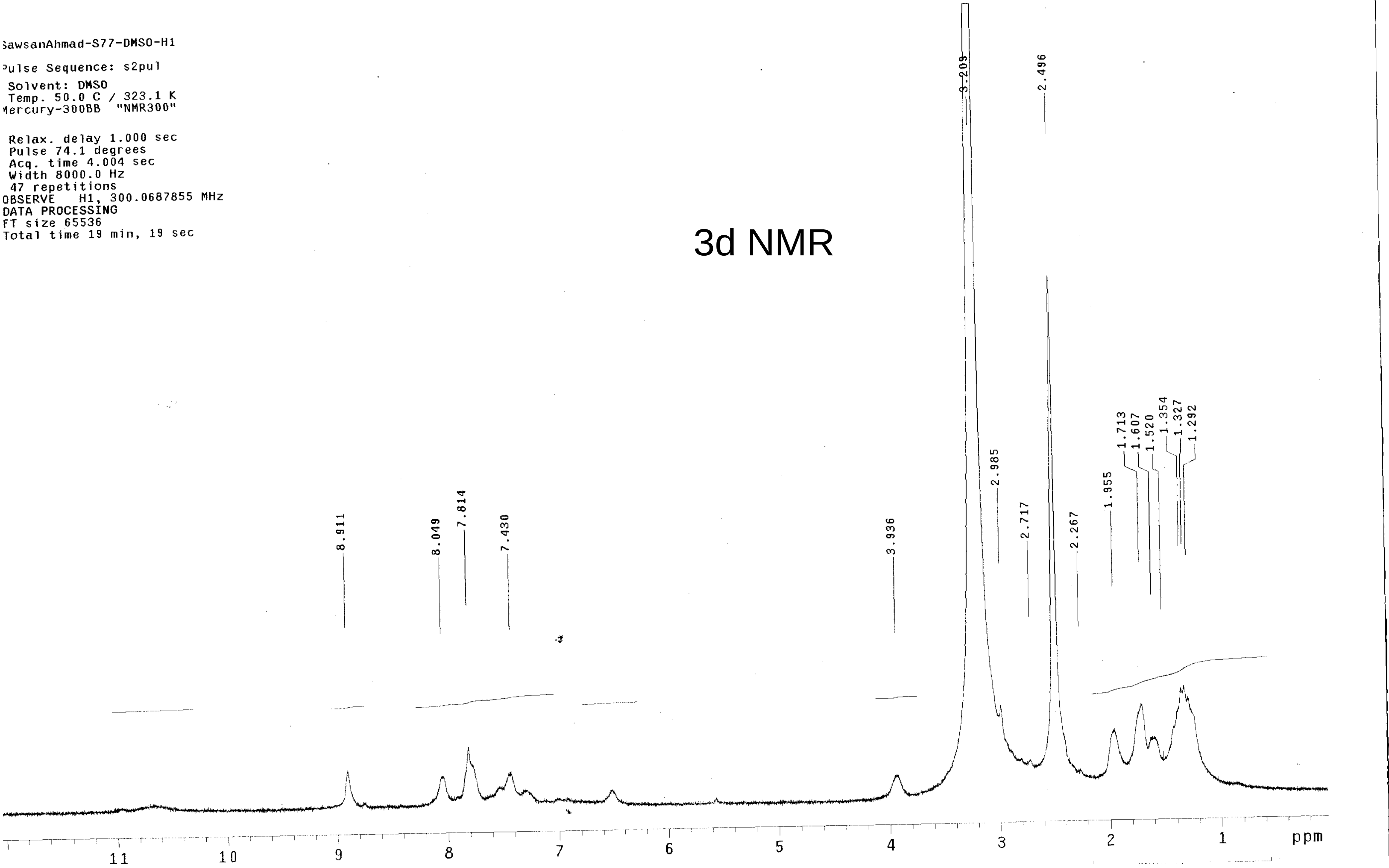

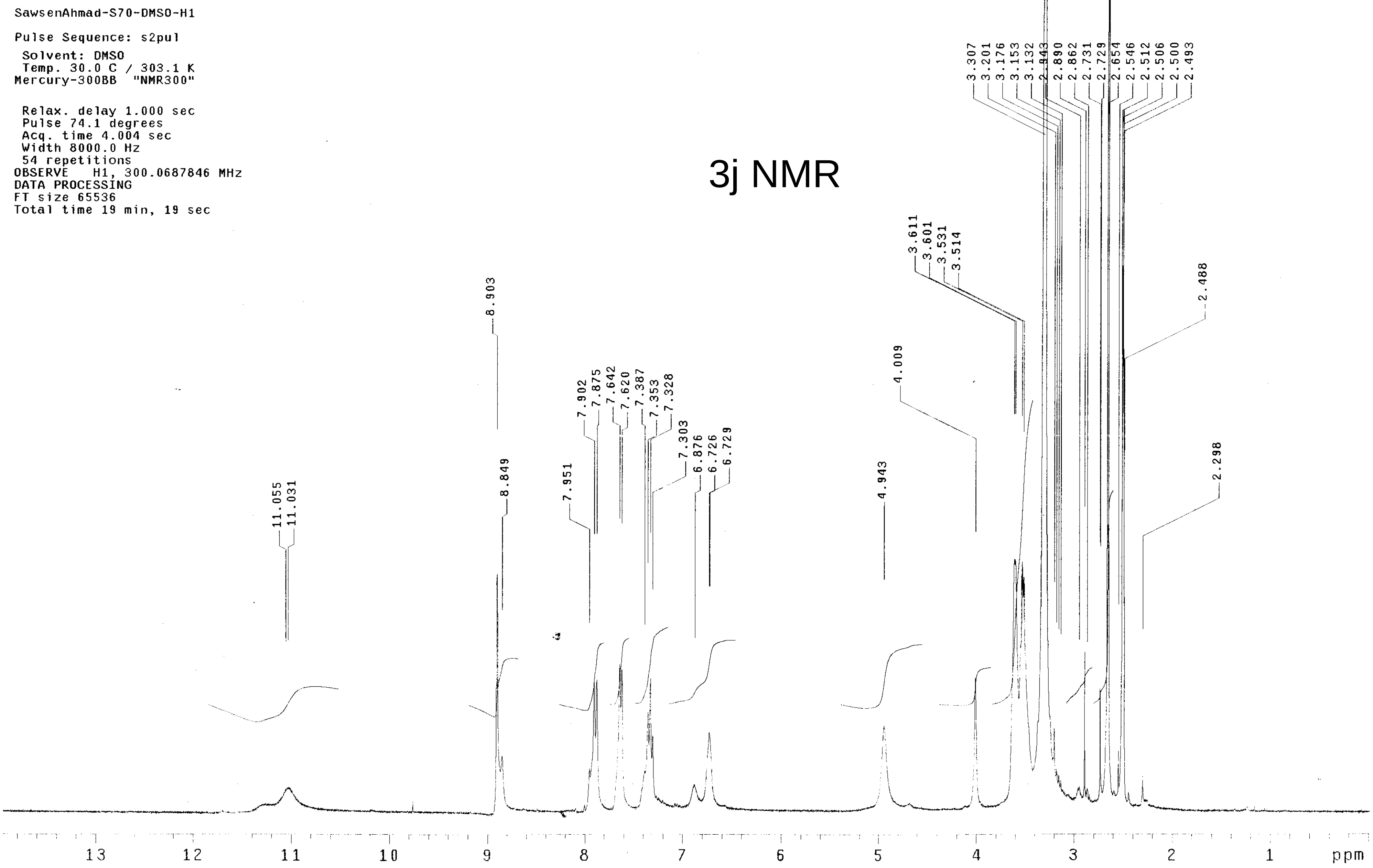
SawsenAhmad-S70-DMSO-H1
Pulse Sequence: s2pul
Solvent: DMSO
Temp. 30.0 C / 303.1 K
Mercury-300BB "NMR300"
Relax. delay 1.000 sec
Pulse 74.1 degrees
Acq. time 4.004 sec
Width 8000.0 Hz
54 repetitions
OBSERVE H1, 300.0687846 MHz
DATA PROCESSING
FT size 65536
Total time 19 min, 19 sec
3j NMR
11.055
11.031
8.903
8.849
7.951
7.902
7.875
7.642
7.620
7.387
7.353
7.328
7.303
6.876
6.726
6.729
4.943
4.009
3.611
3.601
3.531
3.514
3.307
3.201
3.176
3.153
3.132
2.943
2.890
2.862
2.731
2.729
2.654
2.546
2.512
2.506
2.500
2.493
2.488
2.298
13
12
11
10
9
8
7
6
5
4
3
2
1
ppm

3k NMR

SawsanAhmad-S76-DMSO-H1

Pulse Sequence: s2pul

Solvent: DMSO
Temp. 30.0 C / 303.1 K
Mercury-300BB "NMR300"

Relax. delay 1.000 sec
Pulse 74.1 degrees
Acq. time 4.004 sec
Width 8000.0 Hz
36 repetitions
OBSERVE H1, 300.0687855 MHz
DATA PROCESSING
FT size 65536
Total time 19 min, 19 sec

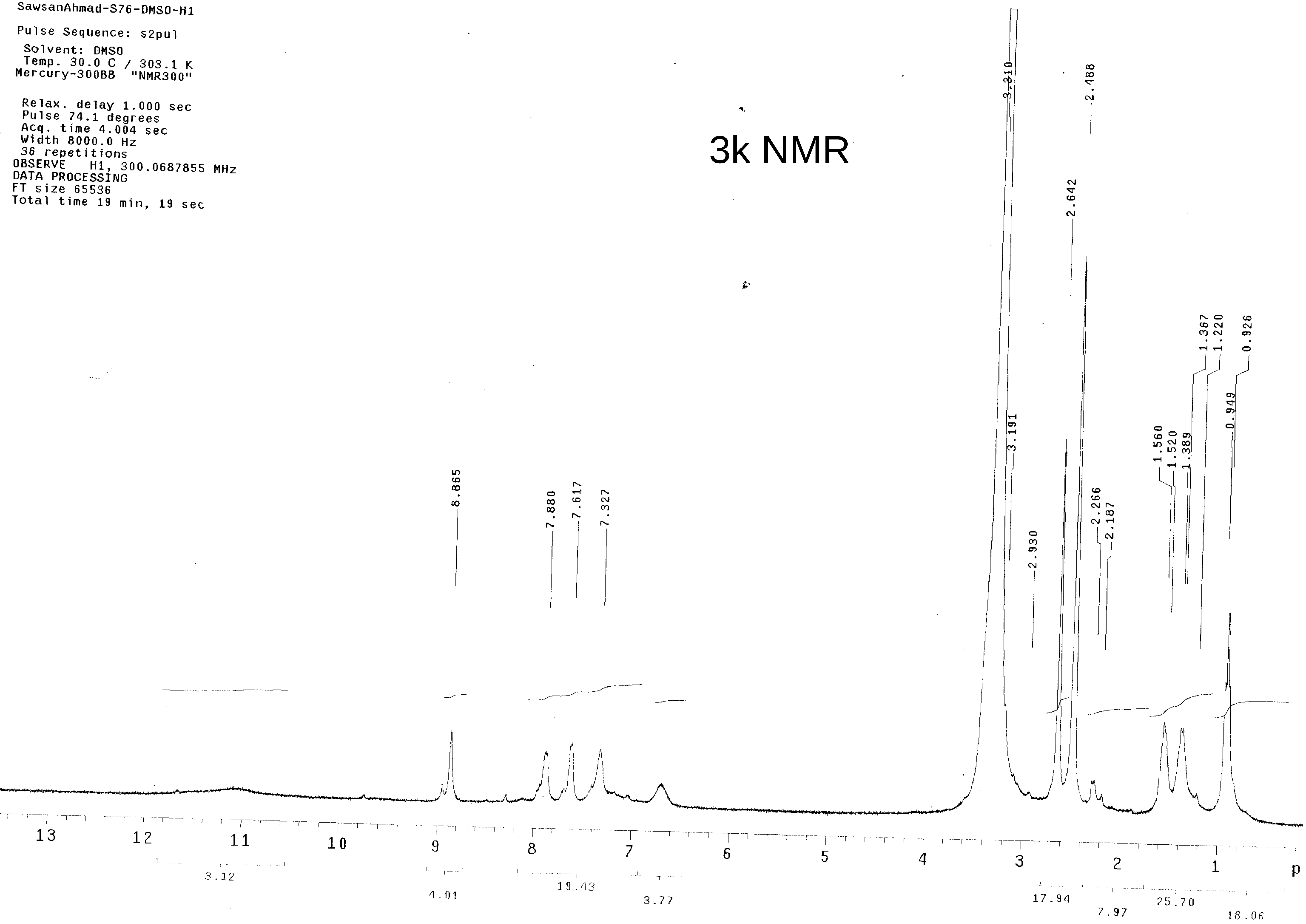

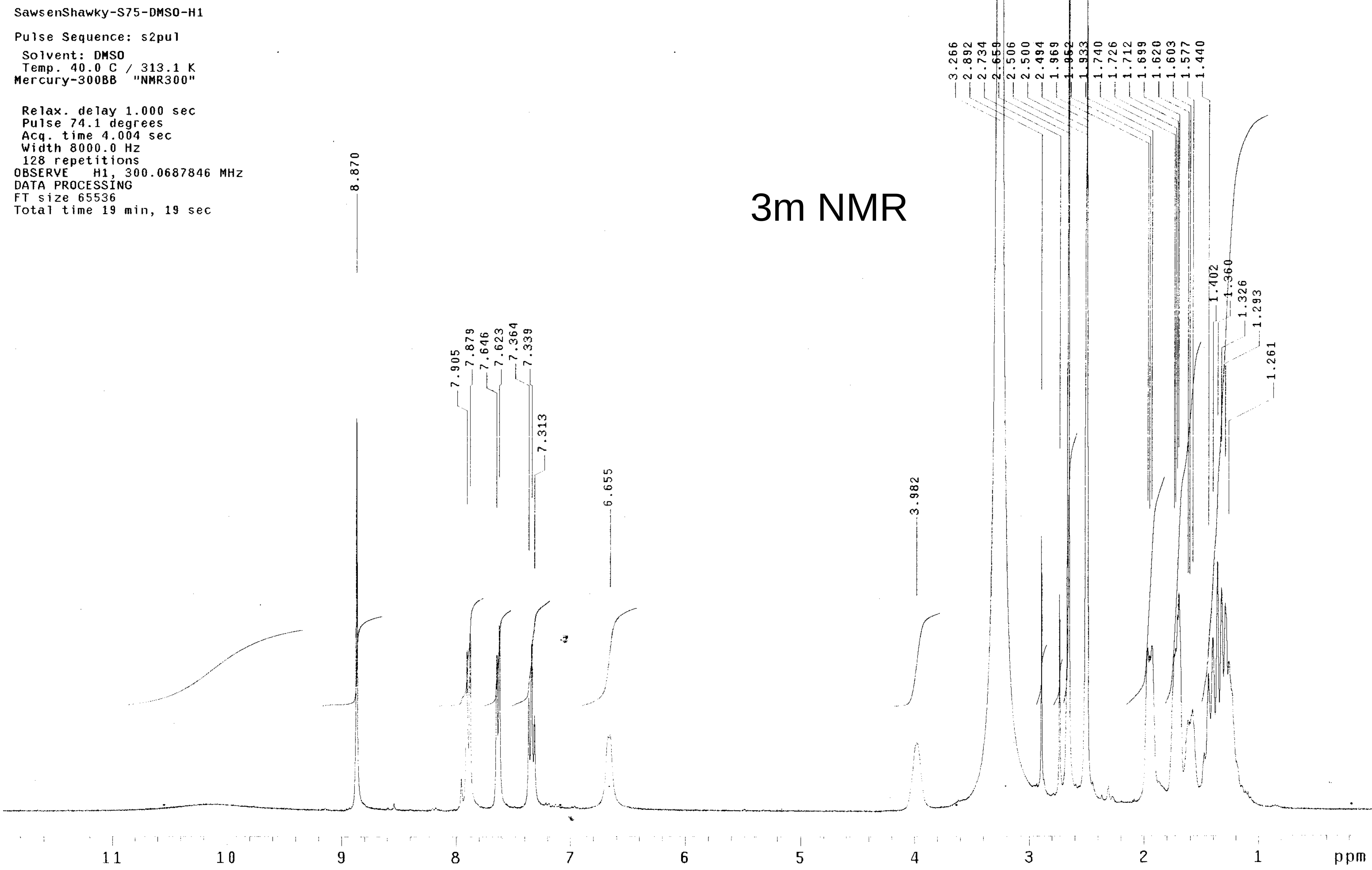
SawsenShawky-S75-DMSO-H1
Pulse Sequence: s2pul
Solvent: DMSO
Temp. 40.0 C / 313.1 K
Mercury-300BB "NMR300"
Relax. delay 1.000 sec
Pulse 74.1 degrees
Acq. time 4.004 sec
Width 8000.0 Hz
128 repetitions
OBSERVE H1, 300.0687846 MHz
DATA PROCESSING
FT size 65536
Total time 19 min, 19 sec
3m NMR
8.870
7.905
7.879
7.646
7.623
7.364
7.339
7.313
6.655
3.982
3.266
2.892
2.734
2.506
2.500
2.494
1.969
1.933
1.740
1.726
1.712
1.699
1.620
1.603
1.577
1.440
1.402
1.360
1.326
1.293
1.261
11
10
9
8
7
6
5
4
3
2
1
ppm

SawssanAhmad-S-22-DMSO-H

Pulse Sequence: s2pul

Solvent: DMSO
Temp. 30.0 C / 303.1 K
Mercury-300BB "NMR300"

Relax. delay 1.000 sec
Pulse 74.1 degrees
Acq. time 4.004 sec
Width 8000.0 Hz
31 repetitions
OBSERVE H1, 300.0687855 MHz
DATA PROCESSING
FT size 65536
Total time 19 min, 19 sec

4b NMR

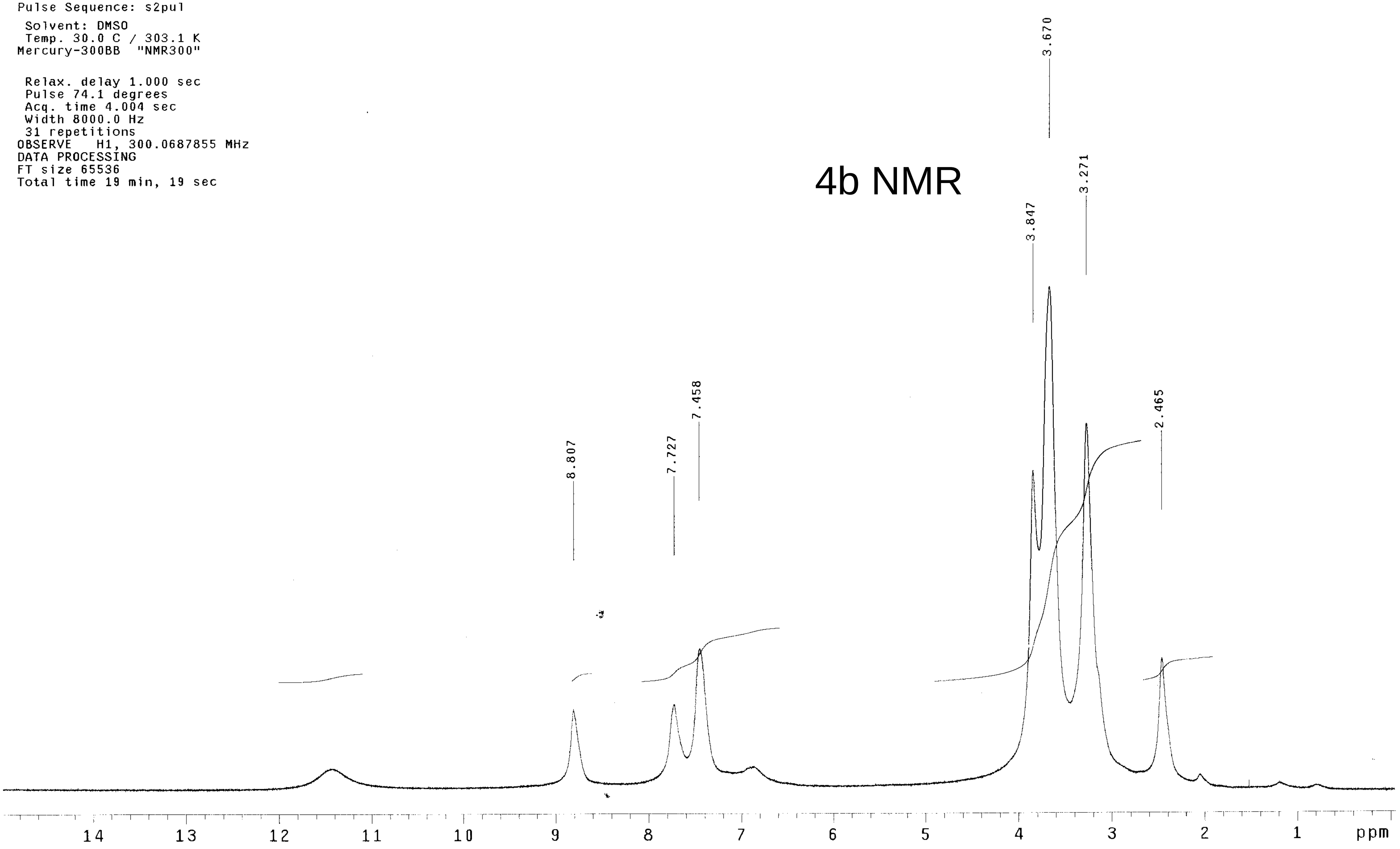

SawsanAhmad-S2-DMSO-H

Pulse Sequence: s2pul

Solvent: DMSO
Temp. 30.0 C / 303.1 K
Mercury-300BB "NMR300"

Relax. delay 1.000 sec
Pulse 74.1 degrees
Acq. time 4.004 sec
Width 8000.0 Hz
32 repetitions
OBSERVE H1, 300.0687855 MHz
DATA PROCESSING
FT size 65536
Total time 3 min, 13 sec

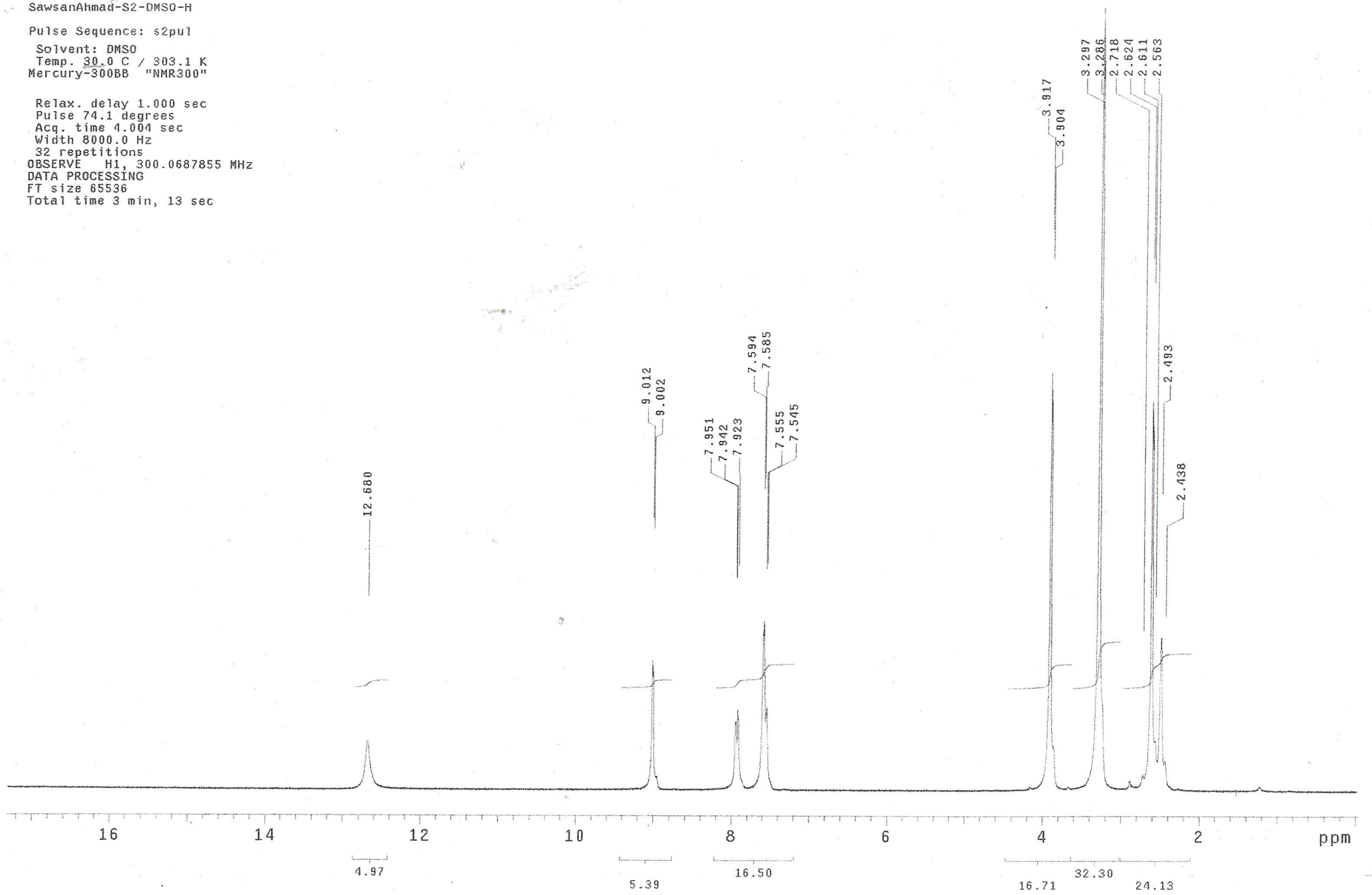

SawsanAhmad-S-116-DMSO-H

Pulse Sequence: s2pul

Solvent: DMSO
Temp. 30.0 C / 303.1 K
Mercury-300BB "NMR300"

Relax. delay 1.000 sec
Pulse 74.1 degrees
Acq. time 4.004 sec
Width 8000.0 Hz
16 repetitions
OBSERVE H1, 300.0687855 MHz
DATA PROCESSING
FT size 65536
Total time 19 min, 19 sec

4e

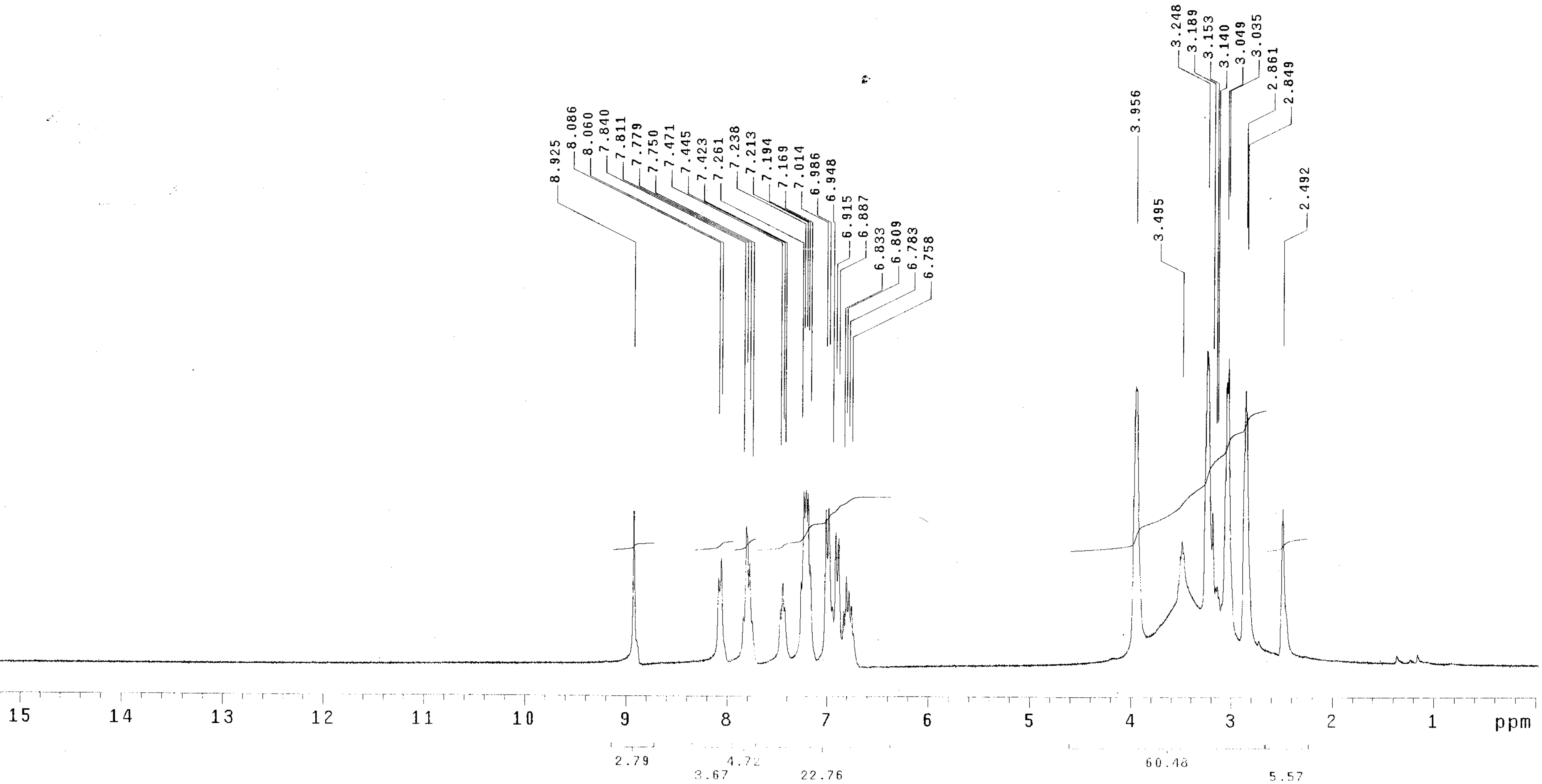

SawssanAhmad-S-11-DMSO-H

Pulse Sequence: s2pul

Solvent: DMSO
Temp. 30.0 C / 303.1 K
Mercury-300BB "NMR300"

Relax. delay 1.000 sec
Pulse 74.1 degrees
Acq. time 4.004 sec
Width 8000.0 Hz
9 repetitions
OBSERVE H1, 300.0687855 MHz
DATA PROCESSING
FT size 65536
Total time 19 min, 19 sec

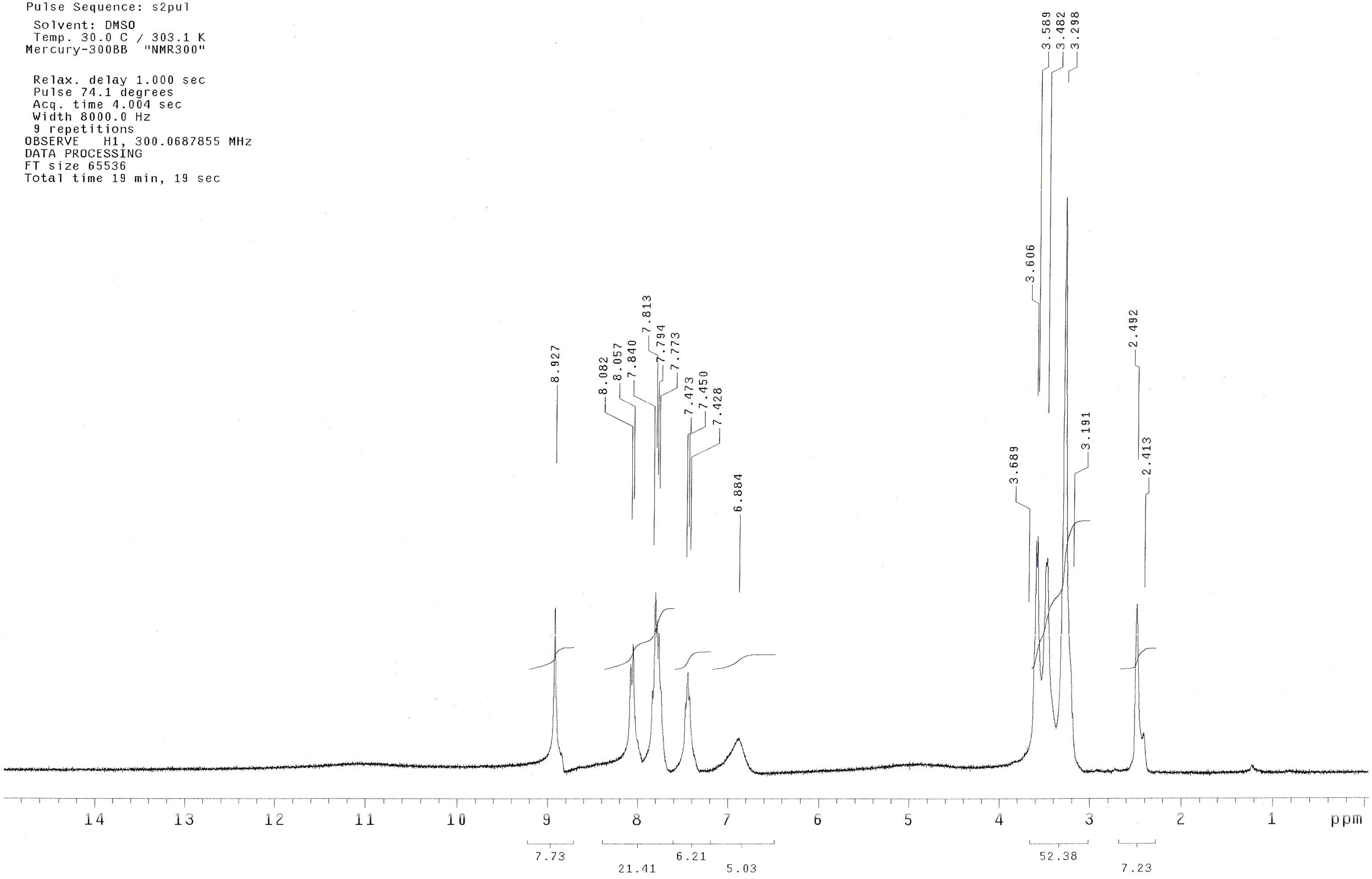

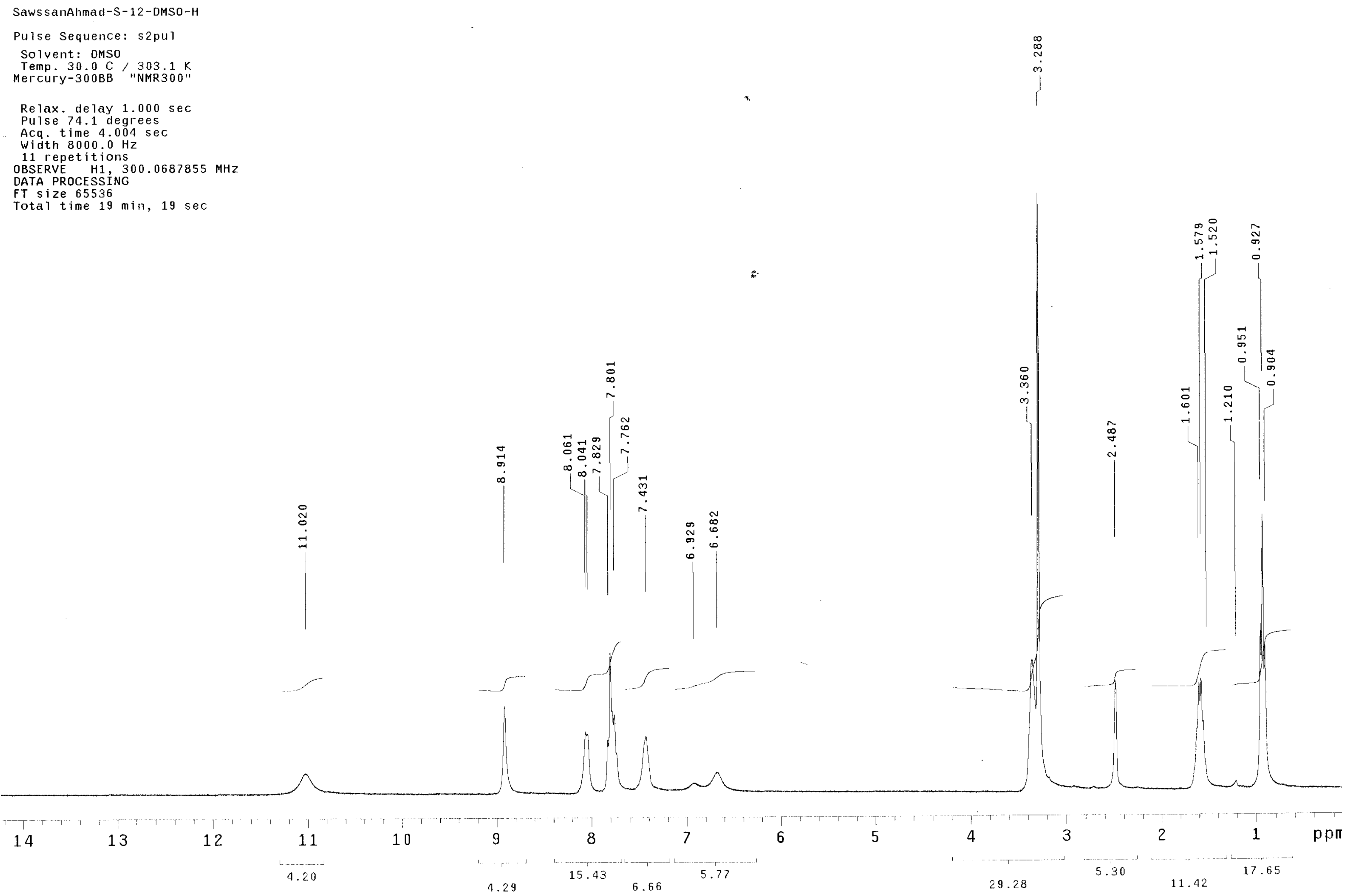
SawssanAhmad-S-12-DMSO-H
Pulse Sequence: s2pul
Solvent: DMSO
Temp. 30.0 C / 303.1 K
Mercury-300BB "NMR300"
Relax. delay 1.000 sec
Pulse 74.1 degrees
Acq. time 4.004 sec
Width 8000.0 Hz
11 repetitions
OBSERVE H1, 300.0687855 MHz
DATA PROCESSING
FT size 65536
Total time 19 min, 19 sec
11.020
8.914
8.061
8.041
7.829
7.801
7.762
7.431
6.929
6.682
3.360
3.288
2.487
1.601
1.579
1.520
1.210
0.951
0.927
0.904
14
13
12
11
10
9
8
7
6
5
4
3
2
1
ppm
4.20
4.29
15.43
6.66
5.77
29.28
5.30
11.42
17.65

SawsanAhmad-S77-DMSO-H1

Pulse Sequence: s2pul

Solvent: DMSO
Temp. 50.0 C / 323.1 K
Mercury-300BB "NMR300"

Relax. delay 1.000 sec
Pulse 74.1 degrees
Acq. time 4.004 sec
Width 8000.0 Hz
47 repetitions
OBSERVE H1, 300.0687855 MHz
DATA PROCESSING
FT size 65536
Total time 19 min, 19 sec

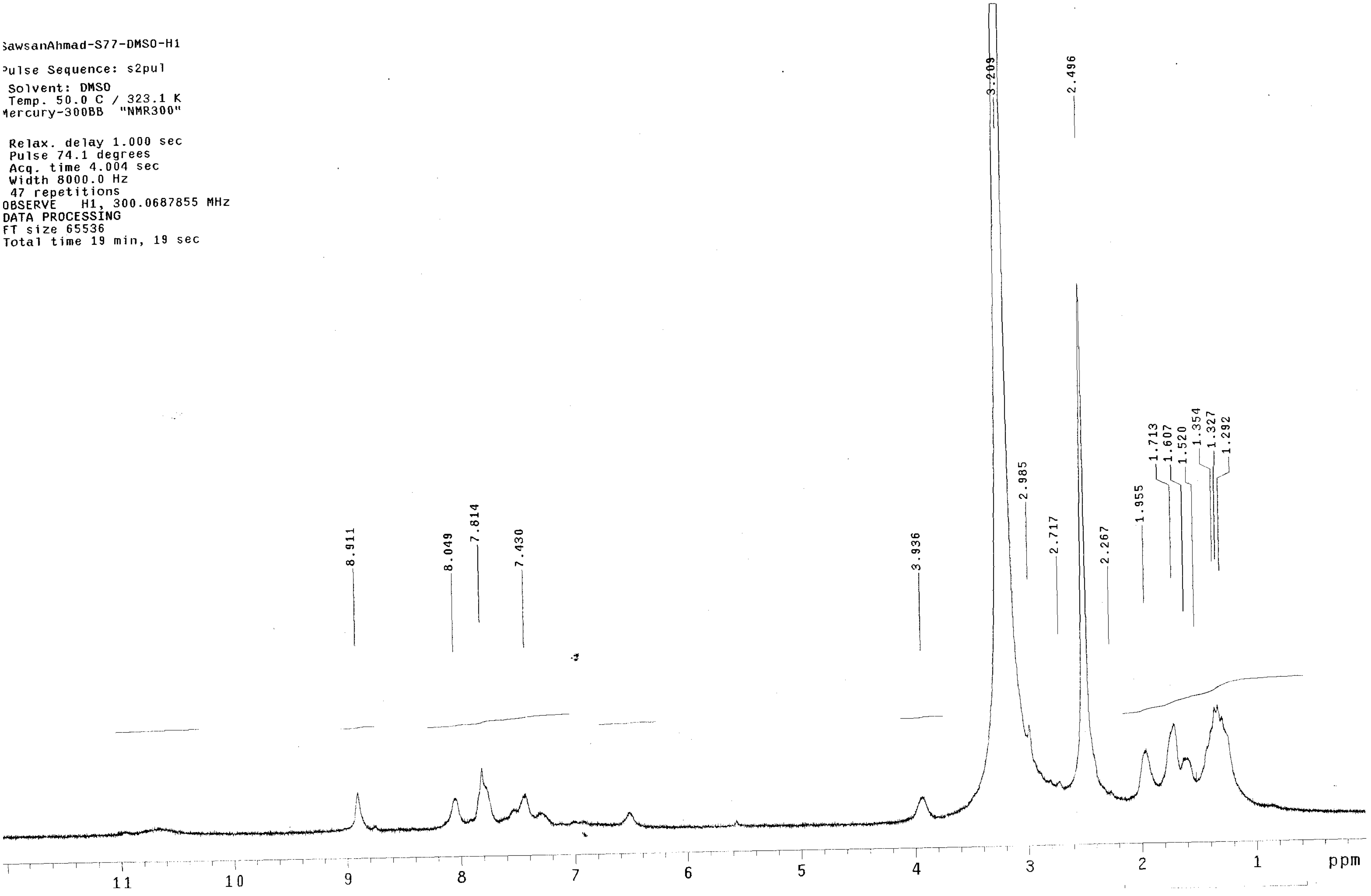

SawsanAhmad-S76-DMSO-H1

Pulse Sequence: s2pul

Solvent: DMSO
Temp. 30.0 C / 303.1 K
Mercury-300BB "NMR300"

Relax. delay 1.000 sec
Pulse 74.1 degrees
Acq. time 4.004 sec
Width 8000.0 Hz
36 repetitions
OBSERVE H1, 300.0687855 MHz
DATA PROCESSING
FT size 65536
Total time 19 min, 19 sec

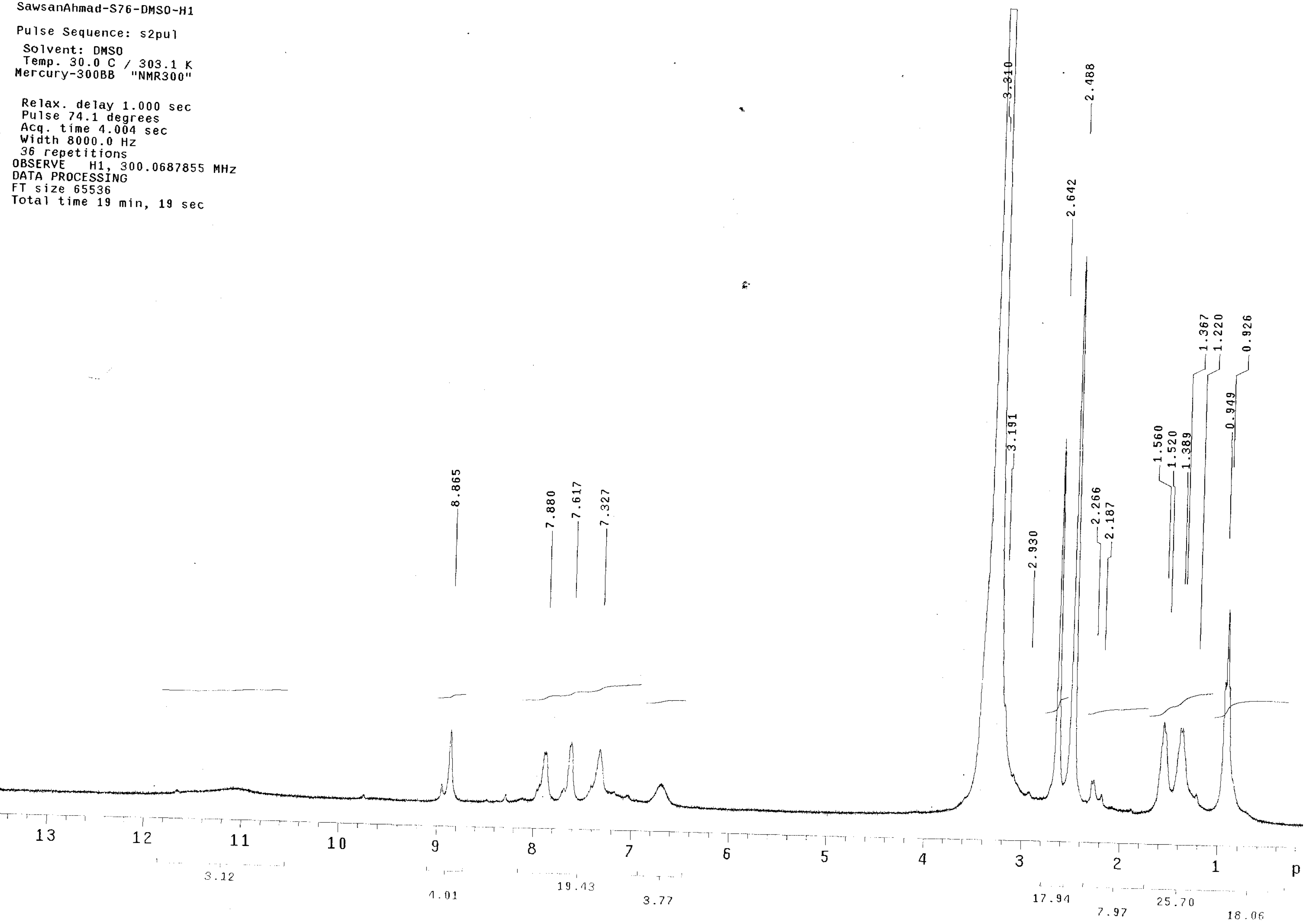

SawsanAhmad-S-116-DMSO-H

Pulse Sequence: s2pul

Solvent: DMSO
Temp. 30.0 C / 303.1 K
Mercury-300BB "NMR300"

Relax. delay 1.000 sec
Pulse 74.1 degrees
Acq. time 4.004 sec
Width 8000.0 Hz
16 repetitions
OBSERVE H1, 300.0687855 MHz
DATA PROCESSING
FT size 65536
Total time 19 min, 19 sec

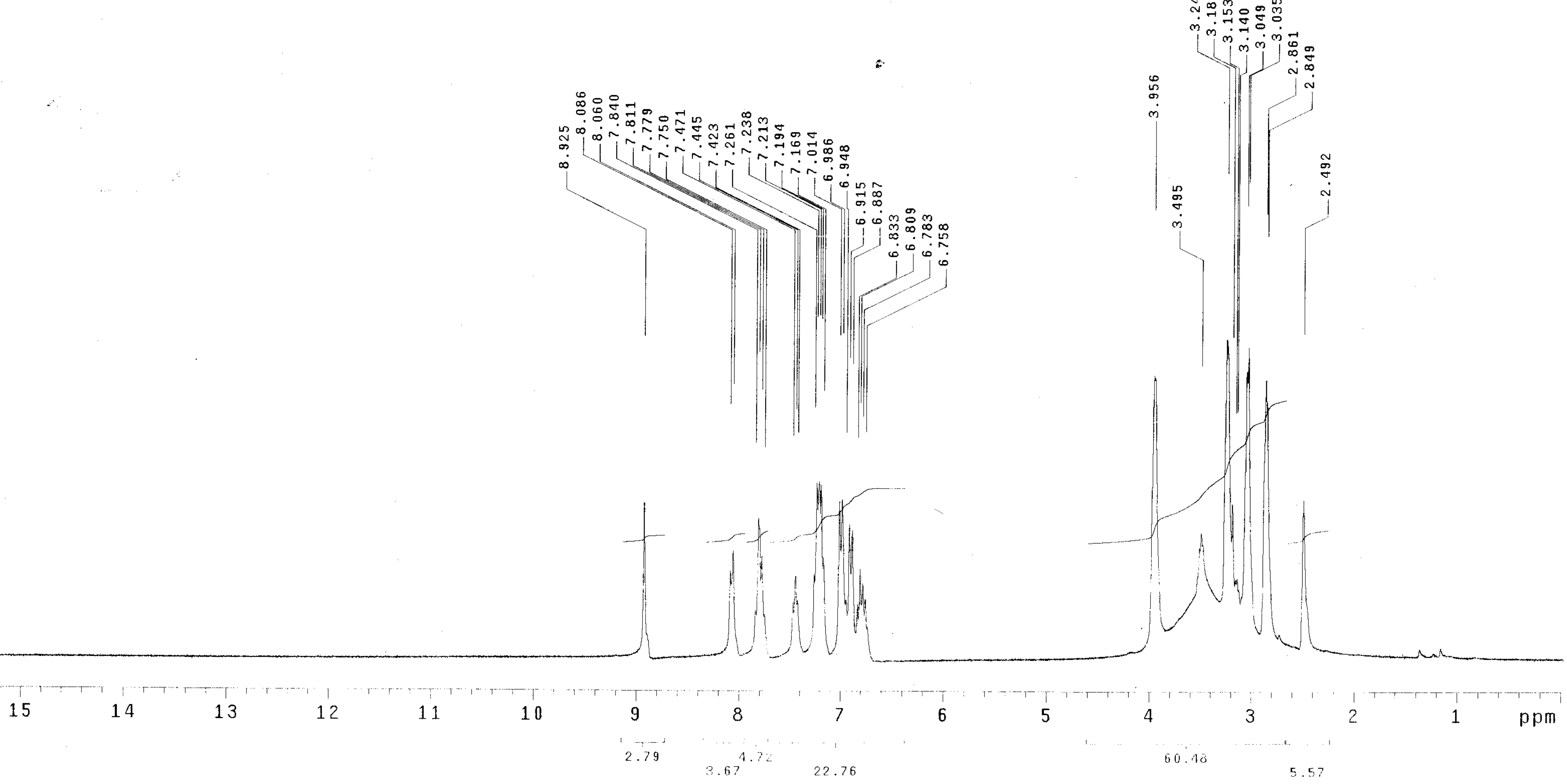

STANDARD 1H OBSERVE

Pulse Sequence: s2pul

Solvent: DMSO
Ambient temperature
GEMINI-200 "NMR"

Relax. delay 1.000 sec
Pulse 39.4 degrees
Acq. time 1.998 sec
Width 4500.0 Hz
67 repetitions
OBSERVE H1, 199.9784953 MHz
DATA PROCESSING
FT size 32768
Total time 11 min, 11 sec

Micro Analytical Center
جامعة القاهرة
التحاليل

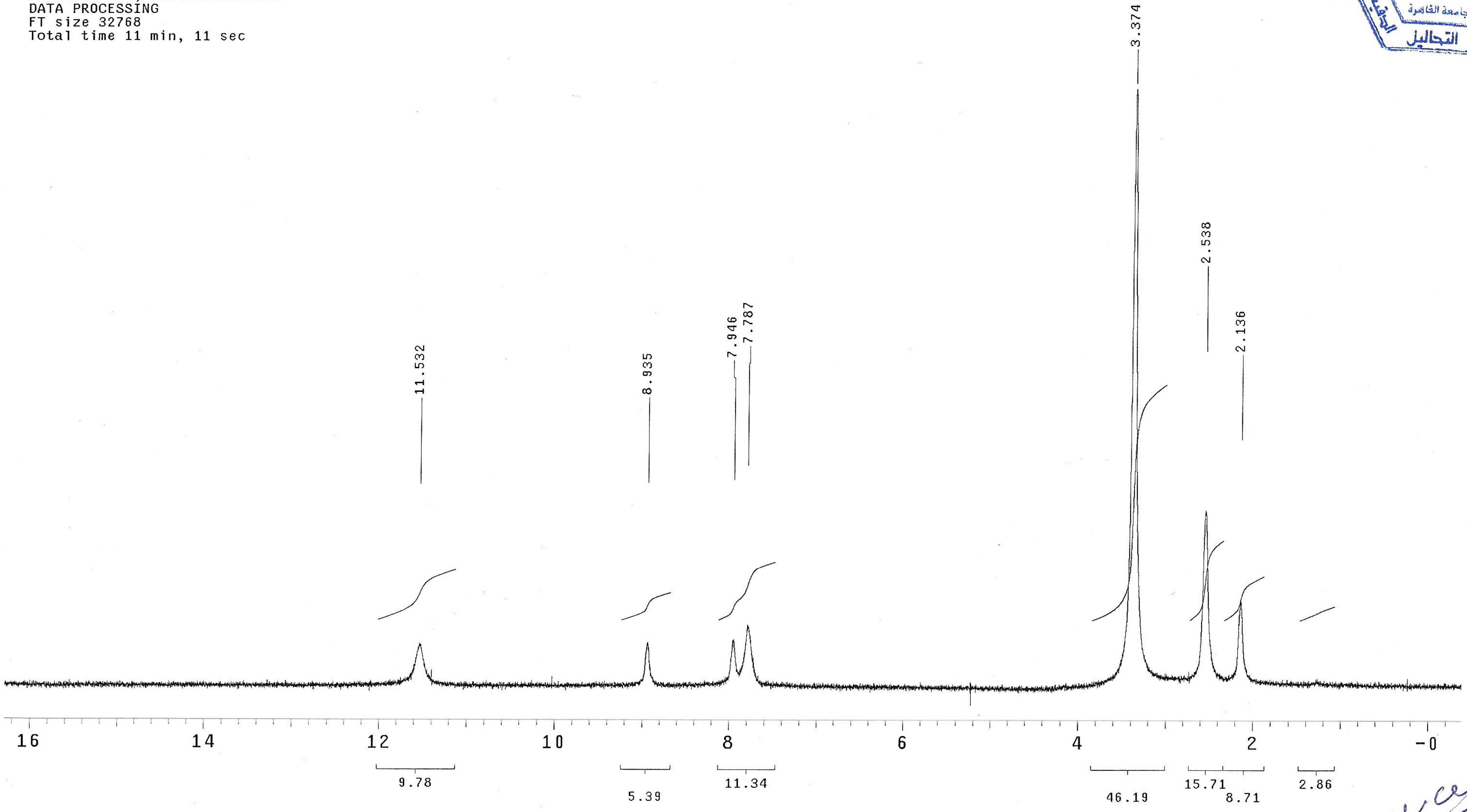

STANDARD 1H OBSERVE

Pulse Sequence: s2pul

Solvent: DMSO
Ambient temperature
GEMINI-200 "NMR"

Relax. delay 1.000 sec
Pulse 39.4 degrees
Acq. time 1.995 sec
Width 4500.0 Hz
108 repetitions
OBSERVE H1, 199.9784999 MHz
DATA PROCESSING
FT size 32768
Total time 11 min, 11 sec

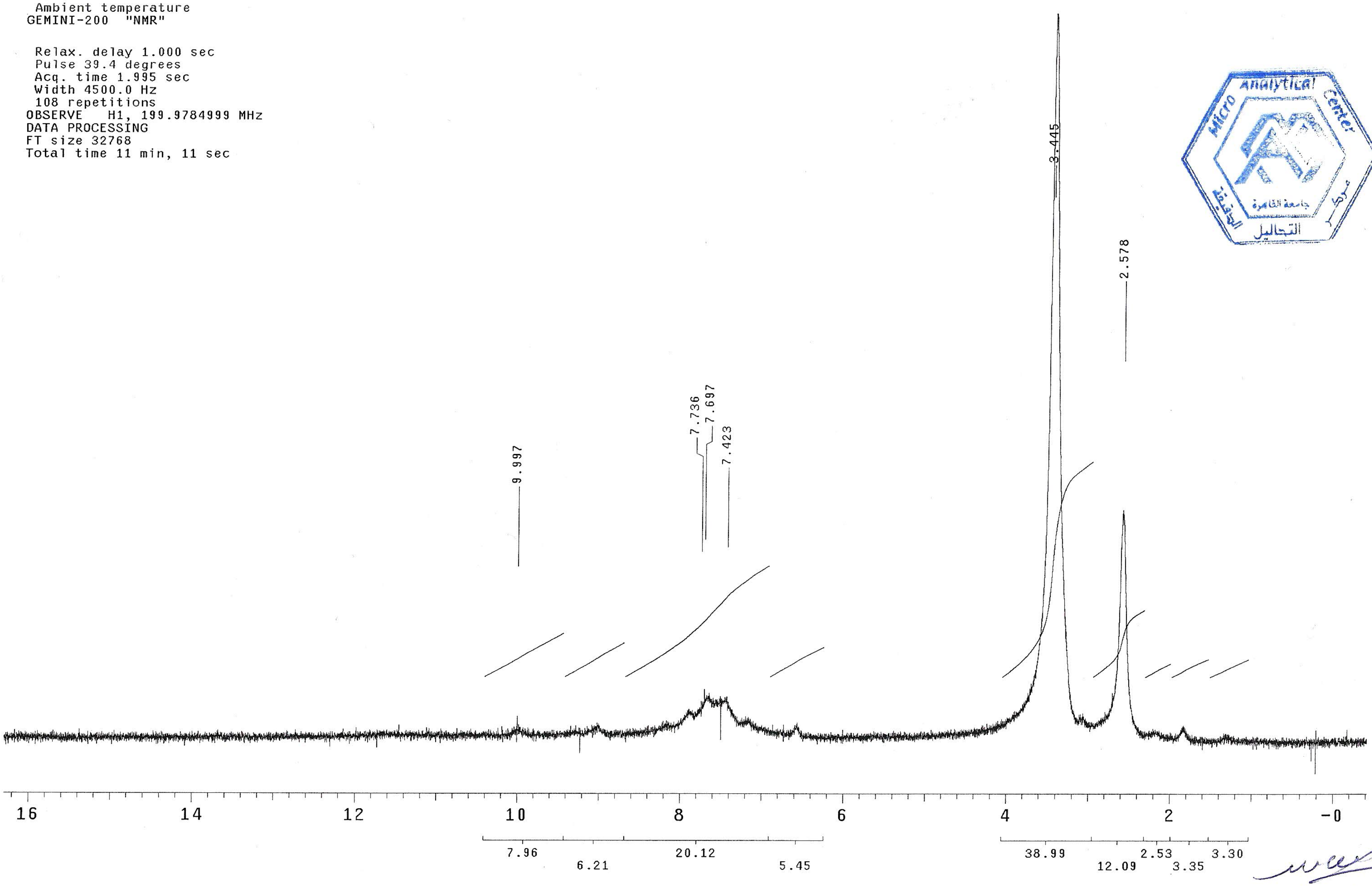

Micro Analytical Center
جامعة القاهرة
مركز التحاليل الدقيقة

SawsanAhmad-SN-DMSO-H

Pulse Sequence: s2pul

Solvent: DMSO
Temp. 30.0 C / 303.1 K
Mercury-300BB "NMR300"

Relax. delay 1.000 sec
Pulse 74.1 degrees
Acq. time 4.004 sec
Width 8000.0 Hz
11 repetitions
OBSERVE H1, 300.0687855 MHz
DATA PROCESSING
FT size 65536
Total time 19 min, 19 sec

8 NMR

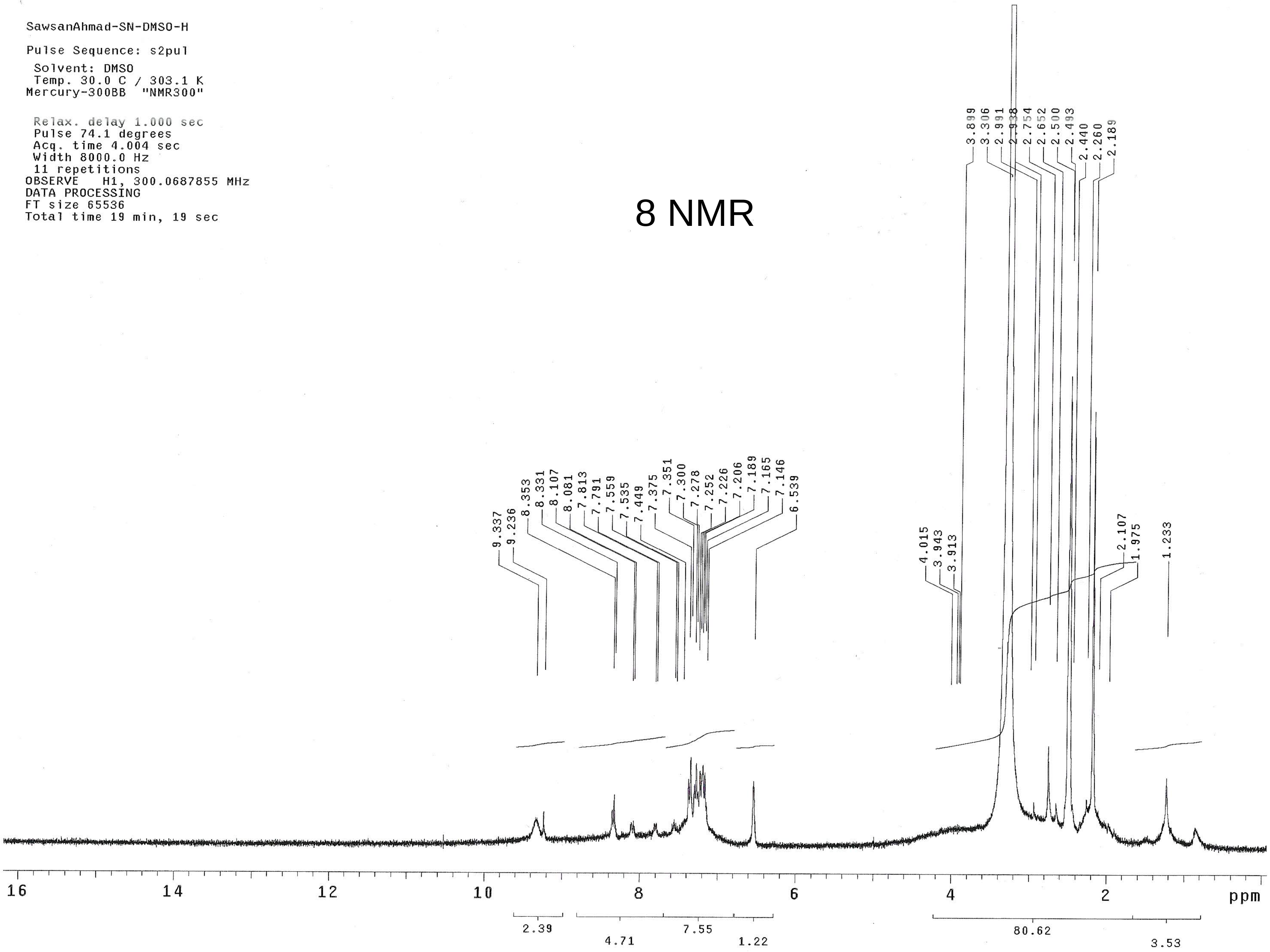

Cairo University
Micro Analytical Center

DI Analysis
Shimadzu Qp-2010 Plus

Sample Information

Analyzed by	: A.GABR
Analyzed	: 1/26/2011 1:16:55 PM
Sample Name	: S2
Sample ID	:
Customer Name	: SAWSAN AHMAD SHWKYVial # : $Vial #
Data File	: C:\GCMSsolution\Data\Project1\S2.QGD
Org Data File	: C:\GCMSsolution\Data\Project1\S2.QGD
Method File	: (Untitled)
Org Method File	: (Untitled)
Report File	:
Tuning File	: C:\GCMSsolution\System\Tune1_default.qgt
$EndIf$Modified by	: A.GABR
Modified	: 1/26/2011 1:26:50 PM

Method

==== Analytical Line 1 ====

IonSourceTemp	:200.00 °C
[MS Table]	
--Group 1 - Event 1--	
Start Time	:0.00min
End Time	:10.00min
ACQ Mode	:Scan
Event Time	:0.50sec
Scan Speed	: 769
Start m/z	:50.00
End m/z	:400.00
Electron Voltage	: 70 eV
Ionization Mode	: EI

C:\GCMSsolution\Data\Project1\S2.QGD

Chromatogram S2 C:\GCMSsolution\Data\Project1\S2.QGD

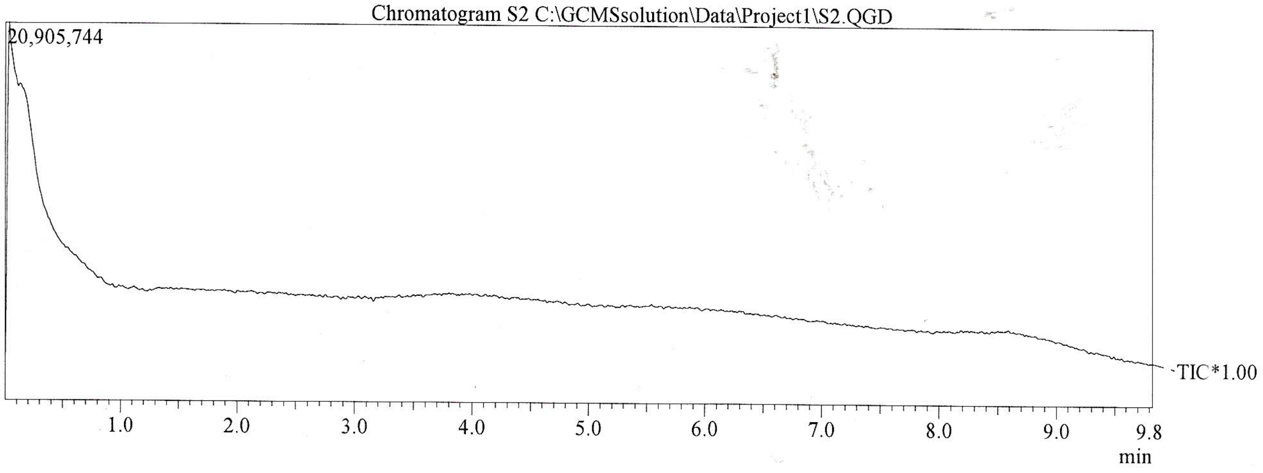

Spectrum

Line#:1 R.Time:7.2(Scan#:860)
MassPeaks:220
RawMode:Single 7.2(860) BasePeak:55(131856)
BG Mode:None Group 1 - Event 1

2c mass

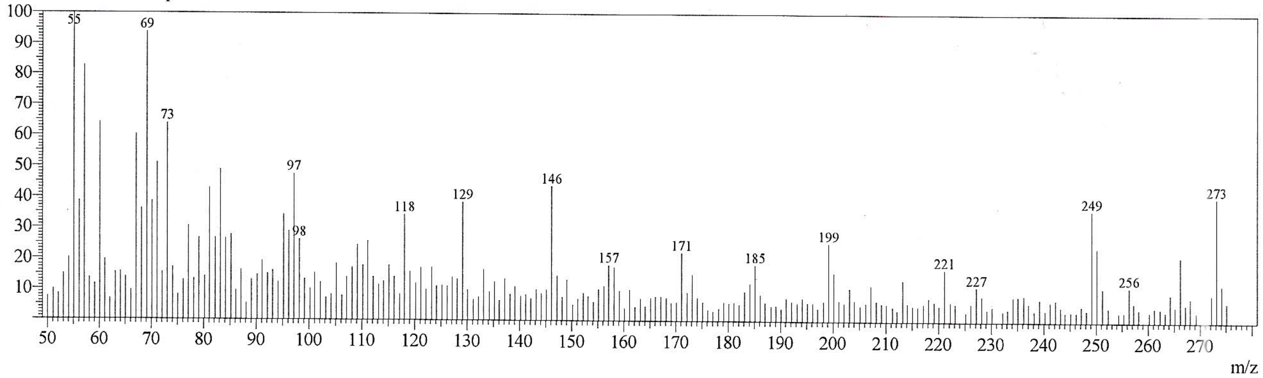

Mass Table
Line#:1 R.Time:7.2(Scan#:860)
MassPeaks:220
RawMode:Single 7.2(860) BasePeak:55(131856)
BG Mode:None Group 1 - Event 1

#	m/z	Abs. Int	Rel. Int.	#	m/z	Abs. Int	Rel. Int.	#	m/z	Abs. Int	Rel. Int.
1	50.00	9841	7.46	4	53.00	19657	14.91	7	56.05	51038	38.71
2	51.05	13057	9.90	5	54.05	26559	20.14	8	57.05	109239	82.85
3	52.05	11157	8.46	6	55.05	131856	100.00	9	58.05	17934	13.60

Cairo University
Micro Analytical Center

DI Analysis
Shimadzu Qp-2010 Plus

Sample Information

Analyzed by	: O.HY
Analyzed	: 20/04/2010 11:55:09 م
Sample Name	: SA8
Sample ID	:
Customer Name	: SAWSAN AHMED Vial # : $Vial #
Data File	: C:\GCMSsolution\Data\Project1\SA8.QGD
Org Data File	: C:\GCMSsolution\Data\Project1\SA8.QGD
Method File	: (Untitled)
Org Method File	: (Untitled)
Report File	:
Tuning File	: C:\GCMSsolution\System\Tune1_default.qgt
$EndIf$Modified by	: O.HY
Modified	: 20/04/2010 11:58:44 م

Method

=====Analytical Line 1 =====

IonSourceTemp	:200.00 °C
[MS Table]	
--Group 1 - Event 1--	
Start Time	:0.50min
End Time	:10.00min
ACQ Mode	:Scan
Event Time	:0.50sec
Scan Speed	: 526
Start m/z	:50.00
End m/z	:300.00
Electron Voltage	: 70 eV
Ionization Mode	: EI

C:\GCMSsolution\Data\Project1\SA8.QGD

Chromatogram SA8 C:\GCMSsolution\Data\Project1\SA8.QGD

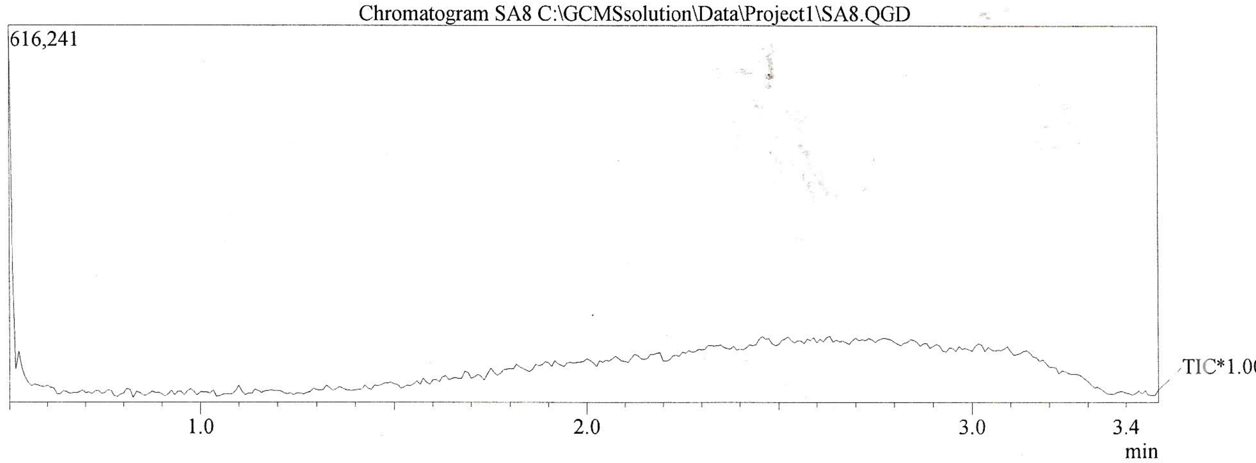

Spectrum

Line#:1 R.Time:2.7(Scan#:264)
MassPeaks:52
RawMode:Single 2.7(264) BasePeak114(5956)
BG Mode:None Group 1 - Event 1

2e mass

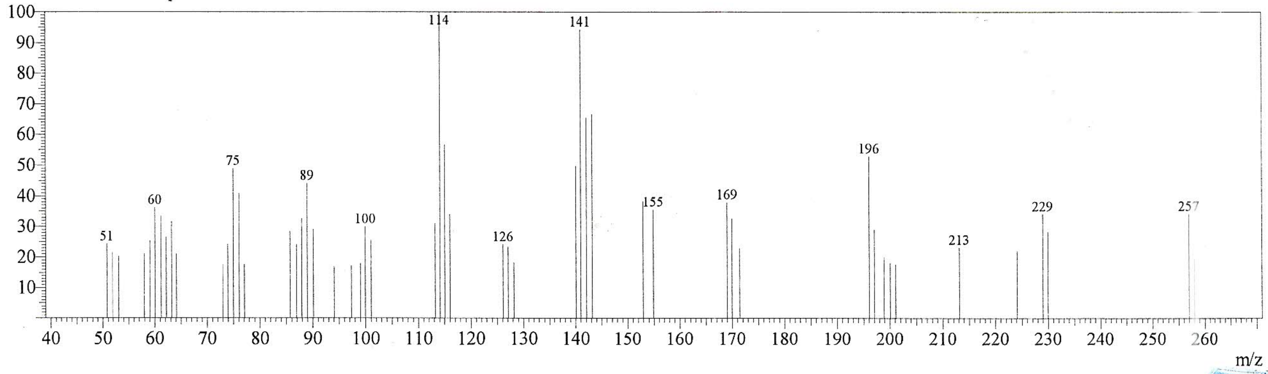

Mass Table
Line#:1 R.Time:2.7(Scan#:264)
MassPeaks:52
RawMode:Single 2.7(264) BasePeak:114(5956)
BG Mode:None Group 1 - Event 1

#	m/z	Abs. In	Rel. Int.	#	m/z	Abs. In	Rel. Int.	#	m/z	Abs. In	Rel. Int.
1	50.75	1448	24.31	4	57.95	1254	21.05	7	61.20	1999	33.56
2	51.85	1277	21.44	5	59.05	1505	25.27	8	62.15	1580	26.53
3	53.10	1206	20.25	6	60.05	2157	36.22	9	63.20	1881	31.58

Cairo University
Micro Analytical Center

DI Analysis
Shimadzu Qp-2010 Plus

Sample Information

Analyzed by	: A.GABR
Analyzed	: 1/27/2011 10:04:10 AM
Sample Name	: S12
Sample ID	:
Customer Name	: SAWSAN AHMAD SHWKYVial # : $Vial #
Data File	: C:\GCMSsolution\Data\Project1\S12.QGD
Org Data File	: C:\GCMSsolution\Data\Project1\S12.QGD
Method File	: (Untitled)
Org Method File	: (Untitled)
Report File	:
Tuning File	: C:\GCMSsolution\System\Tune1_default.qgt
$EndIf$Modified by	: A.GABR
Modified	: 1/27/2011 10:10:40 AM

Method

===== Analytical Line 1 =====

IonSourceTemp	:200.00 °C
[MS Table]	
--Group 1 - Event 1--	
Start Time	:0.00min
End Time	:10.00min
ACQ Mode	:Scan
Event Time	:0.50sec
Scan Speed	: 625
Start m/z	:50.00
End m/z	:350.00
Electron Voltage	: 70 eV
Ionization Mode	: EI

C:\GCMSsolution\Data\Project1\S12.QGD

Chromatogram S12 C:\GCMSsolution\Data\Project1\S12.QGD

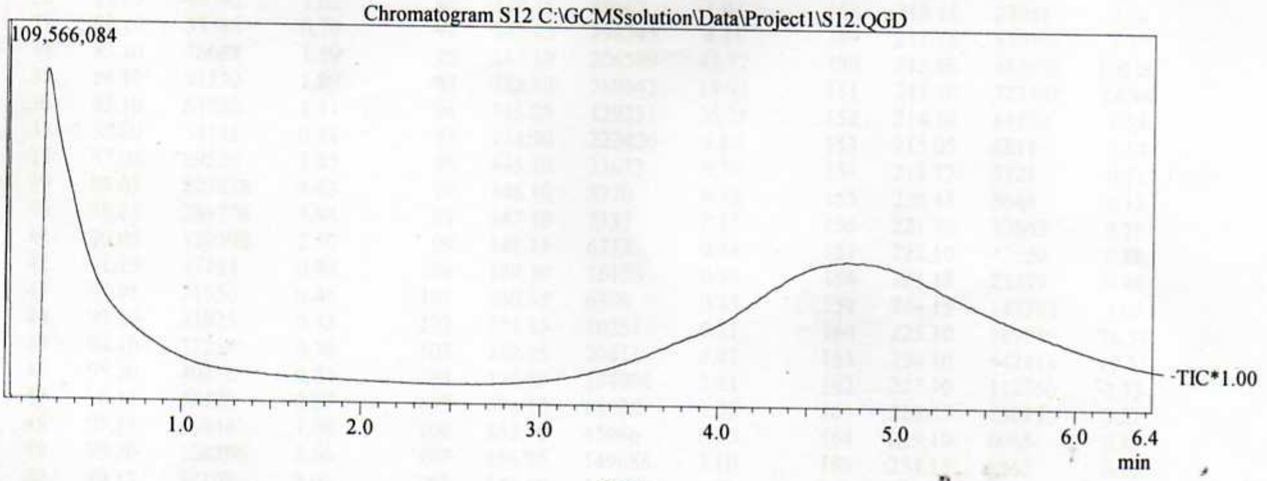

Spectrum

Line#:1 R.Time:5.2(Scan#:628)
MassPeaks:183
RawMode:Single 5.2(628) BasePeak:212(4830583)
BG Mode:None Group 1 - Event 1

3c mass

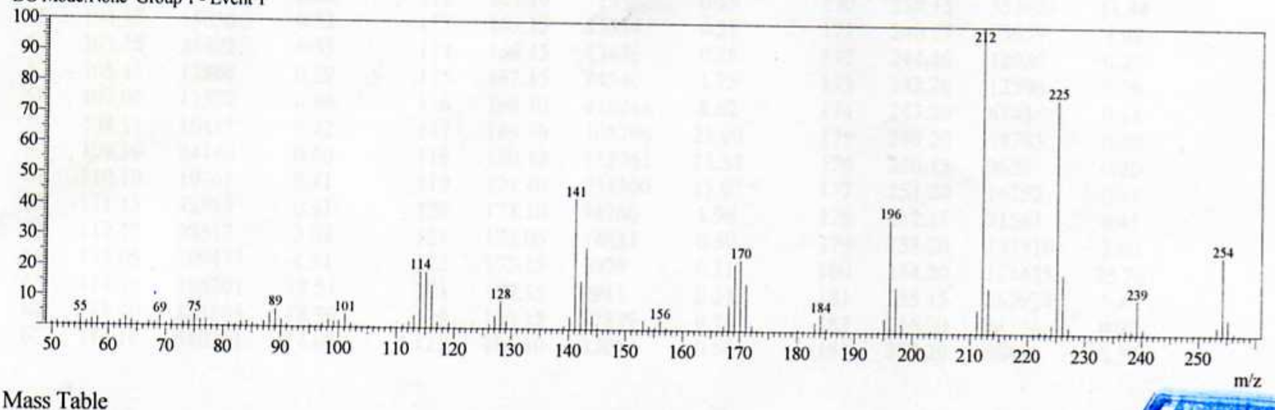

Mass Table
Line#:1 R.Time:5.2(Scan#:628)
MassPeaks:183
RawMode:Single 5.2(628) BasePeak:212(4830583)
BG Mode:None Group 1 - Event 1

#	m/z	Abs. Int	Rel. Int.	#	m/z	Abs. Int	Rel. Int.	#	m/z	Abs. Int	Rel. Int.
1	50.00	40831	0.85	4	52.95	30779	0.64	7	56.05	61824	1.28
2	50.95	58507	1.21	5	54.05	34344	0.71	8	57.05	105163	2.18
3	52.00	36137	0.75	6	55.00	159698	3.31	9	58.05	128809	2.67

Cairo University
Micro Analytical Center

DI Analysis
Shimadzu Qp-2010 Plus

Sample Information

Analyzed by	: A.GABR
Analyzed	: 1/27/2011 10:45:02 AM
Sample Name	: S77
Sample ID	:
Customer Name	: SAWSAN AHMAD SHWKYVial # : $Vial #
Data File	: C:\GCMSsolution\Data\Project1\S77.QGD
Org Data File	: C:\GCMSsolution\Data\Project1\S77.QGD
Method File	: (Untitled)
Org Method File	: (Untitled)
Report File	:
Tuning File	: C:\GCMSsolution\System\Tune1_default.qgt
$EndIf$Modified by	: A.GABR
Modified	: 1/27/2011 10:50:00 AM

Method

==== Analytical Line 1 ====

IonSourceTemp	:200.00 °C
[MS Table]	
--Group 1 - Event 1--	
Start Time	:0.00min
End Time	:10.00min
ACQ Mode	:Scan
Event Time	:0.50sec
Scan Speed	: 769
Start m/z	:50.00
End m/z	:400.00
Electron Voltage	: 70 eV
Ionization Mode	: EI

C:\GCMSsolution\Data\Project1\S77.QGD

Chromatogram S77 C:\GCMSsolution\Data\Project1\S77.QGD

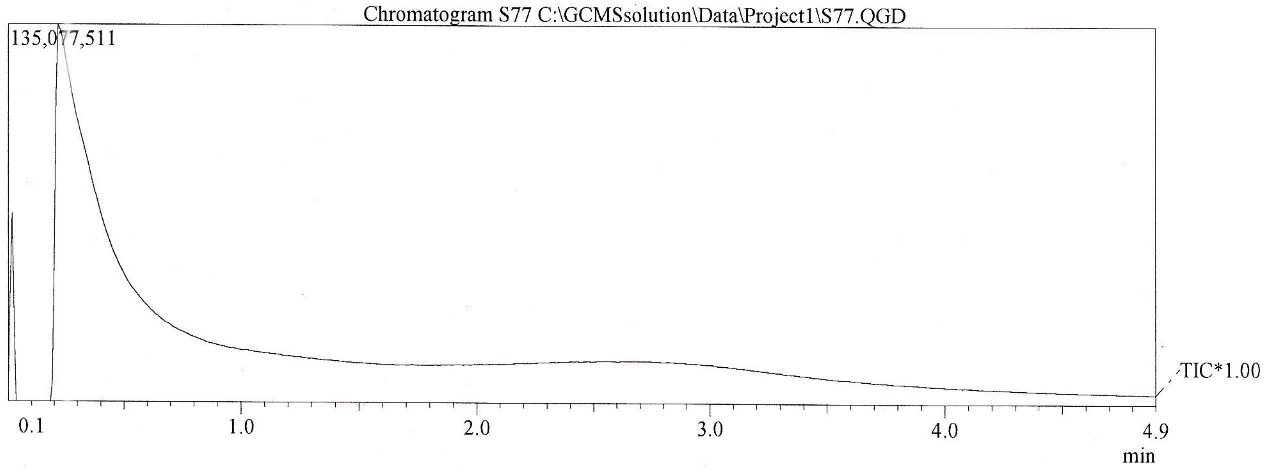

Spectrum

Line#:1 R.Time:2.9(Scan#:354)
MassPeaks:247
RawMode:Single 2.9(354) BasePeak:57(198704)
BG Mode:3.7(442) Group 1 - Event 1

3d mass

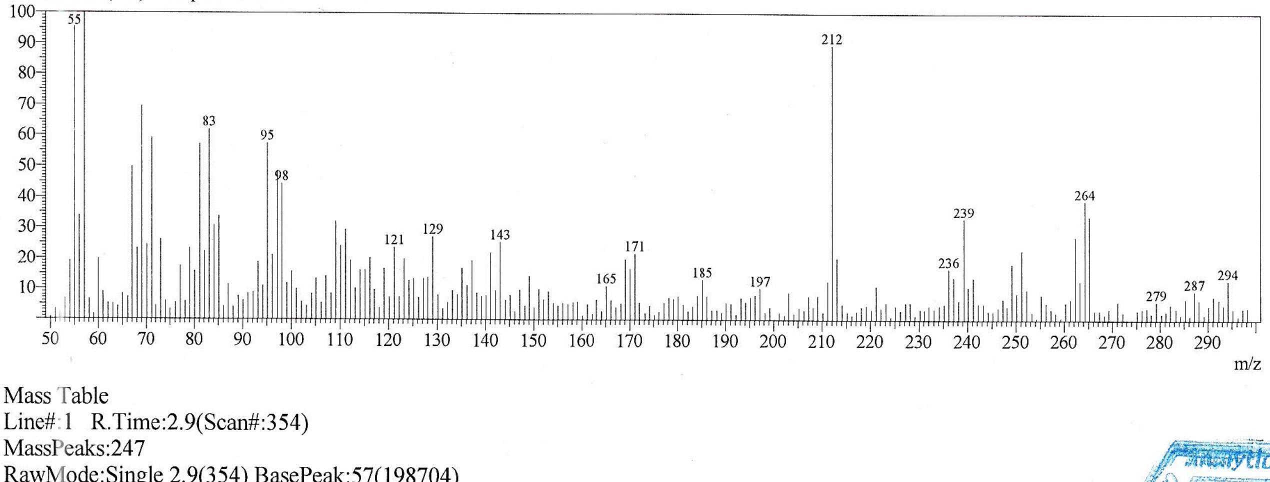

Mass Table
Line#:1 R.Time:2.9(Scan#:354)
MassPeaks:247
RawMode:Single 2.9(354) BasePeak:57(198704)
BG Mode:3.7(442) Group 1 - Event 1

#	m/z	Abs. In	Rel. Int.	#	m/z	Abs. In	Rel. Int.	#	m/z	Abs. In	Rel. Int.
1	50.00	1417	0.71	4	53.05	13473	6.78	7	56.05	67287	33.86
2	51.00	6724	3.38	5	54.05	37930	19.09	8	57.05	198704	100.00
3	52.00	4906	2.47	6	55.05	189656	95.45	9	58.05	12967	6.53

Cairo University
Micro Analytical Center

DI Analysis
Shimadzu Qp-2010 Plus

Sample Information

Analyzed by	: A.GABR
Analyzed	: 12/26/2010 4:17:54 PM
Sample Name	: S76
Sample ID	:
Customer Name	: SAWSAN AHMED SHAWKIVial # : $Vial #
Data File	: C:\GCMSsolution\Data\Project1\S76.QGD
Org Data File	: C:\GCMSsolution\Data\Project1\S76.QGD
Method File	: (Untitled)
Org Method File	: (Untitled)
Report File	:
Tuning File	: C:\GCMSsolution\System\Tune1_default.qgt
$EndIfSModified by	: A.GABR
Modified	: 12/26/2010 4:21:01 PM

Method

==== Analytical Line 1 ====

IonSourceTemp	:200.00 °C
[MS Table]	
--Group 1 - Event 1--	
Start Time	:0.50min
End Time	:10.00min
ACQ Mode	:Scan
Event Time	:0.50sec
Scan Speed	: 526
Start m/z	:50.00
End m/z	:300.00
Electron Voltage	: 70 eV
Ionization Mode	: EI

C:\GCMSsolution\Data\Project1\S76.QGD

Chromatogram S76 C:\GCMSsolution\Data\Project1\S76.QGD

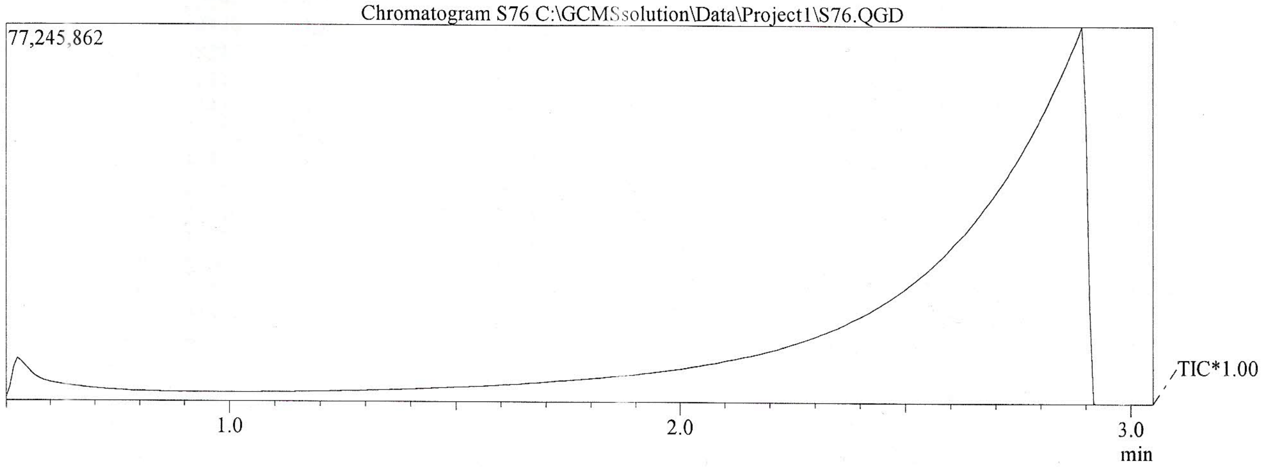

Spectrum

Line#:1 R.Time:2.8(Scan#:281)
MassPeaks:228
RawMode:Single 2.8(281) BasePeak:226(6305619)
BG Mode:1.7(145) Group 1 - Event 1

3k mass

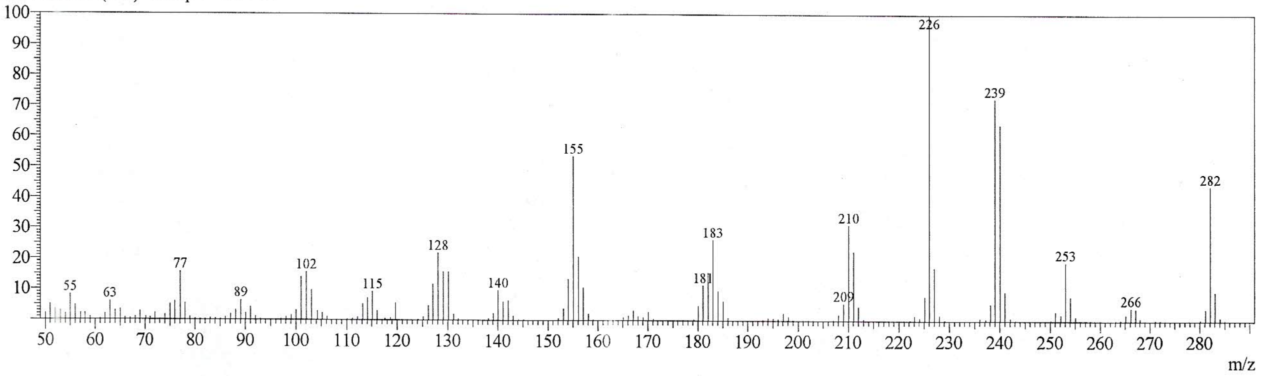

Mass Table
Line#:1 R.Time:2.8(Scan#:281)
MassPeaks:228
RawMode:Single 2.8(281) BasePeak:226(6305619)
BG Mode:1.7(145) Group 1 - Event 1

#	m/z	Abs. Int	Rel. Int.	#	m/z	Abs. Int	Rel. Int.	#	m/z	Abs. Int	Rel. Int.
1	50.00	131272	2.08	4	53.00	198140	3.14	7	56.00	299025	4.74
2	51.00	313779	4.98	5	54.05	116913	1.85	8	57.05	140718	2.23
3	52.00	223941	3.55	6	55.00	513523	8.14	9	58.00	139530	2.21

Cairo University
Micro Analytical Center

DI Analysis
Shimadzu Qp-2010 Plus

Sample Information

Analyzed by	: A.GABR
Analyzed	: 1/26/2011 1:37:06 PM
Sample Name	: S22
Sample ID	:
Customer Name	: SAWSAN AHMAD SHWKYVial # : $Vial #
Data File	: C:\GCMSsolution\Data\Project1\S22.QGD
Org Data File	: C:\GCMSsolution\Data\Project1\S22.QGD
Method File	: (Untitled)
Org Method File	: (Untitled)
Report File	:
Tuning File	: C:\GCMSsolution\System\Tune1_default.qgt
$EndIf$Modified by	: A.GABR
Modified	: 1/26/2011 1:42:51 PM

Method

==== Analytical Line 1 ====

IonSourceTemp	:200.00 °C
[MS Table]	
--Group 1 - Event 1--	
Start Time	:0.00min
End Time	:10.00min
ACQ Mode	:Scan
Event Time	:0.50sec
Scan Speed	: 769
Start m/z	:50.00
End m/z	:400.00
Electron Voltage	: 70 eV
Ionization Mode	: EI

C:\GCMSsolution\Data\Project1\S22.QGD

Chromatogram S22 C:\GCMSsolution\Data\Project1\S22.QGD

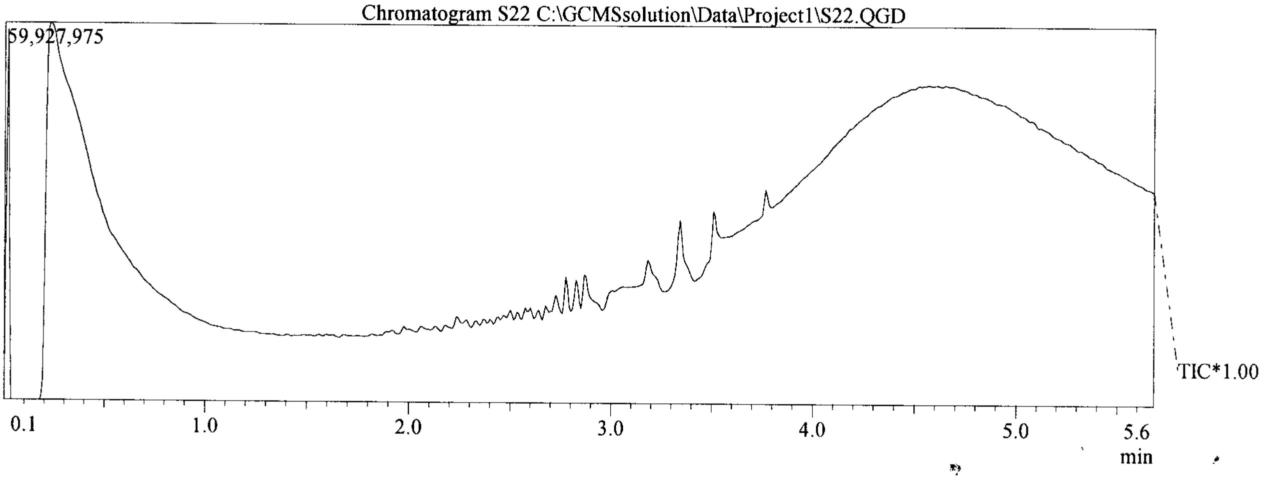

Spectrum

Line#:1 R.Time:5.5(Scan#:662)
MassPeaks:237
RawMode:Single 5.5(662) BasePeak:255(1531490)
BG Mode:1.9(226) Group 1 - Event 1

4b mass

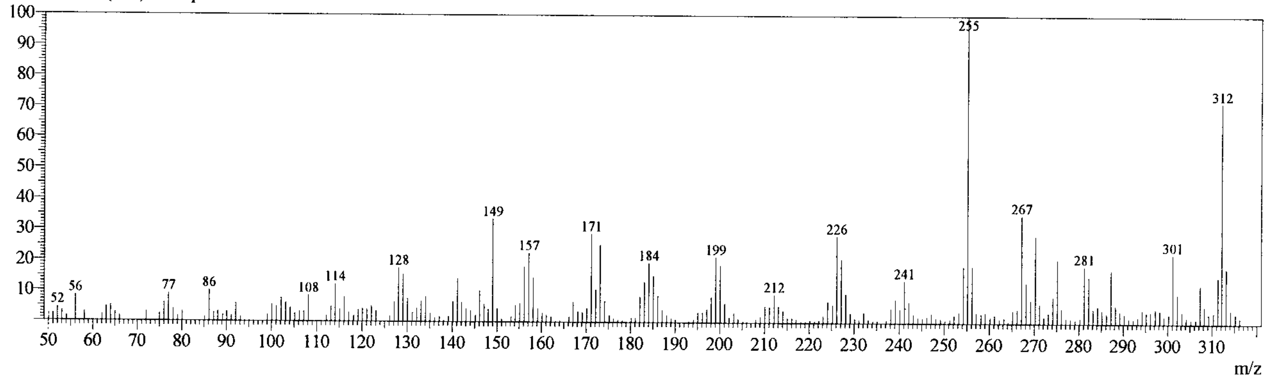

Mass Table
Line#:1 R.Time:5.5(Scan#:662)
MassPeaks:237
RawMode:Single 5.5(662) BasePeak:255(1531490)
BG Mode:1.9(226) Group 1 - Event 1

#	m/z	Abs. Int.	Rel. Int.	#	m/z	Abs. Int.	Rel. Int.	#	m/z	Abs. Int.	Rel. Int.
1	50.00	37316	2.44	4	53.00	50980	3.33	7	58.05	45940	3.00
2	51.00	40002	2.61	5	54.05	25834	1.69	8	62.05	33172	2.17
3	52.00	68157	4.45	6	56.05	127419	8.32	9	63.05	71624	4.68

Cairo University
Micro Analytical Center

DI Analysis
Shimadzu Qp-2010 Plus

Sample Information

Analyzed by : A.GABR
Analyzed : 1/27/2011 10:21:08 AM
Sample Name : SN
Sample ID :
Customer Name : SAWSAN AHMAD SHWKYVial # : $Vial #
Data File : C:\GCMSsolution\Data\Project1\SN.QGD
Org Data File : C:\GCMSsolution\Data\Project1\SN.QGD
Method File : (Untitled)
Org Method File : (Untitled)
Report File :
Tuning File : C:\GCMSsolution\System\Tune1_default.qgt
$EndIf$Modified by : A.GABR
Modified : 1/27/2011 10:26:14 AM

Method

==== Analytical Line 1 ====
IonSourceTemp :200.00 °C
[MS Table]
--Group 1 - Event 1--
Start Time :0.00min
End Time :10.00min
ACQ Mode :Scan
Event Time :0.50sec
Scan Speed : 526
Start m/z :50.00
End m/z :300.00

Electron Voltage : 70 eV
Ionization Mode : EI

C:\GCMSsolution\Data\Project1\SN.QGD

Chromatogram SN C:\GCMSsolution\Data\Project1\SN.QGD

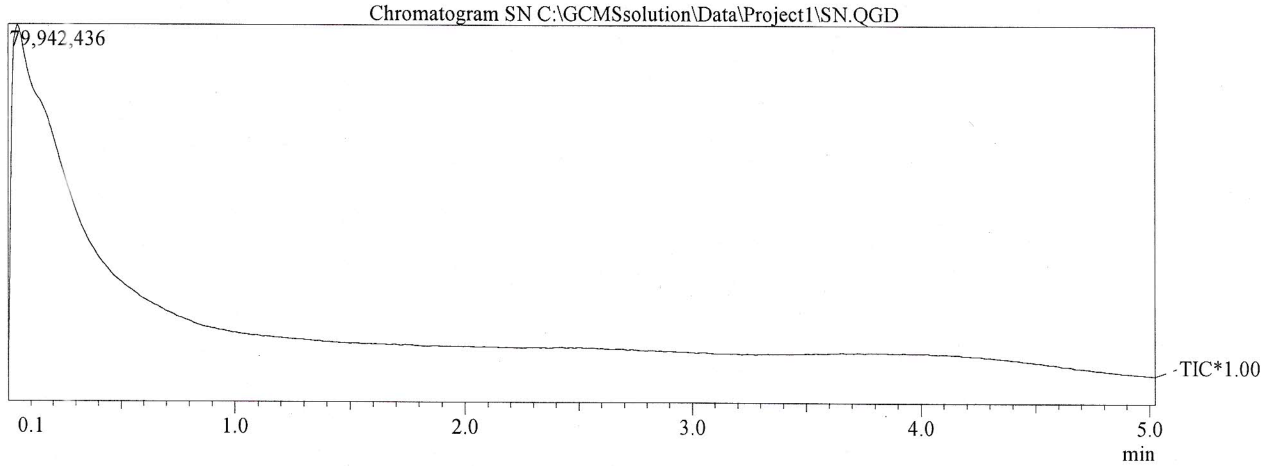

Spectrum

Line#:1 R.Time:4.7(Scan#:569)
MassPeaks:166
RawMode:Single 4.7(569) BasePeak:211(252156)
BG Mode:None Group 1 - Event 1

8 mass

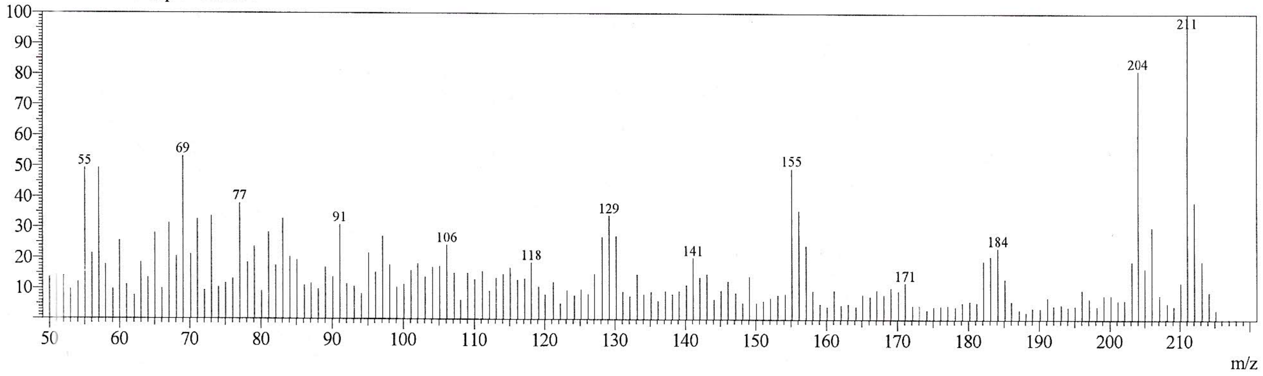

Mass Table
Line#:1 R.Time:4.7(Scan#:569)
MassPeaks:166
RawMode:Single 4.7(569) BasePeak:211(252156)
BG Mode:None Group 1 - Event 1

#	m/z	Abs. Int	Rel. Int.	#	m/z	Abs. Int	Rel. Int.	#	m/z	Abs. Int	Rel. Int.
1	50.00	34482	13.67	4	53.00	24615	9.76	7	56.05	54086	21.45
2	51.00	36159	14.34	5	54.05	30569	12.12	8	57.05	124408	49.34
3	52.00	35329	14.01	6	55.05	124168	49.24	9	58.00	44810	17.77

Peak Find - Memory-25

3a IR

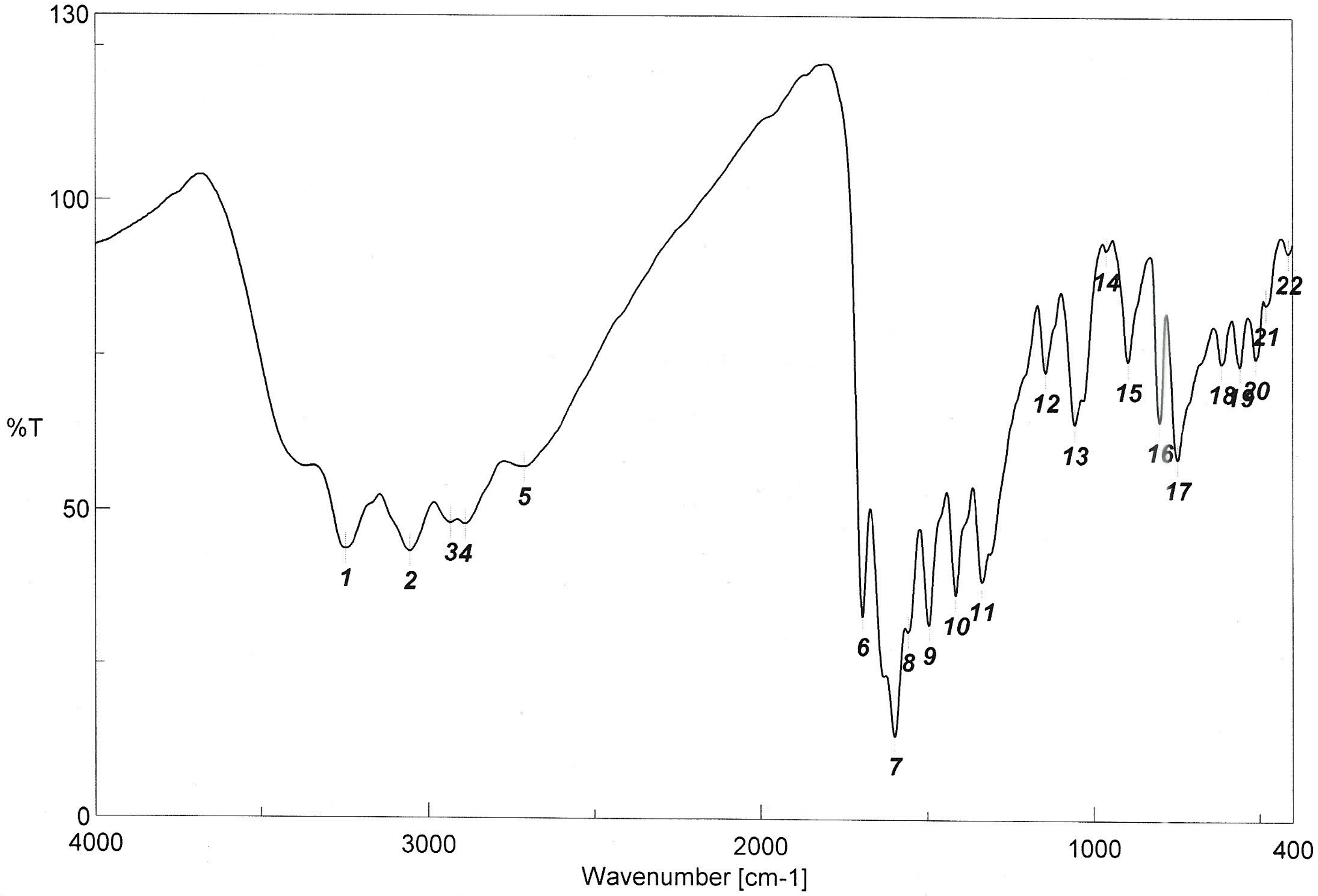

[Comments]

Sample name	S11
Comment	13-1 -2011
User	IR
Division	IR
Company	Micro Analytical Center

[Result of Peak Picking]

No.	Position	Intensity	No.	Position	Intensity	No.	Position	Intensity
1	3247.54	43.7053	2	3055.66	43.2979	3	2933.2	47.9756
4	2888.84	47.8098	5	2712.39	57.052	6	1694.16	32.9696
7	1597.73	13.6103	8	1557.24	30.488	9	1495.53	31.59
10	1415.49	36.4892	11	1336.43	38.7822	12	1145.51	72.6198
13	1057.76	64.211	14	962.305	92.2309	15	896.737	74.4428
16	800.314	64.6733	17	747.281	58.65	18	613.252	74.1274
19	558.291	73.7096	20	511.044	74.9094	21	480.188	83.6244
22	412.692	91.9062						

Micro Analytical Center
جامعة القاهرة
مركز التحاليل الدقيقة

Peak Find - Memory-54

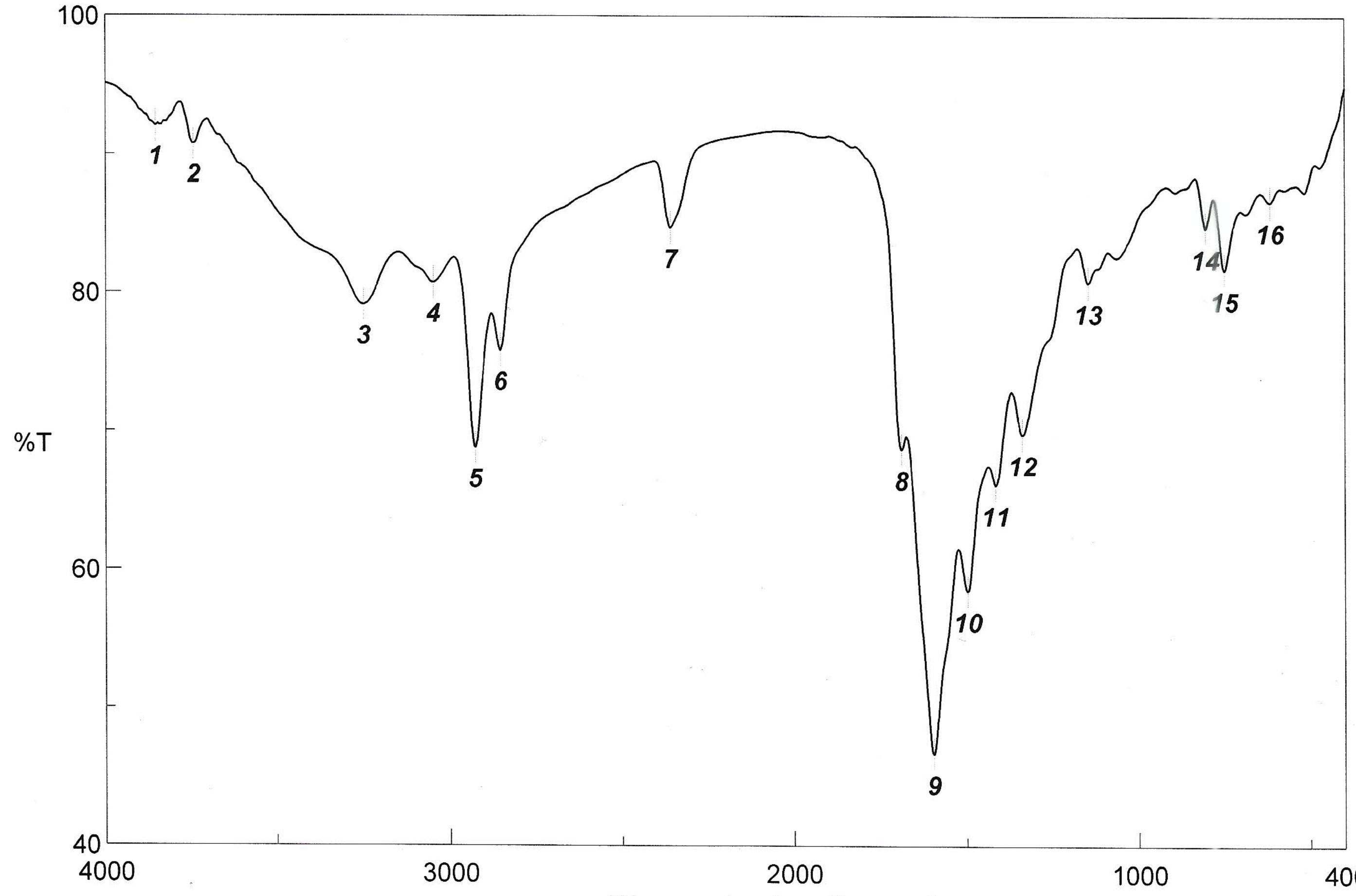

[Comments]

Sample name	S77
Comment	13-1 -2011
User	IR
Division	IR
Company	Micro Analytical Center

[Result of Peak Picking]

No.	Position	Intensity	No.	Position	Intensity	No.	Position	Intensity
1	3853.08	92.0621	2	3742.19	90.7364	3	3250.43	79.1294
4	3048.91	80.7172	5	2927.41	68.7876	6	2854.13	75.8298
7	2358.52	84.6932	8	1688.37	68.6944	9	1595.81	46.693
10	1497.45	58.4831	11	1416.46	66.1661	12	1338.36	69.804
13	1147.44	80.7786	14	806.099	84.7221	15	751.138	81.695
16	617.109	86.5951						

Micro Analytical Center

Peak Find - Memory-10

5a IR

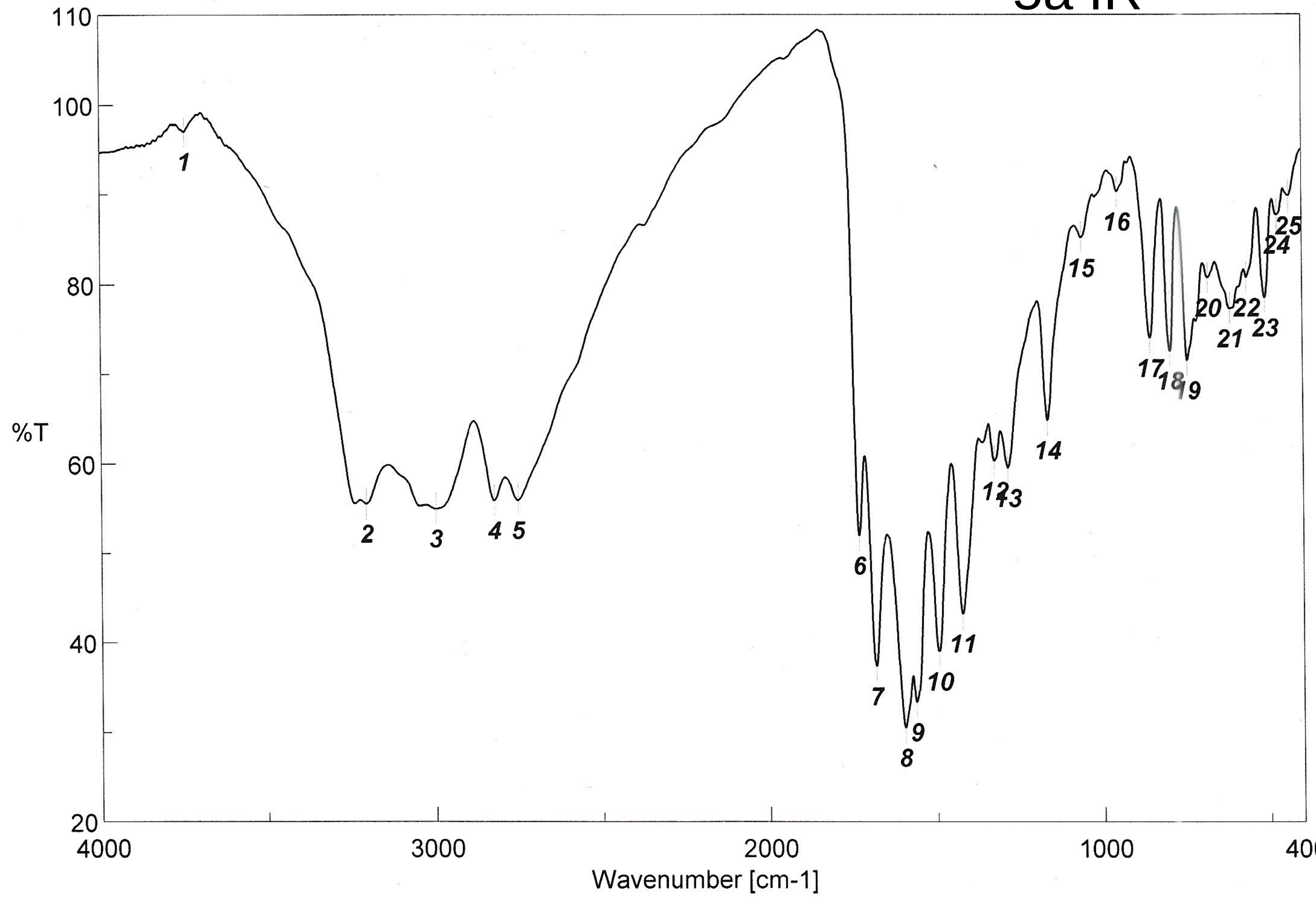

[Comments]

Sample name	SA5
Comment	13-1 -2011
User	IR
Division	IR
Company	Micro Analytical Center

[Result of Peak Picking]

No.	Position	Intensity	No.	Position	Intensity	No.	Position	Intensity
1	3743.15	96.981	2	3206.08	55.5104	3	2999.73	54.98
4	2824.24	55.8855	5	2753.85	55.9382	6	1730.8	51.9871
7	1681.62	37.4005	8	1595.81	30.5522	9	1562.06	33.4161
10	1494.56	39.0347	11	1424.17	43.2571	12	1326.79	60.3163
13	1287.25	59.5506	14	1167.69	64.8578	15	1063.55	85.194
16	955.555	90.2672	17	858.168	74.028	18	798.385	72.6001
19	747.281	71.5367	20	682.677	80.771	21	617.109	77.3383
22	566.969	80.7878	23	512.008	78.5173	24	476.331	87.6968
25	440.655	89.8002						

Micro Analytical Center
